# Structure‐Based Design, Synthesis and Biological Evaluation of Bis‐Tetrahydropyran Furan Acetogenin Mimics Targeting the Trypanosomatid F1 Component of ATP Synthase

**DOI:** 10.1002/ejoc.201900541

**Published:** 2019-05-29

**Authors:** Marija K. Zacharova, Lindsay B. Tulloch, Eoin R. Gould, Andrew L. Fraser, Elizabeth F. King, Stefanie K. Menzies, Terry K. Smith, Gordon J. Florence

**Affiliations:** ^1^ EaStCHEM School of Chemistry and School of Biology Biomedical Science Research Complex University of St Andrews North Haugh St Andrews KY16 9ST UK

**Keywords:** Trypanosomatid, Drug design, Biological activity, FoF1‐ATP synthase, Homogeneous catalysis

## Abstract

The protozoan parasites *Trypanosoma brucei, Trypanosoma cruzi* and *Leishmania spp*. are responsible for the severely debilitating neglected Tropical diseases of African sleeping sickness, Chagas disease and leishmaniasis, respectively. As part of our ongoing programme exploring the potential of simplified analogues of the acetogenin chamuvarinin we identified the *T. brucei* FoF1‐ATP synthase as a target of our earlier triazole analogue series. Using computational docking studies, we hypothesised that the central triazole heterocyclic spacer could be substituted for a central 2,5‐substituted furan moiety, thus diversifying the chemical framework for the generation of compounds with greater potency and/or selectivity. Here we report the design, docking, synthesis and biological evaluation of new series of trypanocidal compounds and demonstrate their on‐target inhibitory effects. Furthermore, the synthesis of furans by the modular coupling of alkyne‐ and aldehyde‐THPs to bis‐THP 1,4‐alkyne diols followed by ruthenium/xantphos‐catalysed heterocyclisation described here represents the most complex use of this method of heterocyclisation to date.

## Introduction

Infectious parasitic diseases are one of the major concerns in Developing countries. In particular, the protozoan parasites of the kinetoplastid family are responsible for several debilitating diseases, with *Trypanosoma brucei, Trypanosoma cruzi* and *Leishmania spp*. being responsible for African sleeping sickness (human African trypanosomiasis, HAT), Chagas disease (American trypanosomiasis), and leishmaniasis, respectively.^1^, ^2^, ^3^ All three diseases have a multi‐thousand annual mortality rate, but have been inadequately investigated, mainly due to the limited financial incentive in treating these conditions. The existing treatments have severe side effects and are difficult to administer (usually intravenously) given the challenging healthcare infrastructure in many affected countries.^4^, ^5^, ^6^, ^7^ A number of novel candidate molecules have entered clinical trials, Fexinidazole^8^, ^9^ and SCYX‐7158^10^ currently being in phase II/III trials. Nevertheless, emerging resistance towards existing treatments^11^, ^12^, ^13^ combined with the lack of preventive methods or possibility for vaccination,^14^, ^15^, ^16^ indicate an urgent need for novel, broad‐spectrum and selective trypanocidal treatments, particularly in the light of the ambitious target set by the World Health Organisation (WHO) to eradicate HAT by 2020.^1^

**Figure 1:**
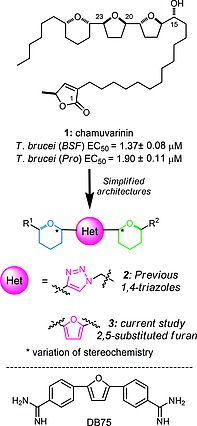
Trypanocidal chamuvarinin, simplified heterocyclic analogues and structure of DB75.

Natural products are a valuable source of unexplored chemical frameworks for the development of new drugs. Chamuvarinin (**1**), originally isolated from the roots of *Uvaria chamae*, which has traditionally been used as a herbal remedy for HAT, and subsequently synthesised in our laboratory,^17^ has low micromolar trypanocidal activity (Figure 1).^18^ We have since described a series of bis‐tetrahydropyran 1,4‐triazole analogues based on the chamuvarinin polyether core and assembled from a pool of readily accessible chiral tetrahydropyran (THP) building blocks linked by different heterocyclic spacers including 1,4‐triazoles of type **2** (Figure 1). Many of these simplified analogues displayed low micromolar trypanocidal activities but more significantly excellent selectivities relative to mammalian cell lines.^19^, ^20^, ^21^, ^22^

Through a series of pull‐down experiments we identified the *T. brucei* FoF1‐ATP synthase as a target for our lead triazole series.^23^ FoF1 is a highly conserved mitochondrial protein consisting of transmembrane (Fo) and matrix‐facing (F1) moieties.^24^, ^25^ F1, which binds our triazole inhibitors, is a heterohexamer consisting of alternating α‐ and β‐subunits with six nucleotide‐binding sites at their interfaces, three of which are catalytic and primarily constitute residues from the β‐subunits, while the remaining three comprising mostly α‐subunit residues have a regulatory function. From our identification and verification of F1 as a target, we proposed that our compounds act as ADP mimics and bind at these nucleotide‐binding sites.

We hypothesised that the triazole spacer unit in our earlier generation inhibitor series (of type **2**, Figure 1) could be substituted for a 2,5‐subsitituted furan moiety, of type **3**, and that this alteration to the chemical framework would reduce conformational flexibility across the heterocyclic array, while maintaining or offering greater potency and/or selectivity. Such bis‐tetrahydropyran 2,5‐substituted furan compounds share a degree of structural similarity with DB75, a bis‐phenyl 2,5‐furan diamidine, which is also reported to inhibit the trypanosomatid FoF1‐ATP synthase.^26^ Herein we undertook computational docking of furans into the recently disclosed crystal structure of F1 from *T. brucei* 25 to identify compounds with potential trypanocidal activities. We then undertook the synthesis of a targeted library of stereochemically complex furan derivatives identified as potential enzyme binders and assessed their biological potential in cytotoxicity assays and F1 assays to confirm their on‐target activities.

## Results and Discussion

**Figure 2:**
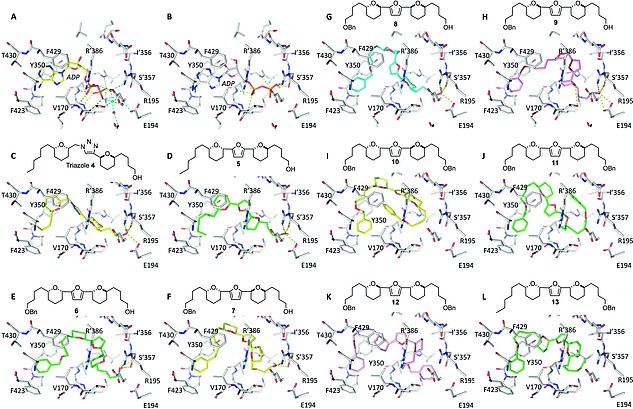
Docked positions of compounds within the *T. brucei* F1 catalytic site. (**A**) Position of bound ADP coordinated with ions in the crystal structure. (**B**) Docked position of ADP in the absence of coordinating ions. The phosphates occupy a slightly different position, but the ribose and adenine occupy the same positions as in the crystal structure. C‐L colour scheme yellow, *syn‐syn*; green, *syn‐anti*; cyan, *anti‐syn*; magenta, *anti‐anti*. (**C‐D**) Docking of lead triazole **4** with Et at R^1^ and OH at R^2^ and furan analogue **5**. The R^2^ OH forms interactions with the phosphate‐binding pocket. (**E‐H**) Furan **6–9** with OBn at R^1^ and OH at R^2^. The additional R^1^ OBn allows π‐stacking in the adenine‐bonding pocket. (**I‐K**) Bis‐OBn furans **10–12**. Substitution of the R^2^ OH for OBn results in the loss of all H‐bonds and potential destabilisation of bound compound. (**L**) Furan **13**, with the OBn at R^1^, flips orientation indicating the phenyl is preferable in the adenine pocket.

To confirm that AutoDock Vina had been set up correctly, and to estimate binding affinity in kcal/mol, ADP and ATP were docked into the catalytic site (corresponding to the ATP‐bound conformation) of F1 from *T. brucei*.^25^, ^27^ Both compounds docked suitably close to the position of ADP detected within the F1 crystal structure (Figure 2A–B), although the β‐phosphate was positioned where the γ‐phosphate of ATP would normally reside, rather than in its own pocket. This is likely a result of Mg^2+^ and water molecules in the crystal structure, which help coordinate the β‐phosphate within its phosphate‐binding pocket, being absent from the docking calculations (since inhibitors can often displace ions to increase binding affinities). On this basis we were satisfied that our modeling methodology had been setup appropriately.

We next docked our lead triazole **4**, which we have established as an F1 inhibitor,^23^ into the same catalytic site of F1 from *T. brucei*. Triazole **4** docked in the same mode as that predicted for bovine F1,^23^ with the THP‐Et side chain bound within the hydrophobic adenine‐binding pocket and the THP‐OH interacting with Glu194, Arg195 and Ser'357 (the prime denoting a residue from the α‐subunit) within the γ‐phosphate‐binding pocket (Figure 2C). This binding mode was predicted to have a similar affinity for the receptor as the substrate and product (Table 1) despite possessing many fewer atoms capable of forming hydrogen bonds than ADP/ATP. However, only the phosphates of ADP and ATP actually form hydrogen bonds with the receptor; the adenine only forms hydrophobic interactions (with Tyr350, Phe429, Val170 and Thr430) while the ribose acts as a heterocyclic linker, thus triazole **4** is able to mimic the substrate(s).

**Table 1:** Compound stereochemistries and docking scores

	Architecture				Dock affinity	Figure
	R^1^	THP	THP	R^2^	(kcal/mol)	
ADP	–	–	–	–	–9.5	2A,B
ATP	–	–	–	–	–9.8	–
Triazole **4**	Et	Syn	Syn	OH	–9.0	2C
Furan **5**	Et	Syn	Anti	OH	–8.9	2D
Furan **6**	OBn	Syn	Anti	OH	–9.7	2E
Furan **7**	OBn	Syn	Syn	OH	–9.7	2F
Furan **8**	OBn	Anti	Syn	OH	–9.1	2G
Furan **9**	OBn	Anti	Anti	OH	–9.9	2H
Furan **10**	OBn	Syn	Syn	OBn	–9.6	2I
Furan **11** ^ [a]^	OBn	Syn	Anti	OBn	–9.7	2J
Furan **12**	OBn	Anti	Anti	OBn	–8.7	2K
Furan **13**	Et	Syn	Anti	OBn	–9.4	2L

Furan **11** can also be represented as OBn‐Anti‐Syn‐OBn.

The bis‐tetrahydropyran furan **5** was designed as an analogue of our lead triazole **4** and alternative substrate mimetic. Both **4** and **5** contain THP‐Et and THP‐OH moieties fused by a heterocycle, but the different orientation of the furan linker could provide alternative stereochemistry for further derivatisation and improved inhibitor design. Docking of furan **5** revealed that it is likely to adopt a similar binding position to triazole **4** in the catalytic site, with the THP‐Et buried within the hydrophobic adenine‐binding pocket and the free alcohol forming hydrogen bonds with Glu194, Arg195 and Ser'357 in the γ‐phosphate‐binding pocket (Figure 2D). Furthermore, the predicted affinity was similar to that of **4** (Table 1).

We postulated that the substitution of the Et group for a benzyl ether (OBn) at R^2^ would lead to an affinity enhancement through the introduction of additional pi‐stacking interactions between Tyr350 and Phe429 in the adenine‐binding pocket, as occurs with ADP and ATP. Docking of **6** (Figure 2E) indicated that the OBn fits within the adenine‐binding pocket without disrupting the binding of the free alcohol within the γ‐phosphate‐binding pocket and lead to a slight improvement in predicted binding affinity. To determine whether different THP stereochemical permutations could optimise binding, *syn‐syn* (furan **7**), *anti‐syn* (furan **8**) and *anti‐anti* (furan **9**) isomers were also docked. All anchored the OBn and OH moieties within the adenine‐ and γ‐phosphate‐binding pockets respectively and through the conformational flexibility of the THP and furan framework to link together (Figure 2E‐H), resulting in similar docking scores.

To determine the importance of the free alcohol on inhibitor potency, the THP‐OH was replaced with THP‐OBn and resulting bis‐THP‐OBn furans (furans **10**, **11** and **12**) were docked into the catalytic site (Figure 2I‐K). For each compound hydrogen bonding with Glu194, Arg195 and Ser'357 in the γ‐phosphate‐binding pocket was completely lost, and despite scoring well in docking, the bound conformations appeared unrealistic, with most of the hydrophobic contacts appearing as intramolecular interactions rather than interactions with the receptor.

As the presence of the OBn at R^1^ in addition to the OBn at R^2^ may make the molecules too large to fit comfortably into the active site, we docked **13** with an OBn at R^1^ and a smaller Et at R^2^ (Figure 2L). Due to the relative similarity of furan core architectures, **13** flipped within the binding site with the OBn, rather than the THP‐Et, docking in the adenine‐binding pocket. The docked position of **13** was similar to that of furans **6–9** with OBn at R^1^ instead of R^2^. This would suggest that the phenyl at R^2^ is likely to improve inhibitor binding over the THP‐Et motif of compounds **4** and **5**. Given the promising docking results for our furan compounds with free alcohols, we decided to synthesise these select examples for biological evaluation, along with the bis‐THP‐OBn compounds as negative controls.

### Synthesis of 2,5‐Substituted Furan Inhibitors

**Scheme 1:**
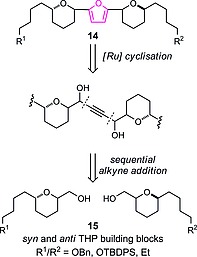
Bis‐tetrahydropyran 2,5‐furan retrosynthesis.

The classical syntheses of furans commonly utilise 1,4‐dicarbonyl precursors,^28^, ^29^ such compounds are often challenging to access with limited capacity to vary precursor substitution. Hence, multiple methodologies of heterocyclic ring formation have been developed utilising dicarbonyl alternatives, such as allenes,^30^, ^31^, ^32^, ^33^, ^34^, ^35^ alkynyl epoxides,^35^ alkynyl ketones^36^ or 1,4‐alkyne diols.^37^ The methodology introduced by Williams and co‐workers involving the ruthenium/xantphos‐catalysed heterocyclisation of 1,4‐alkyne diols^38^ avoids the isolation of challenging 1,4‐diketone precursors which was particularly attractive in our current system to access furans of type **14**, given the sensitivity of α‐oxygenated carbonyl groups to epimerisation and the compatibility with our readily accessible chiral THP building blocks of type **15** (Scheme 1).^17^, ^18^

**Scheme 2:**
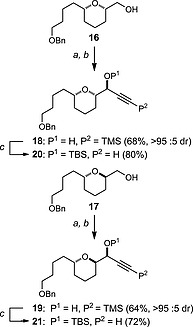
Synthesis of THP‐alkynes **20** and **21**: a) (COCl)_2_, DMSO, CH_2_Cl_2_, –78 °C, 1 h; then Et_3_N –78 °C to r.t., 2 h; b) TMSCCH, Zn(OTf)_2_, (+)‐*N*‐methylephedrine, Et_3_N, PhMe, 70 °C, 16 h; c) (i) K_2_CO_3_, MeOH, r.t. 5 h; (ii) TBSCl, ImH, DMAP, CH_2_Cl_2_, r.t., 16 h. TBSCl = *tert*‐butyldimethylsilyl chloride, ImH = imidazole, DMAP = 4‐dimethylaminopyridine.

Starting from our established THP building block *syn*‐**16** and *anti*‐**17**,^17^, ^19^ Swern oxidation followed by diastereoselective Carreira alkynylation^39^, ^40^, ^41^ gave the corresponding propargyl alcohols **18** and **19**, respectively. While the stereochemical control of the alkynylation reactions was inconsequential for the downstream synthesis, these conditions proved more efficient compared with lithium TMS‐acetylide addition to the respective aldehydes.^42^, ^43^, ^44^, ^45^ The alkynylation products were then treated with potassium carbonate to cleave the *C*‐TMS group and protected as their respective TBS ethers in alkynes **20** and **21** (Scheme 2).

**Scheme 3:**
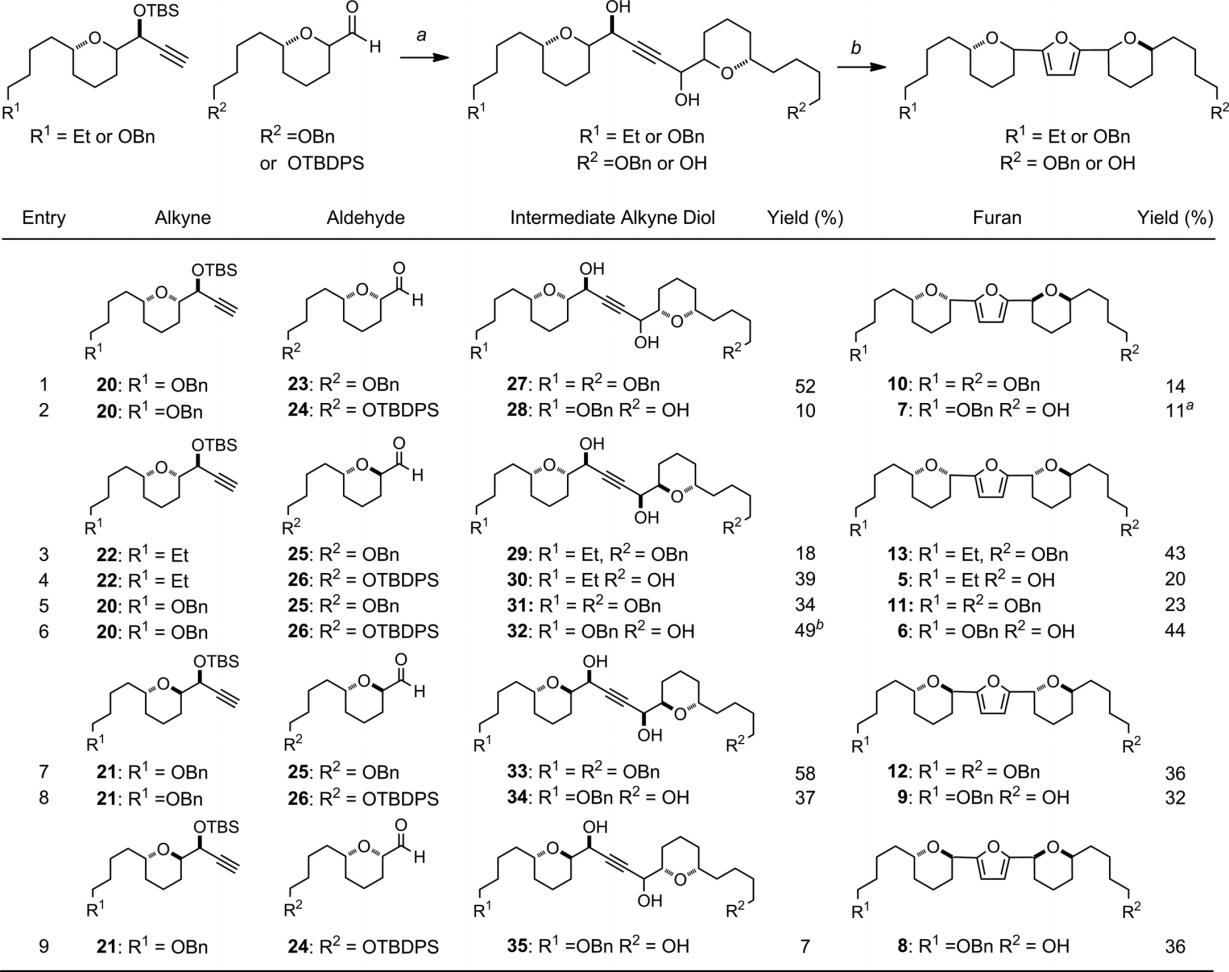
Alkyne coupling and ruthenium/xantphos‐catalysed heterocyclisation to furans: a) (i) alkyne, *n*BuLi, MTBE, –78 °C, 10 min; aldehyde, –78 to r.t., 1 h; (ii) (±)‐CSA, MeOH/CH_2_Cl_2_ (1:1), r.t., 15 h; (b) Ru(PPh_3_)_3_(CO)H_2_ (6 mol‐%), xantphos (6 mol‐%), PhCO_2_H, PhMe, 120 °C, sealed tube, 15 h. ^*a*^Initially co‐isolated with aldehyde by‐product, upon reduction with NaBH_4_ in MeOH furan **7** was isolated in 11 % yield over two steps; ^*b*^ **32** was isolated in 49 % yield after two cycles of (±)‐CSA in MeOH/CH_2_Cl_2_ (1:1), see supporting information. MTBE = methyl *tert*‐butyltether, (±)‐CSA = camphorsulfonic acid, xantphos = 4,5‐bis(diphenylphosphanyl)‐9,9‐dimethylxanthene.

With alkyne precursors **20** and **21** in hand along with the ethyl‐terminated variant **22** (used in our initial synthesis of **1**),^17^ we were in a position to begin the modular assembly of first the key 1,4‐alkyne diol framework followed by the Williams Ru‐catalysed heterocyclisation to access furan derivatives with both diastereomeric and terminal functionality variations, as detailed in Scheme 3. Access to the intermediate 1,4‐alkyne diol precursors was achieved by *n*BuLi deprotonation of the respective alkyne **20**, **21** or **22** followed by addition of the respective THP‐aldehyde **23–26** to give precursors **27–35** (entries 1–9) after silyl group cleavage in modest but workable yields over two steps. In the case of additions to *syn*‐THP aldehydes (entries 1, 2 and 9) the products were formed with low diastereoselectivity at the new carbinol centre which proved inconsequential in the subsequent cyclisation. In contrast the addition of alkynes to *anti*‐THP aldehydes (entries 3–8) proceeded with high levels of Felkin‐Anh selectivity (>95:<5 *d*.r. in all cases). The intermediate alkyne 1,4‐diols where then cyclised under the conditions described by Williams and co‐workers employing the Ru(PPh_3_)_3_(CO)H_2_/xantphos catalyst combination to afford the 2,5‐disubstituted furan derivatives **5–13**, as detailed in Scheme 3. The yields for this isomerisation varied from low to modest, given the complexity of the 1,4‐alkyne diol precursors, in terms of α‐oxygenated tetrahydropyran motifs flanking the central 1,4‐alkyne diol. However, in all cases sufficient material was isolated to facilitate evaluation of antitrypanosomatid activity.

### Biological Evaluation

Synthesised furans were tested in cytotoxicity assays against a panel of kinetoplastid cell lines including bloodstream form (BSF) *T. brucei*, procyclic form (PF) *T. brucei*, epimastigote form *T. cruzi* and promastigote form *L. major*, as well as human HeLa cells for the determination of cell selectivity. EC_50_ values for **5**, the furan analogue of our lead triazole **4**, were similar across all cell lines (Table 2, entries 1 and 2), ranging from 10.8–28.9 µM, and were not dissimilar from **4**. To confirm that **5** was acting on target, we assessed the ability of the compound to inhibit F1 ATP production in digitonin‐permeabilised PF *T. brucei*, which contained permeable cell membranes but intact, functional mitochondria. Like triazole **4**, furan **5** ablated ATP production (Table 2, entry 2), confirming that its trypanocidal activity is through F1 inhibition, supporting docking predictions that both compounds would be similar. These data demonstrated that the central 1,4‐triazole heterocyclic spacer could indeed be substituted for a 2,5‐furan moiety.

**Table 2:** Biological activities of compounds

Entry	Compound	EC_50_ [µM]					SI^** [a]**^	% ATP
		*T. brucei* BSF	*T. brucei* PF	*T.cruzi*	*L.major*	HeLa		inhibition
**1**	Triazole** 4**	1.8 ± 0.1	8.4 ± 0.2	ND	ND	7.0 ± 1.0	4	124 ± 24
**2**	Furan **5**	12.2 ± 0.8	10.8 ± 0.3	28.9 ± 1.4	17.6 ± 0.9	11.3 ± 1.13	1	163 ± 11
**3**	Furan **6**	9.7 ± 0.7	7.2 ± 0.2	24.9 ± 1.1	29.5 ± 1.8	7.9 ± 0.3	1	135 ± 13
**4**	Furan **7**	10.9 ± 0.4	12.2 ± 0.4	30.8 ± 0.7	14.3 ± 0.3	34.4 ± 2.2	3	148 ± 19
**5**	Furan **8**	14.3 ± 0.6	5.0 ± 0.1	18.0 ± 1.0	17.6 ± 0.6	9.1 ± 1.4	1	137 ± 17
**6**	Furan **9**	9.6 ± 1.3	5.1 ± 0.3	21.3 ± 1.4	25.4 ± 1.0	7.9 ± 1.5	1	134 ± 27
**7**	Furan **10**	29.9 ± 3.1	39.0 ± 1.2	>500	>500	>500	>17	–33 ± 21
**8**	Furan **11** a	23.2 ± 1.1	70.3 ± 3.2	>500	>500	>500	>22	–22 ± 31
**9**	Furan **12**	14.9 ± 0.6	32.2 ± 2.1	>500	>500	>500	>34	12 ± 6
**10**	Furan **13**	44.0 ± 3.4	77.3 ± 2.9	127 ± 10	>500	>250	>6	ND

SI (selectivity index) is the ratio of HeLa EC_50_/BSF EC_50_ and measures how selective compounds are towards BSF *T. brucei* relative to human HeLa cell line.

a Furan **11** can also be represented as OBn‐anti‐syn‐OBn.

Likewise, the F1 assay confirmed that OBn‐substituted furans **6**, **7**, **8** and **9** also acted on target, by eliminating F1 ATP production (Table 2, entries 3–6). Cytotoxicity assays revealed that all four compounds had similar potencies across all cell lines tested, ranging from 5.0–34.4 µM, but disappointingly, no compound offered improved toxicity or selectivity over the THP‐Et analogue **5** (Table 2, entry 2), despite the introduction of π‐stacking interactions within the adenine‐binding pocket. It is possible that the enhancement in binding through π‐stacking does not outweigh the potential loss in affinity created through increased inhibitor flexibility, and future studies will investigate the removal of a THP to mitigate this.

Testing of the bis‐THP‐OBn furans **10**, **11** and **12** against F1 revealed that, as expected, they did not inhibit F1 ATP production (Table 2, entries 7–9), and compounds were ineffective against *T. cruzi, L. major* and HeLa cells, failing to significantly inhibit cell growth below 250 µM. This correlation between loss of activity against F1 and against cells by furans **10–12** implies that in these cells, compounds **4–9** induce their cytotoxic activities through F1 inhibition. Given that mammalian and *T. brucei* F1 are highly similar^24^, ^25^ and that **4–9** are relatively non‐specific towards trypanosomatid cells, the design of trypanosomatid‐specific F1 inhibitors of this type may prove challenging.

Remarkably, despite failing to inhibit *T. brucei* F1, **10**, **11** and **12** still displayed activity towards *T. brucei*, albeit with somewhat reduced potencies (Table 2, entries 7–9), providing compounds with selectivities towards BSF *T. brucei* over human HeLa cells of >17, >22 and >34 respectively. This would suggest that these bis‐THP‐OBn compounds have an alternative *T. brucei*‐specific target and that in the design of potential F1‐targetting inhibitors, variants incapable of F1 inhibition have proven fruitful. Future compounds will likely be designed for their inability to inhibit F1 in order to reduce their potent side effects.

Furan **13**, which docks in a flipped orientation providing an *anti‐syn* architecture, like furans **8** and **11**, but with the OH/OBn substituted for an Et group, also failed to significantly inhibit the growth of HeLa, *L. major* and *T. cruzi* (Table 2, entry 10) and both displayed cytotoxicity similar to **11**. This further highlights the importance of hydrogen bonding to the γ‐phosphate‐binding pocket for F1‐binding and inhibition and may therefore form a focal point for avoidance in future investigations.

## Conclusions

Having previously identified the target of our original triazole inhibitor series as mitochondrial F1 and established their hypothetical binding mode, furan analogues were proposed to provide an alternative framework for future analogue derivatisation. We therefore employed a structure‐based approach to design, synthesise and biologically evaluate compounds targeting the FoF1‐ATP synthase. Computational docking indicated that binding of furan analogues was feasible, so the compounds were synthesised by the coupling of pairs of THP building blocks to access 1,4‐alkyne diols, followed by ruthenium/xantphos‐catalysed heterocyclisation. This successful synthetic route is itself an achievement given the complexity of the alkyne diol precursors, which to the best of our knowledge, are the most complex substrates thus described for this method of heterocyclisation. Cytotoxicity assays confirmed that many of the synthesised furans were broadly cytotoxic against a panel of kinetoplastid pathogens as well as human HeLa cells, while an F1 ATP synthase assay confirmed that they acted on target at F1. Docking indicated that substitution of the THP‐Et (**5**) for THP‐OBn (**6–9**) could potentially enhance inhibitor binding through favourable π‐stacking interactions with the receptor, and while all retained F1‐inhibitory properties, enhanced cytotoxicity was not evident. Inhibitors with terminal hydroxylation appeared to form critical hydrogen bonding interactions. Upon substitution with a benzyl ether moiety a loss of activity against most cell lines was observed along with the loss of F1 inhibition, further indicating the on‐target effects of previous analogues. Unexpectedly, these di‐benzoylated compounds **10**, **11** and **12** retained modest activity against *T. brucei*, with selectivities ranging from 17–34 fold, and therefore represent new lead compounds for further development in finding new treatments for HAT.

## Experimental Section

**Compound synthesis and characterisation**

Full details of compound synthesis and spectroscopic characterisation is available in the supporting information.

**Computational Studies**

*Compounds* – bis‐THP furan compounds of *syn‐syn, syn‐anti, anti‐syn* and *anti‐anti* variants and **4** were drawn and converted to three‐dimensional Protein Data Bank (PDB) format structures and minimised using Schrødinger Suite 2012 update 2 (Maestro version 9.3.518) with Macromodel using the MMFF forcefield. PDB coordinates of ADP and ATP were extracted directly from F1. Compounds were then converted to pdbqt format using AutodockTools version 1.5.6.^46^

*Protein receptor sites* – the F1 crystal structures from *T. brucei* (6F5D.pdb)^25^ was used for docking with Autodock Vina.^27^ The receptor was prepared exactly as described in the user manual using Autodock tools.^46^ A grid box of 17 × 15 × 23 Å was centred around the catalytic site comprising chains B (α‐subunit) and F (β‐subunit).

*Compound docking and evaluation* – Ligand‐macromolecule docking was carried out using Autodock Vina. In the same directory files for the ligand and macromolecule, configuration file, and the vina.exe file available for free online were placed. The top 10 binding modes were output and visually assessed with Pymol (Schrødinger). Optimal binding mode and corresponding AutoDock energy (kcal/mol) for each compound were assessed.

**Biological Evaluation**

Compounds were screened against parasites (bloodstream form [BSF] *T. brucei brucei* strain 427, procyclic [PF] form *T. brucei* strain 29–13, epimastigote *T. cruzi* strain CL Brener, promastigote *L. major* strain Friedlin) and human HeLa cells.

*General cell culture* – BSF *T. brucei* were maintained between 10^3^‐10^6^ cells/mL in full HMI‐11 (HMI‐11 medium supplemented with 10 % heat‐inactivated foetal bovine serum (HI‐FBS, Gibco), 1 mM sodium pyruvate, 160 µM thymidine, 50 µM Bathcupromedisulfonic acid, 1 mM hypoxanthine, 1.5 mM L‐cysteine, 60 µM 1‐thioglycerol, and 2.5 mg/L G418 (Calbiochem)) at 37 °C with 5 % v/v CO_2_, as described previously.^47^ PF *T. brucei* were maintained between 10^5^‐10^7^ cells/mL in full SDM‐79 (SDM‐79 medium supplemented with 10 % HI‐FBS, 23.8 mM sodium bicarbonate, 10 µM haemin, 12.5 mg/L G418 and 25 mg/L hygromycin) at 28 °C with 5 % CO_2_.^48^ *T. cruzi* were maintained between 10^6^‐10^7^ cells/mL in full RTH (RPMI 1640 medium supplemented with 10 % HI‐FBS, 16.53 mM HEPES pH 7.2, 4.2 g/L peptone (Fluka), 2 mM sodium glutamate, 100 mg/L streptomycin, 100 KU/L penicillin, 30 µM haemin) at 28 °C with 5 % CO_2_. *L. major* were maintained between 10^5^‐10^7^ cells/mL in full M199 (M199 medium (Sigma) supplemented with 10 % HI‐FBS, 4.17 mM sodium bicarbonate, 40 mM HEPES pH 7.4, 100 µM adenosine and 10 µM haemin) at 28 °C with 5 % CO_2_. HeLa cells were maintained in DMEM supplemented with 10 % HI‐FBS at 37 °C with 5 % CO_2_.

*Cytotoxicity assays* – compounds were serially diluted across 96‐well microtitre plates containing 100 µL of relevant full growth medium in quadruplicate. The final concentration of DMSO (used to solubilise compounds) never exceeded 1 % (v/v). Cells were diluted to 2 × required cell density in relevant full growth medium and 100 µL added to the 100 µL serial dilution (total volume 200 µL per well), generating starting cell densities of 5 × 10^3^/mL (BSF *T. brucei*), 5 × 10^4^/mL (PF *T. brucei*, promastigote* L. major* and HeLa) or 1 × 10^6^/mL (epimastigote *T. cruzi*). Cultures were incubated at the relevant temperature with 5 % CO_2_ for 64 hours, then 10–20 µL of resazurin (1.1 mg/mL in PBS) was added and cultures were incubated for a further 6 h at relevant temperature with 5 % CO_2_ to allow cells to oxidise resazurin to resorufin. Resorufin fluorescence was recorded with a Bio Tek FLX8000 spectrofluorimeter and Gen5 software, using an excitation of 545 ± 15 nm and emission of 590 nm. Curves were calculated using GraFit through the application of four‐parameter least squared non‐linear regression and EC_50_ values were taken as the concentration of drug at the inflection point.

**FoF1 ATP synthase inhibition assay**

Compounds were screened for their abilities to inhibit ATP production from succinate in digitonin‐permeabilised PF *T. brucei* as described previously.^23^ Briefly, PF *T. brucei* were washed in PBS, resuspended in isotonic SoTE buffer (20 mM Tris**·**HCl pH 7.5, 2 mM EDTA pH 7.5, 600 mM sorbitol) and their plasma membranes permeabilised with 0.015 % (w/v) digitonin for 5 min on ice. Permeabilised cells were pelleted at 5000 g for 3 min at 4 °C, resuspended in isotonic assay buffer (20 mM Tris**·**HCl pH 7.4, 15 mM KH_2_PO_4_, 10 mM MgSO_4_, 600 mM sorbitol, 2.5 mg/mL fatty acid‐free BSA), and incubated with/without inhibitor at 200 µM for 10 min at r.t. to allow inhibitors to take effect. Succinate (2.5 mM) was added as substrate and incubated at r.t. for 10 min to allow uptake to the mitochondrion. ATP production was initiated with the addition of 60 µM ADP, and after 30 min incubation at r.t. the reaction was terminated with the addition of 1.37 % (final concentration) perchloric acid, which also released generated ATP from the mitochondrion. After neutralisation with KOH, the degree of ATP production was quantified using the CLSII bioluminescence assay kit (Roche) as directed, recording bioluminescence using Spectramax Gemini XPD spectrofluorimeter (Molecular Devices) and Softmax Pro software. Background luminescence was subtracted from each well and ATP levels calculated relative to uninhibited controls.

## Supporting information

Supporting Information
