# Supplementary material for: Structure‐Based Design, Synthesis and Biological Evaluation of Bis‐Tetrahydropyran Furan Acetogenin Mimics Targeting the Trypanosomatid F1 Component of ATP Synthase
Source: European J Org Chem. 2019 May 29;2019(31-32):5434–40. doi: 10.1002/ejoc.201900541 (PMC6774295; doi:10.1002/ejoc.201900541)
Supplement: Supplementary file 1 — Supporting Information [file EJOC-2019-5434-s001.pdf]

Supporting Information

Structure-Based Design, Synthesis and Biological Evaluation of Bis-Tetrahydropyran Furan Acetogenin Mimics Targeting the Trypanosomatid F1 Component of ATP Synthase

Marija K. Zacharova, Lindsay B. Tulloch, Eoin R. Gould, Andrew L. Fraser, Elizabeth F. King, Stefanie K. Menzies, Terry K. Smith,* and Gordon J. Florence*

ejoc201900541-sup-0001-SupMat.pdf

Contents

General Experimental Conditions S2

Compound Characterization S4

Selected ^{1}H and ^{13}C NMR Spectra S37

General Experimental Conditions

All reactions were performed using oven-dried glassware (oven temp. >100 °C) under a positive pressure of argon or nitrogen with magnetic stirring unless otherwise stated.

^{1}H NMR spectra were recorded using a Bruker Avance III 500 (500 MHz), Bruker Avance 500 (500 MHz), Bruker Avance II 400 (400 MHz), Bruker Avance 400 (400 MHz) or Bruker Avance 300 (300 MHz) and referenced using the quoted deuterated solvent, $CDCl_3$ = 7.26 ppm.[1] Chemical shifts (δ) are reported in ppm (parts per million). Solvent peaks were referenced to literature values. For each resonance, the number of protons, multiplicity pattern, coupling constants (*J*) and interpretation are reported. Coupling constants were reported uncorrected.

^{13}C NMR spectra were recorded using a Bruker Avance III 500 (126 MHz), Bruker Avance 500 (126 MHz), Bruker Avance II 400 (100 MHz), Bruker Avance 400 (100 MHz) or Bruker Avance 300 (75 MHz) and referenced using the quoted deuterated solvent, CDCl3 = 77.16 ppm. Chemical shifts (δ) are reported in ppm (parts per million). Solvent peaks were referenced to literature values.[1]

^{19}F NMR spectra were recorded using a Bruker Avance III 500 (471 MHz), Bruker Avance 500 (471 MHz), Bruker Avance II 400 (377 MHz) or Bruker Avance 400 (377 MHz). Chemical shifts are reported in ppm (parts per million).

Infrared spectra were recorded using a Shimadzu IRAffinity-1 FTIR spectrometer, using attenuated total reflectance (ATR) as the sampling technique. Absorption maxima are recorded in wavenumbers (cm^{-1}).

Optical rotations were recorded using a Perkin Elmer Model 341 polarimeter. A cell of path length, 1 dm was used. Concentration (c) is reported as g/100 mL using spectrophotometric grade $CHCl_3$ unless otherwise stated. Specific rotations were recorded at 20 °C.

HRMS (high resolution mass spectroscopy) was performed using a Thermo Scientific LTQ Orbitrap XL mass spectrometer by the EPSRC national mass spectrometry service (Swansea, UK). Ionisation was carried out using NSI (nano-electrospray ionisation), ESI (electro-spray ionisation) or APCI (Atmospheric pressure chemical ionization). The parent 7ion is quoted.

Analytical thin layer chromatography was carried out on pre-coated Merck silica gel 60 (F_{254}) TLC plates. Visualisation was achieved by UV irradiation or by application of

phosphomolybdic acid (PMA) followed by thermal development. Reagent grade solvents were used as purchased.

Flash column chromatography was performed using Merck silica gel 60 (0.04 – 0.063 mm) under positive pressure. Reagent grade solvents were used as purchased.

Melting points were recorded using a Stuart SMP10 melting point apparatus and are uncorrected.

All reagents/solvents were used as purchased unless otherwise stated. Dichloromethane (CH_2Cl_2), diethyl ether (Et_2O) and tetrahydrofuran (THF) were dried by passing through two columns of alumina (MBRAUN SPS-800 purification system). Distillation/storage of dry solvents was conducted under argon. Oxalyl chloride, triethylamine (Et_3N) and methyl *tert*-butylether (MTBE) were distilled from CaH_2 and stored over CaH_2. Dimethylsulfoxide (DMSO), dimethylformamide (DMF) and pyridine were dried over 4 Å molecular sieves and distilled under vacuum (stored over 4 Å molecular sieves). Methanol (MeOH) was distilled from CaH_2 in a recycling still under argon. Molecular sieves (4 Å) were activated by heating at 200 °C under high vacuum for 6 h, followed by purging with argon. Brine is used to describe a saturated solution of NaCl. Petroleum ether refers to the boiling range: 40 – 60 °C.

Compound Characterization

23: (2*S*,6*S*)-6-[(4-benzyloxy)butyl]-2-(carbaldehyde)tetrahydropyran[1]

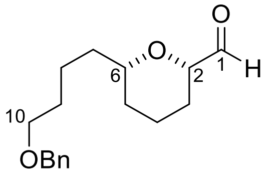

To a solution of dimethyl sulfoxide (334 μL, 4.70 mmol) in CH_2Cl_2 (10 mL) at -78 °C was added oxalyl chloride (302 μL, 3.52 mmol). The reaction mixture was stirred at this temperature for 30 min before the addition of *syn*-THP alcohol **16** (545 mg, 1.96 mmol) as a solution in CH_2Cl_2 (2 mL) *via* cannula. The reaction was stirred for a further 1 h, before the dropwise addition of Et_3N (1.90 mL, 13.7 mmol) and the reaction mixture was warmed to RT over 30 min. After the addition of saturated aqueous NH_4Cl (10 mL), the organics were extracted with CH_2Cl_2 (3 x 20 mL) and the combined organic extracts were dried ($MgSO_4$), filtered and concentrated *in vacuo*. Purification by flash column chromatography (10% Et_2O/Hexane) provided aldehyde **23** (242 mg, 44%) as a colourless oil.

R_f 0.54 (25% EtOAc/Hexane); **^{1}H NMR** (500 MHz, $CDCl_3$) δ 9.59 (1H, s, H^1), 7.36-7.29 (4H, m, ArH), 7.29-7.22 (1H, m, ArH), 4.49 (2H, s, OCH_2Ar), 3.75 (1H, dd, *J* = 11.9, 2.4 Hz, H^2), 3.47 (2H, t, *J* = 6.5 Hz, H^{10}), 3.39-3.30 (1H, m, H^6), 1.95-1.14 (12H, m, CH_2).

Data in agreement with reference[1]

18: (*S*)-1-((2*S*,6*S*)-6-(4-(benzyloxy)butyl)tetrahydropyran-2-yl)-3-(trimethylsilyl)prop-2-yn-1-ol

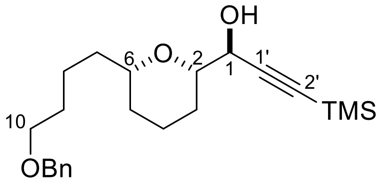

To a mixture of $Zn(OTf)_2$ (51 mg, 0.141 mmol) and (+)-*N*-methylephedrine (29 mg, 0.160 mmol) at RT was added toluene (2 mL) and Et_3N (22 μL, 0.160 mmol). After 2h, TMS acetylene (60 μL, 0.423 mmol) was added and stirred at RT for 20 min. A solution

of aldehyde **23** (26 mg, 94.1 μmol) in toluene (0.5 mL) was added and the solution was heated at 70°C. After 48 h, the reaction was quenched by addition of saturated aqueous NH_4Cl (10 mL), the organics were extracted with CH_2Cl_2 (3 x 20 mL), washed with brine (10 mL), dried ($MgSO_4$), filtered and concentrated *in vacuo*. Purification by flash column chromatography (20% Et_2O/hexane) provided alcohol **18** (24 mg, 68%) as a colourless oil, >95:5 dr by ^{1}H NMR spectroscopy.

R$_f$ 0.39 (30% Et_2O/Hexane); $[\alpha]_D^{20}$ +3.37 (*c* 1.9, $CHCl_3$); **IR** (thin film) 3414 (O-H), 2936 (C-H), 2859 (C-H), 2174 (C≡C), 1454 (aromatic C=C), 1092 (C-O), 1045 (C-O) cm^{-1}; **^{1}H NMR** (300 MHz, $CDCl_3$) δ 7.37 – 7.30 (4H, m, ArH), 7.30 – 7.26 (1H, m, ArH), 4.50 (2H, s, CH_2Ph), 4.18 (1H, dd, *J* = 8.0, 2.5 Hz, H^1), 3.46 (2H, t, *J* = 6.4 Hz, H^{10}), 3.39 – 3.26 (2H, m, H^2+ H^6), 2.89 (1H, d, *J* = 2.6 Hz, OH), 1.93 – 1.73 (2H, m, CH_2), 1.68 – 1.36 (8H, m, CH_2), 1.29 – 1.08 (2H, m, CH_2), 0.17 (9H, s, $Si(CH_3)_3$); **^{13}C NMR** (126 MHz, $CDCl_3$) δ 138.7 (C(quat), ArC), 128.5 (CH, ArC), 127.8 (CH, ArC), 127.7 (CH, ArC), 103.1 (C(quat), $C^{1'}$), 91.0 (C(quat), $C^{2'}$), 80.6 (CH, C^2), 78.1 (CH, C^6), 73.1 (CH_2, CH_2Ph), 70.3 (CH_2, C^{10}), 66.7 (CH, C^1), 36.1 (CH_2), 31.5 (CH_2), 29.8 (CH_2), 27.5 (CH_2), 23.1 (CH_2), 22.3 (CH_2), 0.0 (CH_3, $Si(CH_3)_3$); **HRMS** (NSI$^+$) Calc. for $C_{22}H_{35}O_3Si$ $[M+H]^+$ 375.2350, found 375.2349.

20: ((*(S)*-1-((2*S*,6*S*)-6-(4-(benzyloxy)butyl)tetrahydropyran-2-yl)prop-2-yn-1-yl)oxy)(*tert*-butyl) dimethylsilane

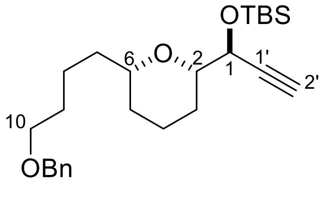

To a solution of alcohol **18** (225 mg, 0.601 mmol) in methanol (50 mL) was added potassium carbonate (1.50 g, 10.9 mmol), after which and the reaction mixture was stirred at RT for 4 h. The reaction was quenched by addition of saturated aqueous NH_4Cl (50 mL). The organics were extracted with EtOAc (3 x 50 mL), dried ($MgSO_4$), filtered and concentrated *in vacuo*. The crude product was dissolved in CH_2Cl_2/MeOH (1:1, 40 mL) and to the solution were added TBSCl (271 mg, 1.80 mmol), imidazole (123 mg, 1.80 mmol) and DMAP (22 mg, 0.180 mmol). The mixture was stirred at RT. After 63 h, TLC analysis showed no conversion, so solvent was removed *in vacuo*, the mixture was re-dissolved in CH_2Cl_2 (30 mL) and re-dosed with TBSCl (367 mg, 2.43 mmol),

imidazole (198 mg, 2.91 mmol) and DMAP (73 mg, 0.598 mmol). The reaction mixture was stirred at RT. After further 28 h, TLC analysis showed no conversion. The reaction was quenched by addition of saturated aqueous NH_4Cl (50 mL). The organics were extracted with CH_2Cl_2 (3 x 20 mL), dried ($MgSO_4$), filtered and concentrated *in vacuo*. ^1H NMR spectroscopic analysis of the crude mixture showed that the reaction was not complete, hence the crude mixture was re-dissolved in CH_2Cl_2 (20 mL) and re-dosed with TBS chloride (1.04 g, 6.90 mmol), imidazole (379 mg, 5.57 mmol) and DMAP (62 mg, 0.507 mmol). After 18 h, the reaction mixture was quenched and the work-up performed as above. Purification by flash column chromatography (10% Et_2O/hexane) provided silyl ether **20** (200 mg, 80%) as a colourless oil.

R_f 0.50 (10% Et_2O/Hexane); $[\alpha]_D^{20}$ -0.50 (*c* 1.2, $CHCl_3$); **IR** (thin film) 2930 (C-H), 2855 (C-H), 1456 (aromatic C=C), 1361 (aromatic C=C), 1098 (C-O) cm^{-1}; **^{1}H NMR** (400 MHz, $CDCl_3$) δ 7.36 – 7.31 (4H, m, Ar<u>H</u>), 7.30 – 7.27 (1H, m, ArH), 4.50 (2H, s, CH_2Ph), 4.30 (1H, dd, *J* = 6.5, 2.2 Hz, H^1), 3.46 (2H, t, *J* = 6.6 Hz, H^{10}), 3.36 – 3.22 (2H, m, H^2+H^6), 2.38 (1H, d, *J* = 2.2 Hz, $H^{2'}$), 1.92 – 1.75 (2H, m, CH_2), 1.68 – 1.10 (10H, m, CH_2), 0.90 (9H, s, $SiC(CH_3)_3$), 0.13 (3H, s, $SiCH_3$), 0.11 (3H, s, $SiCH_3$); **^{13}C NMR** (101 MHz, $CDCl_3$) δ 138.8 (C(quat), ArC), 128.5 (CH, ArC), 127.8 (CH, ArC), 127.6 (CH, ArC), 83.3 (C(quat), $C^{1'}$), 80.6 (CH, C^2), 78.0 (CH, C^6), 73.6 (CH, $C^{2'}$), 73.0 (CH_2, CH_2Ph), 70.6 (CH_2, C^{10}), 66.8 (CH, C^1), 36.3 (CH_2), 31.4 (CH_2), 30.0 (CH_2), 26.6 (CH_2), 25.9 (CH_3, $SiC(CH_3)_3$), 23.3 (CH_2), 22.2 (CH_2), 18.5 (C(quat), $SiC(CH_3)_3$), -4.6 (CH_3, $SiCH_3$), -4.8 (CH_3, $SiCH_3$); **HRMS** (NSI^+) Calc. for $C_{25}H_{41}O_3Si_1$ $[M+H]^+$ 417.2819, found 417.2825.

25: <u>(2*R*,6*S*)-6-[(4-benzyloxy)butyl]-2-(carbaldehyde)tetrahydropyran[1]</u>

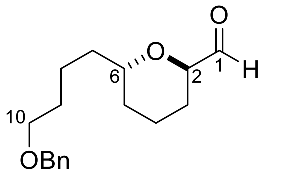

To a solution of dimethyl sulfoxide (153 μL, 2.16 mmol) in CH_2Cl_2 (10 mL) at -78 °C was added oxalyl chloride (139 μL, 1.62 mmol). The reaction mixture was stirred at this temperature for 30 min before the addition of *anti*-THP alcohol **17** (274 mg, 984 μmol) as a solution in CH_2Cl_2 (2 mL) *via* cannula. The reaction was stirred for a further 1 h, before the dropwise addition of Et_3N (870 μL, 6.29 mmol) and the reaction mixture was warmed to RT over 30 min. After the addition of saturated aqueous NH_4Cl (20 mL), the

organics were extracted with CH_2Cl_2 (3 x 20 mL) and the combined organic extracts were dried ($MgSO_4$), filtered and concentrated *in vacuo*. Purification by flash column chromatography (20% Et_2O/Hexane) provided aldehyde **25** (175 mg, 64%) as a colourless oil.

R$_f$ 0.50 (25% EtOAc/Hexane); **^{1}H NMR** (300 MHz, $CDCl_3$) δ 9.83 (1H, s, H^1), 7.38-7.27 (5H, m, ArH), 4.51 (2H, s, OCH_2Ar), 4.17 (1H, dd, *J* = 5.9, 3.1 Hz, H^2), 3.65-3.52 (1H, m, H^6), 3.49 (2H, t, *J* = 6.4 Hz, H^{10}), 2.07-1.14 (12H, m, CH_2).

Data in agreement with reference[1]

19: (*S*)-1-((2*R*,6*S*)-6-(4-(benzyloxy)butyl)tetrahydropyran-2-yl)-3-(trimethylsilyl)prop-2-yn-1-ol

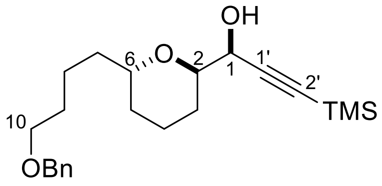

To a mixture of $Zn(OTf)_2$ (2.49 g, 6.84 mmol) and (+)-*N*-methylephedrine (1.39 g, 7.75 mmol) at RT was added toluene (30 mL) and Et_3N (1.07 mL, 7.75 mmol). After 2h, TMS acetylene (2.90 mL, 20.5 mmol) was added and stirred at RT for 20 min. A solution of aldehyde **25** (1.26 g, 4.56 mmol) in toluene (10 mL) was added and the solution was heated at 70°C. After 12 h, the reaction was quenched by addition of saturated aqueous NH_4Cl (50 mL), the organics were extracted with CH_2Cl_2 (3 x 20 mL), dried ($MgSO_4$), filtered and concentrated *in vacuo*. Purification by flash column chromatography (20% Et_2O/hexane) provided epimerised syn-alcohol **18** (308 mg, 18%) as a colourless oil and **19** (1.10 g, 64%) as colourless crystals, **19**:**18** = 78 : 22 dr.

R$_f$ 0.52 (50% Et_2O/Hexane); m.p. 35-38°; $[\alpha]_D^{20}$ -16.6 (*c* 0.5, $CHCl_3$); **IR** (thin film) 3402 (O-H), 2936 (C-H), 2862 (C-H), 2174 (C≡C), 1109 (C-O), cm^{-1}; **^{1}H NMR** (500 MHz, $CDCl_3$) δ 7.37 – 7.30 (4H, m, ArH), 7.30 – 7.27 (1H, m, ArH), 4.50 (2H, s, CH_2Ph), 4.37 (1H, dd, *J* = 6.0, 4.4 Hz, H^1), 3.96 – 3.90 (1H, m, H^6), 3.68 – 3.62 (1H, m, H^2), 3.47 (2H, t, *J* = 6.6 Hz, H^{10}), 2.40 (1H, d, *J* = 6.1 Hz, OH), 1.93 – 1.31 (12H, m, CH_2), 0.17 (9H, s, $Si(CH_3)_3$); **^{13}C**

NMR (126 MHz, $CDCl_3$) δ 138.7 (C(quat)), 128.5 (CH), 127.8 (CH), 127.7 (CH), 103.8 (C(quat)), 91.0 (C(quat)), 73.3 (CH), 73.1 (CH_2), 71.8 (CH), 70.4 (CH_2), 65.4 (CH), 30.9 (CH_2), 29.8 (CH_2), 28.8 (CH_2), 25.3 (CH_2), 22.7 (CH_2), 18.3 (CH_2), 0.0 (CH_3, $Si(CH_3)_3$); **HRMS** (NSI^+) Calc. for $C_{22}H_{34}O_3SiNa$ $[M+Na]^+$ 397.2169, found 397.2167.

21: <u>(((*S*)-1-((2*R*,6*S*)-6-(4-(benzyloxy)butyl)tetrahydropyran-2-yl)prop-2-yn-1-yl)oxy)(*tert*-butyl) dimethylsilane</u>

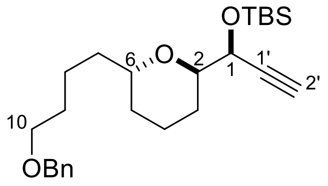

To a solution of alcohol **19** (1.05 g, 2.81 mmol) in methanol (30 mL) was added potassium carbonate (1.94 g, 14.1 mmol) and the reaction mixture was stirred at RT for 30 min. The reaction was quenched by addition of saturated aqueous NH_4Cl (80 mL) and diluted with EtOAc (10 mL) and water (60 mL). The organics were extracted with EtOAc (4 x 20 mL), dried ($MgSO_4$), filtered and concentrated *in vacuo*. The crude product was dissolved in CH_2Cl_2 (30 mL), after which to the solution were added TBSCl (1.90 g, 12.6 mmol) and imidazole (762 mg, 11.2 mmol). The mixture was stirred at RT for 16h. The reaction was quenched by addition of saturated aqueous NH_4Cl (50 mL). The organics were extracted with CH_2Cl_2 (3 x 20 mL), dried ($MgSO_4$), filtered and concentrated *in vacuo*. Purification by flash column chromatography (5→10% Et_2O/hexane) provided silyl ether **21** (841 mg, 72% over two steps) as a colourless oil.

$\mathbf{R_f}$ 0.54 (20% Et_2O/Hexane); $[\alpha]_D^{20}$ +0.111 (*c* 0.9, $CHCl_3$); **IR** (thin film) 2930 (C-H), 2855 (C-H), 1362 (aromatic C=C), 1092 (C-O) cm^{-1}; **^{1}H NMR** (500 MHz, $CDCl_3$) δ 7.35 – 7.32 (4H, m, Ar<u>H</u>), 7.30 – 7.26 (1H, m, ArH), 4.50 (2H, s, CH_2Ph), 4.34 (1H, dd, *J* = 7.5, 2.1 Hz, H^1), 3.83 – 3.73 (1H, m, H^6), 3.67 – 3.59 (2H, m, H^2), 3.47 (2H, t, *J* = 6.6 Hz, H^{10}), 2.35 (1H, d, *J* = 2.1 Hz, $H^{2'}$), 1.81 – 1.19 (12H, m, CH_2), 0.90 (9H, s, $SiC(CH_3)_3$), 0.15 (3H, s, $SiCH_3$), 0.11 (3H, s, $SiCH_3$); **^{13}C NMR** (126 MHz, $CDCl_3$) δ 138.8 (C(quat), ArC), 128.5 (CH, ArC), 127.8 (CH, ArC), 127.6 (CH, ArC), 84.1 (C(quat), $C^{1'}$), 73.7 (CH, C^2), 73.1 (CH, $C^{2'}$), 73.0 (CH_2, CH_2Ph), 72.5 (CH, C^6), 70.1 (CH_2, C^{10}), 64.8 (CH, C^1), 32.4 (CH_2), 29.9 (CH_2), 29.6 (CH_2), 26.3 (CH_2), 25.9 (CH_3, $SiC(CH_3)_3$), 22.4 (CH_2), 18.5 (CH_2), 18.3 (C(quat), $SiC(CH_3)_3$), -4.4 (CH_3, $SiCH_3$), -5.0 (CH_3, $SiCH_3$); **HRMS** (NSI^+) Calc. for $C_{25}H_{44}O_3Si_1N_1$ $[M+NH_4]^+$ 434.3085, found 434.3082.

S36: Carbonyl(dihydro)tris(triphenylphosphine)ruthenium(II) [2]

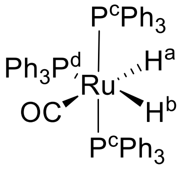

To triphenylphosphine (6.34 g, 23.9 mmol) was added degassed anhydrous methanol (200 mL) and the mixture was heated at reflux for 10 minutes. In quick succession, ruthenium trichloride hydrate (1.00 g) in methanol (40 mL), aqueous formaldehyde (37% w/w) (40 mL) and potassium hydroxide (1.23 g, 21.4 mmol) in methanol (40 mL) were added *via* cannula. The resulting solution was heated for 30 min at reflux and then cooled to 0°C with stirring for further 30 min. The grey precipitate was collected by vacuum filtration and washed with ice-cold absolute ethanol (50 mL), ice-cold water (50 mL), ice-cold absolute ethanol (50 mL) and hexane (50 mL). The crude product was dissolved in toluene and filtered through a column of neutral alumina and washed through thoroughly with toluene. The toluene solution was concentrated in vacuo to 20 mL and anhydrous methanol was added dropwise whilst stirring, producing a precipitate which was collected by vacuum filtration yielding the ruthenium complex **S36** as an off-white solid (1.18 g, minimum yield 27% assuming pure ruthenium chloride).

^{1}H NMR (500 MHz, C_6D_6) δ 7.47 (12H, q, *J* = 5.3 Hz), 7.32 (6H, t, *J* = 8.5 Hz), 7.05 – 6.94 (9H, m), 6.91 (12H, t, *J* = 7.4 Hz), 6.84 (6H, t, *J* = 6.9 Hz), -6.46 (1H, ddt, H^a, J_{Pc-Ha} = 30.8 Hz, J_{Pd-Ha} = 15.2 Hz, J_{Ha-Hb} = 6.5 Hz), -8.29 (1H, ddt, H^b, J_{Pd-Hb} = 74.3 Hz, J_{Pc-Hb} = 28.3 Hz, J_{Ha-Hb} = 6.3Hz); **^{31}P (202 MHz, C_6D_6)** δ 57.5 (d, J_{Pc-Pd} = 17.6 Hz), 45.3 (t, J_{Pd-Pc} = 17.6 Hz).

Data in agreement with reference[2]

S37: <u>(1*R*/*S*,4*S*)-1,4-bis((2*S*,6*S*)-6-(4-(benzyloxy)butyl)tetrahydropyran-2-yl)-4-((*tert*-butyldimethylsilyl)oxy)but-2-yn-1-ol</u>

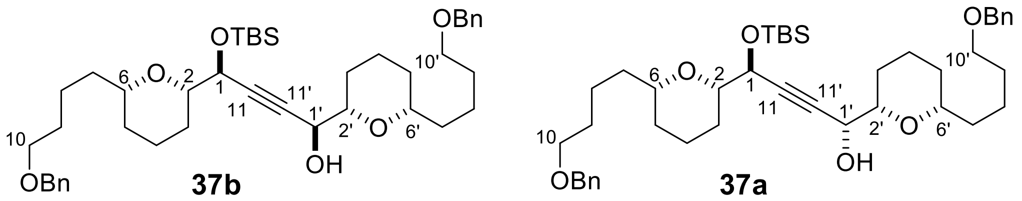

37b **37a**

To a solution of alkyne **20** (592 mg, 1.42 mmol) in MTBE (15 mL) at 0°C was added *n*-BuLi (400 µL, 2.5 M solution in hexane, 1.01 mmol) and the reaction mixture was stirred for 10 min. The temperature was then lowered to -78°C and after further 10 min a solution of aldehyde **23** (93.8 mg, 0.339 mmol) in MTBE (5 mL) was added and the reaction mixture was allowed to warm to RT. The reaction was quenched by addition of saturated aqueous NH_4Cl (20 mL) and CH_2Cl_2 (10 mL). The organics were extracted with CH_2Cl_2 (3 x 20 mL), dried ($MgSO_4$), filtered and concentrated *in vacuo*. Purification by flash column chromatography (10→30% Et_2O/hexane) provided an inseparable mixture of diastereomers of **S37** (130 mg, 55%) as a colourless oil, with 71:29 dr as by ^{1}H NMR spectroscopy.

R$_f$ 0.65 (50% Et_2O/Hexane); **IR** (thin film) 3428 (O-H), 2932 (C-H), 2855 (C-H), 1454 (aromatic C=C), 1362 (aromatic C=C), 1098 (C-O), 1049 (C-O) cm^{-1};

S37a (major diastereomer): **^{1}H NMR** (500 MHz, $CDCl_3$) δ 7.37 – 7.31 (8H, m, ArH), 7.30 – 7.26 (2H, m, ArH), 4.49 (4H, s, CH_2Ph), 4.37 (1H, ddd, *J* = 7.1, 3.3, 1.6 Hz, $H^{1'}$), 4.31 (1H, dd, *J* = 6.7, 1.6 Hz, $H^{1'}$), 3.51 – 3.41 (1H, m, $H^{2'}$), 3.45 (2H, t, *J* = 6.0 Hz, CH_2O), 3.36 – 3.21 (3H, m, H^2 + H^6 + $H^{6'}$), 2.60 (1H, d, *J* = 7.1 Hz, OH), 1.95 - 1.02 (24H, m, CH_2), 0.90 (9H, s, $SiC(CH_3)_3$), 0.13 (3H, s, $SiCH_3$), 0.10 (3H, s, $SiCH_3$); **^{13}C NMR** (126 MHz, $CDCl_3$) δ 138.8 (C(quat), ArC), 138.7 (C(quat), ArC), 128.5 (2 x CH, ArC), 127.8 (2 x CH, ArC), 127.7 (CH, ArC), 127.6 (CH, ArC), 85.0 (C(quat), CHC), 83.3 (C(quat), CHC), 80.9 (CH, C^2), 79.5 (CH, $C^{2'}$), 78.2 (CH, C^6/$C^{6'}$), 77.8 (CH, C^6/$C^{6'}$), 73.0 (CH_2, CH_2Ph), 70.6 (2 x CH_2, C^{10} + $C^{10'}$), 67.1 (CH, C^1), 65.5 (CH, $C^{1'}$), 36.3 (CH_2), 36.2 (CH_2), 31.5 (CH_2), 31.4 (CH_2), 30.0 (CH_2), 29.9 (CH_2), 27.0 (CH_2), 26.0 (CH_2), 25.7 (3 x CH_3, $SiC(CH_3)_3$), 23.4 (CH_2), 23.1 (CH_2), 22.3 (CH_2), 22.2 (CH_2), 18.5 (C(quat), $SiC(CH_3)_3$), -4.5 (CH_3, $SiCH_3$), -4.7 (CH_3, $SiCH_3$);

S37b (minor diastereomer): **^{1}H NMR** (500 MHz, $CDCl_3$) δ 7.37 – 7.31 (8H, m, ArH), 7.30 – 7.26 (2H, m, ArH), 4.50 (4H, s, CH_2Ph), 4.34 – 4.29 (1H, m, $H^{1'}$), 4.23 – 4.18 (1H, m, H^1), 3.46 (2H, t, *J* = 6.5 Hz, CH_2O), 3.34 – 3.28 (2H, m, H^2 + $H^{2'}$), 3.28 – 3.22 (2H, m, H^6 + $H^{6'}$), 2.82 (1H, d, *J* = 2.8 Hz, OH), 1.95 - 1.02 (24H, m, CH_2), 0.89 (9H, s, $SiC(CH_3)_3$), 0.12 (3H, s,

$SiCH_3$), 0.09 (3H, s, $SiCH_3$); **^{13}C NMR** (126 MHz, $CDCl_3$) δ 138.8 (C(quat), ArC), 138.7 (C(quat), ArC), 128.5 (2 x CH, ArC), 127.8 (2 x CH, ArC), 127.7 (CH, ArC), 127.6 (CH, ArC), 85.4 (C(quat), CHC), 83.2 (C(quat), CHC), 80.7 (CH, $C^2/C^{2'}$), 80.6 (CH, $C^2/C^{2'}$), 78.2 (CH, $C^6/C^{6'}$), 77.8 (CH, $C^6/C^{6'}$), 73.0 (CH_2, CH_2Ph), 70.4 (2 x CH_2, C^{10} + $C^{10'}$),67.0 (CH, $C^{1'}$), 66.3 (CH, C^1), 36.3 (CH_2), 36.2 (CH_2), 31.5 (CH_2), 31.4 (CH_2), 30.0 (CH_2), 29.9 (CH_2), 27.0 (CH_2), 26.0 (CH_2), 25.7 (3 x CH_3, $SiC(CH_3)_3$), 23.4 (CH_2), 23.1 (CH_2), 22.3 (CH_2), 22.2 (CH_2), 18.5 (C(quat), $SiC(CH_3)_3$), -4.5 (CH_3, $SiCH_3$), -4.7 (CH_3, $SiCH_3$);

HRMS (NSI^+) Calc. for $C_{42}H_{68}O_6Si_1N_1$ $[M+NH_4]^+$ 710.4810, found 710.4802.

27: (1*R*/*S*,4*S*)-1,4-bis((2*S*,6*S*)-6-(4-(benzyloxy)butyl)tetrahydropyran-2-yl)but-2-yne-1,4-diol

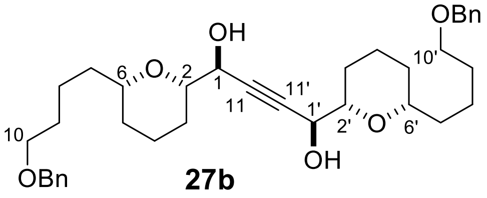

27b

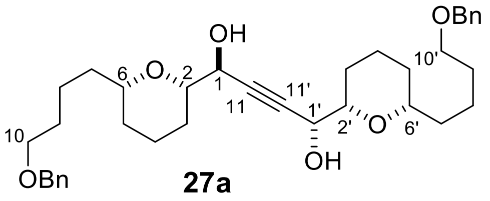

27a

To a solution of alkyne **S37** (120 mg, 173 μmol) in methanol/CH_2Cl_2 (1:1, 10 mL) (±)-CSA (12.2 mg, 52.5 μmol) was added and stirred at RT. After 15 h, the reaction mixture was quenched by the addition of saturated aqueous $NaHCO_3$ (20 mL) and water (5 mL). The organics were extracted with CH_2Cl_2 (3 x 10 mL), dried ($MgSO_4$), filtered and concentrated *in vacuo*. Purification by flash column chromatography (100% Et_2O) provided diol **27** (95.0 mg, 95%) as a colourless oil, with 77:23 dr by ^{1}H NMR spectroscopy.

R_f 0.38 (80% Et_2O/Hexane); $[\alpha]_D^{20}$ -69.6 (*c* 5.6, $CHCl_3$); **IR** (thin film) 3420 (O-H), 2934 (C-H), 2857 (C-H), 1454 (aromatic C=C), 1364 (aromatic C=C), 1092 (C-O), 1047 (C-O) cm^{-1};

27a (major diastereomer): **^{1}H NMR** (500 MHz, $CDCl_3$) δ 7.37 – 7.31 (8H, m, ArH), 7.31 - 7.25 (2H, m, ArH), 4.50 (4H, s, CH_2Ph), 4.41 (1H, dd, *J* = 5.2 Hz, $H^{1'}$), 4.26 – 4.21 (1H, m, H^1), 3.50 - 3.42 (1H, m, $H^{2'}$), 3.46 (4H, t, *J* = 6.5 Hz, H^{10} + $H^{10'}$), 3.39 - 3.27 (3H, m, H^2 + H^6 + $H^{6'}$), 2.93 (1H, d, *J* = 2.5 Hz, OH), 2.68 (1H, d, *J* = 6.3 Hz, OH), 1.93 -1.07 (24H, m, CH_2); **^{13}C NMR** (126 MHz, $CDCl_3$) δ 138.7 (2 x C(quat), ArC), 128.5 (4 x CH, ArC), 127.8 (4 x CH, ArC), 127.6 (2 x CH, ArC), 84.0 (C(quat), CHC), 83.5 (C(quat), CHC), 80.5 (CH, C^2), 79.4 (CH, $C^{2'}$), 78.2 (CH, $C^6/C^{6'}$), 78.1 (CH, $C^6/C^{6'}$), 73.0 (2 x CH_2, CH_2Ph), 70.3 (2 x CH_2, C^{10} +

$C^{10'}$), 66.2 (CH, $C^{1'}$), 65.3 (CH, C^{1}), 36.1 (2 x CH_2), 31.4 (2 x CH_2), 29.9 (CH_2), 29.8 (CH_2), 27.4 (CH_2), 27.3 (CH_2), 25.5 (CH_2), 23.1 (CH_2), 22.3 (CH_2), 22.2 (CH_2);

27b (minor diastereomer): **^{1}H NMR** (500 MHz, $CDCl_3$) δ 7.37 – 7.31 (8H, m, ArH), 7.31 - 7.25 (2H, m, ArH), 4.50 (4H, s, CH_2Ph), 4.26 – 4.21 (2H, m, H^{1} + $H^{1'}$), 3.46 (4H, t, *J* = 6.5 Hz, H^{10} + $H^{10'}$), 3.39 - 3.27 (4H, m, H^{2} + $H^{2'}$ + H^{6} + $H^{6'}$), 2.93 (2H, d, *J* = 2.5 Hz, OH), 1.93 -1.07 (24H, m, CH_2); **^{13}C NMR** (126 MHz, $CDCl_3$) δ 138.7 (2 x C(quat), ArC), 128.5 (4 x CH, ArC), 127.8 (4 x CH, ArC), 127.6 (2 x CH, ArC), 84.0 (C(quat), CHC), 83.5 (C(quat), CHC), 80.4 (2 x CH, C^{2} + $C^{2'}$), 78.2 (2 x CH, C^{6} + $C^{6'}$), 73.0 (2 x CH_2, CH_2Ph), 70.4 (2 x CH_2, C^{10} + $C^{10'}$), 65.3 (2 x CH, C^{1}+ $C^{1'}$), 36.1 (2 x CH_2), 31.4 (2 x CH_2), 29.9 (CH_2), 29.8 (CH_2), 27.4 (CH_2), 27.3 (CH_2), 25.5 (CH_2), 23.1 (CH_2), 22.3 (CH_2), 22.2 (CH_2);

HRMS (NSI^+) Calc. for $C_{36}H_{54}O_6N_1$ $[M+NH_4]^+$ 596.3946, found 596.3935.

10: 2,5-bis((2*S*,6*S*)-6-(4-(benzyloxy)butyl)tetrahydropyran-2-yl)furan

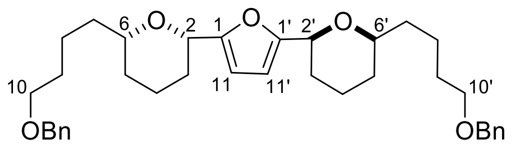

A solution of alkyne diol **27** (48.0 mg, 82.1 μmol), $Ru(PPh_3)_3(CO)H_2$ (4.8 mg, 5.2 μmol), xantphos (2.8 mg, 4.8 μmol) and benzoic acid (5.0 mg, 41 μmol) in toluene (3 mL) was heated in a sealed tube at 120°C. After 15 h, the reaction mixture was quenched by the addition of saturated aqueous $NaHCO_3$ (10 mL). The organics were extracted with CH_2Cl_2 (4 x 10 mL), dried ($MgSO_4$), filtered and concentrated *in vacuo*. Purification by flash column chromatography (10%→100% Et_2O/Hexane) provided furan **10** (6.7 mg, 14%) as a colourless oil.

R_f 0.71 (50% Et_2O); $[\alpha]_D^{20}$ -11.6 (*c* 0.3, $CHCl_3$); **IR** (thin film) 2934 (C-H), 2857 (C-H), 1718 (heteroaromatic C-O), 1454 (aromatic C=C), 1362 (aromatic C=C), 1194 (C-O), 1094 (C-O) , 1042 (C-O), 1029 (C-O) cm^{-1}; **^{1}H NMR** (500 MHz, $CDCl_3$) δ 7.37-7.30 (8H, m, ArH), 7.30 – 7.23 (2H, m, ArH), 6.18 (2H, s, H^{11} + $H^{11'}$), 4.49 (4H, s, CH_2Ph), 4.37 (2H, dd, *J* = 11.3, 2.1 Hz, H^{2} + $H^{2'}$), 3.52 - 3.38 (2H, m, $H^{6'}$ + H^{6}), 3.46 (4H, t, *J* = 6.6 Hz, H^{10} + $H^{10'}$), 1.98 - 1.09 (24H, m, CH_2); **^{13}C NMR** (126 MHz, $CDCl_3$) δ 155.0 (C(quat), $C^{1}/C^{1'}$), 138.8 (C(quat), ArC), 128.5 (CH, ArC), 127.8 (CH, ArC), 127.6 (CH, ArC), 106.7 (CH, $C^{11}/C^{11'}$), 78.4 (CH, $C^{6}/C^{6'}$), 73.4 (CH, C^{2}+ $C^{2'}$), 73.0 (CH_2, CH_2Ph), 70.5 (CH_2, $C^{10}/C^{10'}$), 36.4 (CH_2),

31.2 (CH_2), 29.9 (CH_2), 29.6 (CH_2), 23.8 (CH_2), 22.4 (CH_2); **HRMS** (NSI^+) Calc. for $C_{36}H_{48}O_5Na$ $[M+Na]^+$ 583.3399, found 583.3385.

S38: (1R/*S*,4*S*)-4-((2*S*,6*S*)-6-(4-(benzyloxy)butyl)tetrahydropyran-2-yl)-4-((*tert*-butyldimethylsilyl)oxy)-1-((2*S*,6*S*)-6-(4-((*tert*-butyldiphenylsilyl)oxy)butyl)tetrahydropyran-2-yl)but-2-yn-1-ol

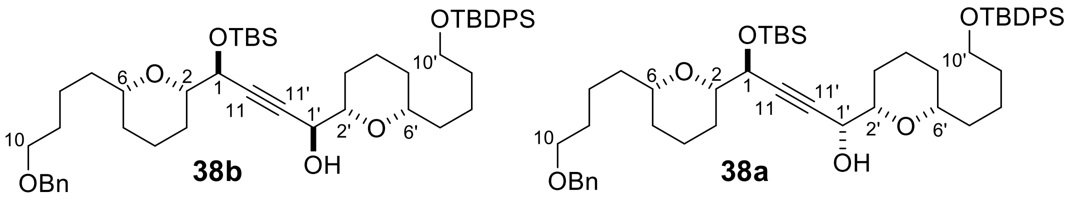

To a solution of alkyne **20** (458 mg, 1.10 mmol) in MTBE (4 mL) at 0°C was added *n*-BuLi (440 μL, 2.5 M solution in hexane, 1.10 mmol) and the reaction mixture was stirred for 5 min. The temperature was then lowered to -78°C and after further 5 min a solution of aldehyde **24**[1] (150 mg, 0.353 mmol) in MTBE (3 mL) was added and the reaction mixture was allowed to warm to RT. The reaction was quenched by addition of saturated aqueous NH_4Cl (20 mL) and CH_2Cl_2 (10 mL). The organics were extracted with CH_2Cl_2 (3 x 20 mL), dried ($MgSO_4$), filtered and concentrated *in vacuo*. Purification by flash column chromatography (5→20% Et_2O/hexane) provided partial separation of major product **S38a** (33 mg, 11%) and a mixed fraction of alcohol diastereomers **S38a/b** (45 mg, 15%), with 67:34 dr by ^1H NMR spectroscopy (overall 78 mg, 26%).

S38a (major diastereomer): $\mathbf{R_f}$ 0.58 (40% Et_2O/Hexane); $[\alpha]_D^{20}$ -6.61 (*c* 0.6, $CHCl_3$); **IR** (thin film) 3439 (O-H), 2928 (C-H), 2855 (C-H), 1462 (aromatic C=C), 1362 (aromatic C=C), 1105 (C-O), cm^{-1}; $\mathbf{^1H}$ **NMR** (700 MHz, $CDCl_3$) δ 7.66 (4H, dd, *J* = 8.0, 1.4 Hz, ArH), 7.29 – 7.31 (11H, m, ArH), 7.31 – 7.26 (1H, m, ArH), 4.49 (4H, s, CH_2Ph), 4.37 (1H, br s, $H^{1'}$), 4.31 (1H, dd, *J* = 6.7, 1.6 Hz, H^1), 3.65 (2H, t, *J* = 6.5 Hz, $H^{10'}$), 3.49 – 3.41 (1H, m, $H^{2'}$), 3.45 (2H, t, *J* = 6.7 Hz, H^{10}), 3.35 – 3.22 (3H, m, $H^2 + H^6 + H^{6'}$), 2.53 (1H, br s, OH), 1.99 - 1.08 (24H, m, CH_2), 1.05 (9H, s, $SiC(CH_3)_3$), 0.89 (9H, s, $SiC(CH_3)_3$), 0.12 (3H, s, $SiCH_3$), 0.10 (3H, s, $SiCH_3$); $\mathbf{^{13}C}$ **NMR** (126 MHz, $CDCl_3$) δ 138.8 (C(quat), ArC), 135.7 (4 x CH, ArC), 134.3 (2 x C(quat), ArC), 129.7 (2 x CH, ArC), 128.5 (2 x CH, ArC), 127.8 (2 x CH, ArC), 127.7 (4 x CH, ArC), 127.6 (CH, ArC), 85.1 (C(quat), CHC), 83.4 (C(quat), CHC), 80.9 (CH, C^2), 79.5 (CH, $C^{2'}$), 78.2 (CH, $C^6/C^{6'}$), 77.8 (CH, $C^6/C^{6'}$), 73.0 (CH_2, CH_2Ph), 70.6 (CH_2, C^{10}), 67.1 (CH, C^1), 65.5 (CH, $C^{1'}$), 64.0 (CH_2, $C^{10'}$), 36.3 (CH_2), 36.2 (CH_2), 32.7 (CH_2), 31.5 (CH_2), 31.4 (CH_2), 30.0 (CH_2), 29.9 (CH_2), 27.0 (3 x CH_3, $SiC(CH_3)_3$), 26.0 (3 x CH_3, $SiC(CH_3)_3$), 25.7 (CH_2), 23.4 (CH_2), 23.2 (CH_2), 22.2 (CH_2), 21.9 (CH_2), 19.4 (C(quat),

$SiC(CH_3)_3$), 18.5 (C(quat), $SiC(CH_3)_3$), -4.5 (CH_3, $SiCH_3$), -4.7 (CH_3, $SiCH_3$); **HRMS** (NSI$^+$) Calc. for $C_{51}H_{76}O_6Si_2Na_1$ [M+Na]$^+$ 863.5073, found 863.5054.

28: (1*S*)-1-((2*S*,6*S*)-6-(4-(benzyloxy)butyl)tetrahydropyran-2-yl)-4-((2*S*,6*S*)-6-(4-hydroxybutyl)tetrahydropyran-2-yl)but-2-yne-1,4-diol

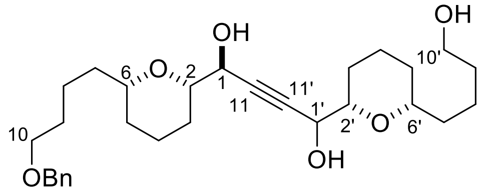

To a solution of alkyne **S38** (33.3 mg, 39.6 μmol) in methanol/$CHCl_3$ (1:1, 6 mL) (±)-CSA (11.0 mg, 47.4 μmol) was added and stirred at RT for 16 h. The reaction mixture was quenched by the addition of saturated aqueous $NaHCO_3$ (10 mL). The organics were extracted with CH_2Cl_2 (3 x 10 mL), dried ($MgSO_4$), filtered and concentrated *in vacuo*. Purification by flash column chromatography (100% Et_2O→100% EtOAc) provided **28** (7.8 mg, 40%) as a colourless oil.

R$_f$ 0.31 (100% Et_2O); **^{1}H NMR** (700 MHz, $CDCl_3$) δ 7.38 – 7.30 (3H, m, ArH), 7.30 – 7.24 (2H, m, ArH), 4.50 (2H, s, CH_2Ph), 4.38 (1H, d, *J* = 3.0 Hz, $H^{1'}$), 4.23 (1H, d, *J* = 7.1 Hz, H^1), 3.46 (2H, t, *J* = 6.4 Hz, $H^{10'}$), 3.49 - 3.41 (1H, m, $H^{2'}$), 3.46 (2H, t, *J* = 6.6 Hz, H^{10}), 3.39 - 3.28 (3H, m, H^2 + H^6 + $H^{6'}$), 3.06 (1H, br s, OH), 2.70 (1H, br s, OH), 1.94 - 1.11 (24H, m, CH_2).

7: 4-((2*S*,6*S*)-6-(5-((2*S*,6*S*)-6-(4-(benzyloxy)butyl)tetrahydropyran-2-yl)furan-2-yl)tetrahydropyran-2-yl)butan-1-ol

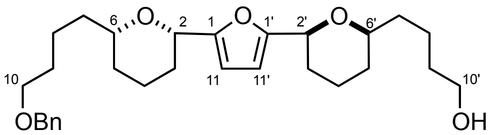

A solution of alkyne triol **28** (7.8 mg, 16.0 μmol), $Ru(PPh_3)_3(CO)H_2$ (1.9 mg, 2.1 μmol), xantphos (2.1 mg, 3.6 μmol) and benzoic acid (6.7 mg, 549 μmol) in toluene (2 mL) was heated in a sealed tube at 130°C. After 7 days, the reaction mixture was quenched by the addition of saturated aqueous $NaHCO_3$ (10 mL). The organics were extracted with CH_2Cl_2 (3 x 10 mL), dried ($MgSO_4$), filtered and concentrated *in vacuo*. Purification by flash column chromatography (10%→100% Et_2O/Hexane) provided a 1:1 mixture of

furan **7** and aldehyde by-product (3.8 mg) as a colourless oil. The mixture was dissolved in methanol (3 mL) and cooled to 0°C. To the mixture was added sodium borohydride (1.9 mg, 50.2 μmol) and the reaction mixture stirred at 0°C for 30 min, then allowed to warm to RT. The reaction was quenched by the addition of saturated aqueous NH_4Cl (10 mL). The organics were extracted with CH_2Cl_2 (3 x 10 mL), dried ($MgSO_4$), filtered and concentrated *in vacuo*. Purification by flash column chromatography (10%→100% Et_2O/Hexane) provided furan **7** (0.8 mg, 11% over 2 steps) as a colourless oil.

R$_f$ 0.31 (70% Et_2O); $[\alpha]_D^{20}$ -11.2 (*c* 0.5, $CHCl_3$); **IR** (thin film) 3426 (O-H), 2934 (C-H), 2857 (C-H), 1743 (heteroaromatic C-O), 1720 (heteroaromatic C-O), 1454 (aromatic C=C), 1364 (aromatic C=C), 1193 (C-O), 1078 (C-O) , 1040 (C-O), 1026 (C-O) cm^{-1}; **^{1}H NMR** (500 MHz, $CDCl_3$) δ 7.37-7.31 (4H, m, ArH), 7.30 – 7.24 (1H, m, ArH), 6.18 (2H, s, H^{11} + $H^{11'}$), 4.49 (4H, s, CH_2Ph), 4.37 (2H, app d, *J* = 10.9 Hz, H^2 + $H^{2'}$), 3.63 (2H, t, *J* = 6.4 Hz, $H^{10'}$), 3.49 – 3.37 (2H, m, H^6 + $H^{6'}$), 3.47 (2H, t, *J* = 6.6 Hz, H^{10}), 2.05 - 1.17 (24H, m, CH_2); **^{13}C NMR** (126 MHz, $CDCl_3$) δ 155.0 (C(quat), C^1/$C^{1'}$), 154.9 (C(quat), C^1/$C^{1'}$), 138.8 (C(quat), ArC),128.5 (2 x CH, ArC), 127.8 (2 x CH, ArC), 127.6 (CH, ArC), 106.7 (2 x CH, C^{11} + $C^{11'}$), 78.4 (2 x CH, C^6+ $C^{6'}$), 73.4 (2 x CH, C^2+ $C^{2'}$), 73.0 (CH_2, CH_2Ph), 70.5 (CH_2, C^{10}), 62.9 (CH_2, $C^{10'}$), 36.3 (CH_2), 36.1 (CH_2), 32.8 (CH_2), 31.3 (CH_2), 31.2 (CH_2), 29.9 (CH_2), 29.6 (CH_2), 29.5 (CH_2), 23.7 (2 x CH_2), 22.3 (CH_2), 21.9 (2 x CH_2); **HRMS** (NSI^+) Calc. for $C_{29}H_{42}O_5Na$ $[M+Na]^+$ 493.2930, found 493.2914.

S39 (1*S*,4*S*)-1-((2*R*,6*S*)-6-(4-(benzyloxy)butyl)tetrahydropyran-2-yl)-4-((*tert*-butyldimethylsilyl)oxy)-4-((2*S*,6*S*)-6-hexyltetrahydropyran-2-yl)but-2-yn-1-ol

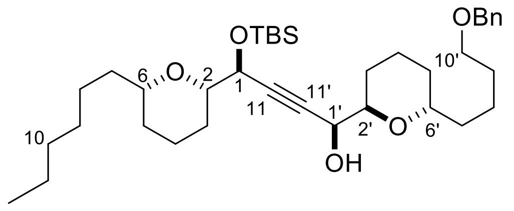

To a solution of alkyne **22**[3] (172 mg, 0.508 mmol) in MTBE (10 mL) at 0°C was added *n*-BuLi (203 μL, 2.5 M solution in hexane, 0.508 mmol). The temperature was then lowered to -78°C and after 10 min a solution of aldehyde **25** (100 mg, 0.362 mmol) in MTBE (5 mL) was added and the reaction mixture was allowed to warm to RT. After 6 h, saturated aqueous NH_4Cl (20 mL) and CH_2Cl_2 (10 mL) were added. The organics were extracted with CH_2Cl_2 (3 x 20 mL), dried ($MgSO_4$), filtered and concentrated *in vacuo*. Purification by flash column chromatography (5→20% Et_2O/hexane) provided alkyne **S39** (39.8 mg, 18%) as a colourless oil, with >95:5 dr by ^{1}H NMR spectroscopy.

R$_f$ 0.64 (50% Et_2O/Hexane); $[\alpha]_D^{20}$ -8.94 (*c* 4.3, $CHCl_3$); **IR** (thin film) 3422 (O-H), 2928 (C-H), 2855 (C-H), 1456 (aromatic C=C), 1362 (aromatic C=C), 1250 (C-O), 1204 (C-O), 1101 (C-O), 1049 (C-O) cm^{-1}; **^{1}H NMR** (500 MHz, $CDCl_3$) δ 7.38 – 7.31 (4H, m, ArH), 7.30 – 7.24 (1H, m, ArH), 4.50 (2H, s, CH_2Ph), 4.40 – 4.35 (1H, m, $H^{1'}$), 4.34 (1H, dd, *J* = 6.7, 1.4 Hz, H^1), 3.97 - 3.88 (1H, m, $H^{6'}$), 3.69 – 3.61 (1H, m, $H^{2'}$), 3.47 (2H, t, *J* = 6.3 Hz, $H^{10'}$), 3.32 (1H, ddd, *J* = 11.0, 6.8, 1.8 Hz, H^2), 3.28 – 3.20 (1H, m, H^6), 2.50 (1H, d, *J* = 6.8 Hz, OH), 1.98 - 1.08 (28H, m, CH_2), 0.91 (9H, s, $SiC(CH_3)_3$), 0.87 (3H, t, *J* = 7.0 Hz, CH_3), 0.13 (3H, s, $SiCH_3$), 0.11 (3H, s, $SiCH_3$); **^{13}C NMR** (126 MHz, $CDCl_3$) δ 138.7 (C(quat), ArC), 128.5 (CH, ArC), 127.8 (CH, ArC), 127.6 (CH, ArC), 85.5 (C(quat), CHC), 83.5 (C(quat), CHC), 80.8 (CH, C^2), 78.0 (CH, C^6), 73.2 (CH, $C^{6'}$), 73.1 (CH_2, CH_2Ph), 71.9 (CH, $C^{2'}$), 70.4 (CH_2, $C^{10'}$), 67.0 (CH, C^1), 65.2 (CH, $C^{1'}$), 36.6 (CH_2), 32.0 (CH_2), 31.5 (CH_2), 30.7 (CH_2), 29.8 (CH_2), 29.6 (CH_2), 28.6 (CH_2), 27.0 (CH_2), 25.9 (CH_3, $SiC(CH_3)_3$), 25.6 (CH_2), 25.5 (CH_2), 23.4 (CH_2), 22.8 (CH_2), 22.7 (CH_2), 18.5 (C(quat), $SiC(CH_3)_3$),), 18.1 (CH_2), 14.3 (CH_3), -4.5 (CH_3, $SiCH_3$), -4.7 (CH_3, $SiCH_3$); **HRMS** (NSI^+) Calc. for $C_{37}H_{66}O_5Si_1N_1$ $[M+NH_4]^+$ 632.4705, found 632.4695.

29: (1*S*,4*S*)-1-((2*R*,6*S*)-6-(4-(benzyloxy)butyl)tetrahydropyran-2-yl)-4-((2*S*,6*S*)-6-hexyltetrahydropyran-2-yl)but-2-yne-1,4-diol

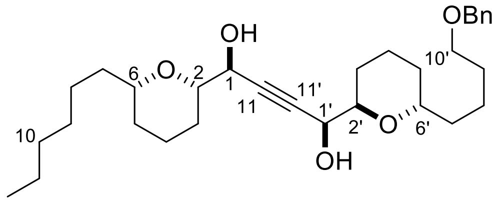

To a solution of alkyne **S39** (39.8 mg, 64.7 μmol) in methanol/CH_2Cl_2 (1:1, 10 mL) (±)-CSA (10 mg, 43.1 μmol) was added and the reaction mixture was stirred at RT. After 14 h, the reaction mixture was quenched by the addition of saturated aqueous $NaHCO_3$ (20 mL) and water (5 mL). The organics were extracted with CH_2Cl_2 (4 x 20 mL), dried ($MgSO_4$), filtered and concentrated *in vacuo*. Purification by flash column chromatography (50%→100% Et_2O/Hexane) provided diol **29** in quantitative yield (32.6 mg) as a colourless oil.

R$_f$ 0.44 (70% Et_2O/Hexane); $[\alpha]_D^{20}$ -110 (*c* 3.3, $CHCl_3$); **IR** (thin film) 3399 (O-H), 2930 (C-H), 2857 (C-H), 1454 (aromatic C=C), 1364 (aromatic C=C), 1204 (C-O), 1094 (C-O), 1042 (C-O), cm^{-1}; **^{1}H NMR** (500 MHz, $CDCl_3$) δ 7.37 - 7.31 (4H, m, ArH), 7.30 - 7.24 (1H, m, ArH), 4.50 (2H, s, CH_2Ph), 4.44 - 4.37 (1H, m, $H^{1'}$), 4.24 (1H, d, *J* = 7.7 Hz, H^1), 3.91

(1H, dd, *J* = 8.4, 3.5 Hz, H$^{6'}$), 3.70 - 3.62 (1H, m, H$^{2'}$), 3.47 (2H, t, *J* = 6.5 Hz, H$^{10'}$), 3.36 (1H, ddd, *J* = 11.0, 7.8, 1.8 Hz, H^{2}), 3.33 - 3.25 (1H, m, H^{6}), 2.98 – 2.90 (1H, m, OH), 2.53 (1H, d, *J* = 6.1 Hz, OH), 1.93 - 1.06 (28H, m, CH_2), 0.87 (3H, t, *J* = 6.9 Hz, CH_3); **^{13}C NMR** (126 MHz, $CDCl_3$) δ 138.7 (C(quat), ArC), 128.5 (CH, ArC), 127.8 (CH, ArC), 127.6 (CH, ArC), 84.3 (C(quat), CHC), 83.9 (C(quat), CHC), 80.5 (CH, C^{2}), 78.3 (CH, C^{6}), 73.2 (CH, C$^{6'}$), 73.0 (CH_2, CH_2Ph), 71.9 (CH, C$^{2'}$), 70.4 (CH_2, C$^{10'}$), 66.2 (CH, C^{1}), 65.0 (CH, C$^{1'}$), 36.4 (CH_2), 31.9 (CH_2), 31.4 (CH_2), 30.8 (CH_2), 29.8 (CH_2), 29.5 (CH_2), 28.7 (CH_2), 27.5 (CH_2), 25.6 (CH_2), 25.5 (CH_2), 23.1 (CH_2), 22.8 (CH_2), 22.7 (CH_2), 18.1 (CH_2), 14.3 (CH_3); **HRMS** (NSI^+) Calc. for $C_{31}H_{52}O_5N_1$ $[M+NH_4]^+$ 518.3840, found 518.3831.

13: (2*S*,6*R*)-2-(4-(benzyloxy)butyl)-6-(5-((2*S*,6*S*)-6-hexyltetrahydropyran-2-yl)furan-2-yl)tetrahydropyran

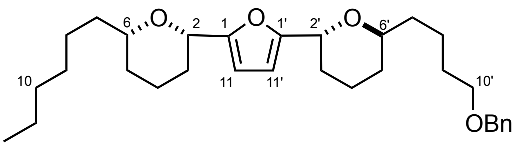

A solution of alkyne diol **29** (15.2 mg, 30.4 μmol), $Ru(PPh_3)_3(CO)H_2$ (6.1 mg, 6.7 μmol), xantphos (9.4 mg, 16 μmol) and benzoic acid (6.1 mg, 50 μmol) in toluene (3 mL) was heated in a sealed tube at 130°C. After 39 h, the reaction mixture was quenched by the addition of saturated aqueous $NaHCO_3$ (10 mL). The organics were extracted with CH_2Cl_2 (4 x 10 mL), dried ($MgSO_4$), filtered and concentrated *in vacuo*. Purification by flash column chromatography (5→100% Et_2O/Hexane) provided furan **13** (6.5 mg, 43%) as a colourless oil.

R_f 0.37 (20% Et_2O/Hexane); $[\alpha]_D^{20}$ -30.8 (*c* 0.7, $CHCl_3$); **IR** (thin film) 2932 (C-H), 2857 (C-H), 1456 (aromatic C=C), 1362 (aromatic C=C), 1098 (C-O), 1042 (C-O), cm^{-1}; **^{1}H NMR** (500 MHz, $CDCl_3$) δ 7.36 - 7.31 (4H, m, ArH), 7.30 – 7.22 (1H, m, ArH), 6.20 (1H, d, *J* = 3.1 Hz, H^{11}/H$^{11'}$), 6.16 (1H, dd, *J* = 3.2, 0.8 Hz, H^{11}/H$^{11'}$), 4.95 - 4.85 (1H, m, H$^{2'}$), 4.49 (2H, s, CH_2Ph), 4.37 (1H, dd, *J* = 11.3, 1.9 Hz, H^{2}), 3.57 - 3.49 (1H, m, H$^{6'}$),3.46 (4H, t, *J* = 6.6 Hz, H^{10}+ H$^{10'}$), 3.43 - 3.36 (1H, m, H^{6}), 2.05 - 1.12 (24H, m, CH_2), 0.87 (3H, t, *J* = 6.9 Hz, CH_3); **^{13}C NMR** (126 MHz, $CDCl_3$) δ 155.1 (C(quat), C^{1}/C$^{1'}$), 154.1 (C(quat), C^{1}/C$^{1'}$), 138.8 (C(quat), ArC), 128.5 (CH, ArC), 127.8 (CH, ArC), 127.6 (CH, ArC), 108.1 (CH, C^{11}/C$^{11'}$), 106.6 (CH, C^{11}/C$^{11'}$), 78.6 (CH, C^{6}), 73.5 (CH, C^{2}), 73.0 (CH_2, CH_2Ph), 71.6 (CH, C$^{6'}$), 70.6 (CH_2, OCH_2), 68.7 (CH, C$^{2'}$), 36.6 (CH_2), 35.1 (CH_2), 32.0 (CH_2), 31.3 (CH_2), 30.9 (CH_2), 29.9

(CH$_2$), 29.7 (CH$_2$), 29.5 (CH$_2$), 27.3 (CH$_2$), 25.7(CH$_2$), 23.8 (CH$_2$), 22.3 (CH$_2$), 19.7 (CH$_2$), 14.3 (CH$_3$); **HRMS** (NSI$^+$) Calc. for $C_{31}H_{46}O_4Na_1$ [M+Na]$^+$ 505.3288, found 505.3278.

S40: (1*S*,4*S*)-4-((*tert*-butyldimethylsilyl)oxy)-1-((2*R*,6*S*)-6-(4-((*tert*-butyldiphenylsilyl)oxy)butyl) tetrahydropyran-2-yl)-4-((2*S*,6*S*)-6-hexyltetrahydropyran-2-yl)but-2-yn-1-ol

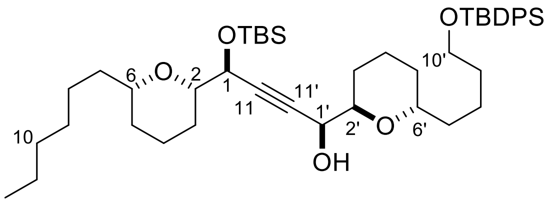

To a solution of alkyne **22**[3] (119 mg, 0.351 mmol) in MTBE (10 mL) at 0°C was added *n*-BuLi (0.22 mL, 1.6 M solution in hexane, 0.351 mmol) and the reaction mixture was stirred for 10 min. The temperature was then lowered to -78°C and after further 3 min a solution of aldehyde **26**[1] (141 mg, 0.332 mmol) in MTBE (5 mL) was added and the reaction mixture was allowed to warm to RT. The reaction was quenched by addition of saturated aqueous NH$_4$Cl (20 mL) and CH$_2$Cl$_2$ (10 mL). The organics were extracted with CH$_2$Cl$_2$ (3 x 20 mL), dried (MgSO$_4$), filtered and concentrated *in vacuo*. Purification by flash column chromatography (10→20% Et$_2$O/hexane) provided alkyne **S40** (119 mg, 47%) as a colourless oil, with >95:5 dr by ^{1}H NMR spectroscopy.

R$_f$ 0.48 (30% Et$_2$O/Hexane); $[\alpha]_D^{20}$ -5.47 (*c* 8.8, CHCl$_3$); **IR** (thin film) 3428 (O-H), 2928 (C-H), 2857 (C-H), 1462 (aromatic C=C), 1427 (aromatic C=C), 1250 (C-O), 1103 (C-O), 1049 (C-O) cm^{-1}; **^{1}H NMR** (500 MHz, CDCl$_3$) δ 7.72 – 7.64 (4H, m, ArH), 7.46 – 7.36 (6H, m, ArH), 4.43 – 4.31 (2H, m, H$^{1'}$+H^1), 3.96 - 3.88 (1H, m, H$^{6'}$), 3.71 – 3.63 (1H, m, H$^{2'}$), 3.68 (2H, t, *J* = 6.4 Hz, H$^{10'}$), 3.34 (1H, ddd, *J* = 11.2, 6.7, 1.8 Hz, H^2), 3.29 – 3.22 (1H, m, H^6), 2.51 (1H, d, *J* = 6.7 Hz, OH), 2.00 - 1.12 (28H, m, CH$_2$), 1.06 (9H, s, SiC(CH$_3$)$_3$), 0.92 (9H, s, SiC(CH$_3$)$_3$), 0.89 (3H, t, *J* = 6.9 Hz, CH$_3$), 0.15 (3H, s, SiCH$_3$), 0.13 (3H, s, SiCH$_3$); **^{13}C NMR** (126 MHz, CDCl$_3$) δ 135.7 (CH, ArC), 134.2 (C(quat), ArC), 129.6 (CH, ArC), 127.7 (CH, ArC), 85.4 (C(quat), CHC), 83.5 (C(quat), CHC), 80.8 (CH, C^2), 78.0 (CH, C^6), 73.2 (CH, C$^{6'}$), 71.9 (CH, C$^{2'}$), 67.0 (CH, C^1), 65.2 (CH, C$^{1'}$), 63.9 (CH$_2$, C$^{10'}$), 36.6 (CH$_2$), 32.6 (CH$_2$), 32.0 (CH$_2$), 31.5 (CH$_2$), 30.6 (CH$_2$), 29.6 (CH$_2$), 28.6 (CH$_2$), 27.0 (CH$_3$, SiC(CH$_3$)$_3$ + CH$_2$), 25.9 (CH$_3$, SiC(CH$_3$)$_3$), 25.6 (CH$_2$), 25.5 (CH$_2$), 23.4 (CH$_2$), 22.8 (CH$_2$), 22.3 (CH$_2$), 19.3 (C(quat), SiC(CH$_3$)$_3$), 18.5 (C(quat), SiC(CH$_3$)$_3$), 18.1 (CH$_2$), 14.3 (CH$_3$), -4.5 (CH$_3$, SiCH$_3$), -4.7 (CH$_3$, SiCH$_3$); **HRMS** (NSI$^+$) Calc. for $C_{46}H_{78}O_5Si_2N_1$ [M+NH$_4$]$^+$ 780.5413, found 780.5407.

30: (1*S*,4*S*)-1-((2*S*,6*S*)-6-hexyltetrahydropyran-2-yl)-4-((2*R*,6*S*)-6-(4-hydroxybutyl)tetrahydropyran-2-yl)but-2-yne-1,4-diol

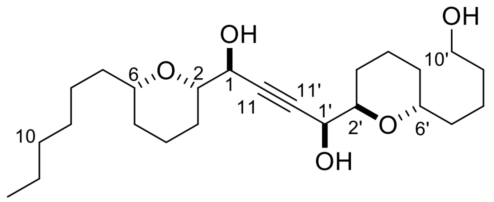

To a solution of alkyne **S40** (92.0 mg, 0.121 mmol) in methanol/CH_2Cl_2 (1:1, 10 mL) was added (±)-CSA (22.0 mg, 96.4 μmol). The reaction mixture was stirred at RT for 72 h, after which it was quenched by the addition of saturated aqueous $NaHCO_3$ (20 mL). The organics were extracted with CH_2Cl_2 (3 x 20 mL), dried ($MgSO_4$), filtered and concentrated *in vacuo*. Purification by flash column chromatography (70% Et_2O/Pet.Ether→100% Et_2O→100% EtOAc) provided triol **30** (40.8 mg, 82%) as a white solid, m.p. 63-64°C.

R$_f$ 0.68 (100% EtOAc); $[\alpha]_D^{20}$ -181.7 (*c* 0.5, $CHCl_3$); **IR** (thin film) 3385 (O-H), 2928 (C-H), 2857 (C-H), 1456 (aromatic C=C), 1263 (C-O), 1202 (C-O), 1086 (C-O), 1038 (C-O) cm^{-1}; **^{1}H NMR** (500 MHz, $CDCl_3$) δ 4.46 – 4.35 (1H, m, H$^{1'}$), 4.25 (1H, d, *J* = 7.4 Hz, H^1), 3.90 (1H, dd, *J* = 9.3, 4.3 Hz, H$^{6'}$), 3.70 - 3.60 (1H, m, H$^{2'}$), 3.65 (2H, t, *J* = 6.2 Hz, H$^{10'}$), 3.40 - 3.33 (1H, m, H^2), 3.33 - 3.26 (1H, m, H^6), 3.16 (1H, br s, OH), 2.61 (1H, d, *J* = 6.1 Hz, OH), 2.11 - 1.10 (28H, m, CH_2), 0.87 (3H, t, *J* = 6.8 Hz, CH_3); **^{13}C NMR** (126 MHz, $CDCl_3$) δ 84.6 (C(quat), CHC), 83.8 (C(quat), CHC), 80.4 (CH, C^2), 78.4 (CH, C^6), 73.0 (CH, C$^{6'}$), 72.3 (CH, C$^{2'}$), 66.2 (CH, C^1), 64.7 (CH, C$^{1'}$), 62.9 (CH_2, C$^{10'}$), 36.5 (CH_2), 32.6 (CH_2), 32.0 (CH_2), 31.4 (CH_2), 31.1 (CH_2), 29.5 (CH_2), 29.1 (CH_2), 27.4 (CH_2), 25.7 (CH_2), 25.6 (CH_2), 23.1 (CH_2), 22.8 (CH_2), 22.2 (CH_2), 18.2 (CH_2), 14.3 (CH_3); **HRMS** (ASAP) Calc. for $C_{24}H_{43}O_5$ $[M+H]^+$ 411.3110, found 411.3108.

5: <u>4-((2*S*,6*R*)-6-(5-((2*S*,6*S*)-6-hexyltetrahydropyran-2-yl)furan-2-yl)tetrahydropyran-2-yl)butan-1-ol</u>

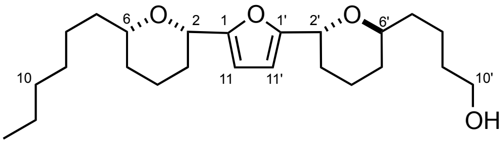

A solution of alkyne triol **30** (40.0 mg, 97.4 μmol), $Ru(PPh_3)_3(CO)H_2$ (2.2 mg, 2.4 μmol), xantphos (2.9 mg, 5.0 μmol) and benzoic acid (1.6 mg, 13 μmol) in toluene (3 mL) was heated in a sealed tube at 130°C. After 58 h, the reaction mixture was quenched by the addition of saturated aqueous $NaHCO_3$ (10 mL). The organics were extracted with CH_2Cl_2 (4 x 10 mL), dried ($MgSO_4$), filtered and concentrated *in vacuo*. Purification by flash column chromatography (20%→40% Et_2O/Hexane) provided a separable mixture of aldehyde by-product **S41** (2.8 mg, 7.4%) and furan **5** (7.7 mg, 20%) as colourless oils.

R_f 0.50 (70% Et_2O/Pet. Ether); $[\alpha]_D^{20}$ -40.8 (*c* 0.8, $CHCl_3$); **IR** (thin film) 3428 (O-H), 2932 (C-H), 2857 (C-H), 1456 (aromatic C=C), 1196 (C-O), 1082 (C-O) , 1042 (C-O), 1016 (C-O) cm^{-1}; **^1H NMR** (500 MHz, $CDCl_3$) δ 6.21 (1H, d, *J* = 3.1 Hz, $H^{11}/H^{11'}$), 6.17 (1H, dd, *J* = 3.2, 0.9 Hz, $H^{11}/H^{11'}$), 4.97 – 4.88 (1H, m, $H^{2'}$), 4.38 (1H, dd, *J* = 11.3, 2.0 Hz, H^2), 3.62 (2H, t, *J* = 6.5 Hz, $H^{10'}$), 3.56 - 3.48 (1H, m, $H^{6'}$), 3.45 - 3.38 (1H, m, H^6), 2.08 - 1.13 (28H, m, CH_2), 0.87 (3H, t, *J* = 6.9 Hz, CH_3); **^{13}C NMR** (126 MHz, $CDCl_3$) δ 155.2 (C(quat), $C^1/C^{1'}$), 154.0 (C(quat), $C^1/C^{1'}$), 108.2 (CH, $C^{11}/C^{11'}$), 106.6 (CH, $C^{11}/C^{11'}$), 78.6 (CH, C^6), 73.5 (CH, C^2), 71.4 (CH, $C^{6'}$), 68.7 (CH, $C^{2'}$), 63.0 (CH_2, $C^{10'}$), 36.6 (CH_2), 35.0 (CH_2), 32.8 (CH_2), 32.0 (CH_2), 31.2 (CH_2), 31.0 (CH_2), 29.7 (CH_2), 29.5 (CH_2), 27.2 (CH_2), 25.7 (CH_2), 23.8 (CH_2), 22.8 (CH_2), 21.8 (CH_2), 19.7 (CH_2), 14.3 (CH_3); **HRMS** (NSI^+) Calc. for $C_{24}H_{40}O_4Na$ $[M+Na]^+$ 415.2819, found 415.2806.

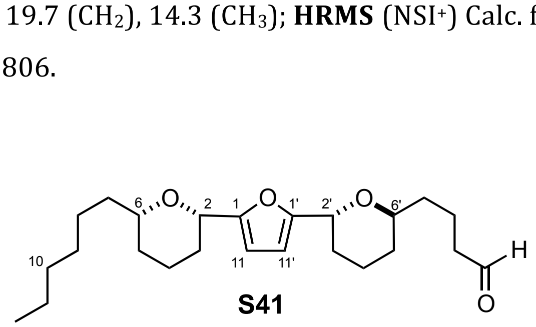

S41

S41: R_f 0.61 (50% Et_2O/Pet.Ether); $[\alpha]_D^{20}$ -40.0 (*c* 0.3, $CHCl_3$); **IR** (thin film) 2930 (C-H), 2857 (C-H), 1726 (C=O), 1456 (aromatic C=C), 1084 (C-O), 1040 (C-O), 1016 (C-O), cm^{-1}; **^1H NMR** (500 MHz, $CDCl_3$) δ 9.75 (1H, t, *J* = 1.8 Hz, CHO), 6.21 (1H, d, *J* = 3.1 Hz, $H^{11}/H^{11'}$), 6.17 (1H, dd, *J* = 3.1, 0.9 Hz, $H^{11}/H^{11'}$), 4.98 – 4.85 (1H, m, $H^{2'}$), 4.38 (1H, dd, *J* = 11.3, 1.9 Hz, H^2), 3.58 - 3.45 (1H, m, $H^{6'}$), 3.45 - 3.37 (1H, m, H^6), 2.44 (2H, t, *J* = 7.3 Hz, $H^{9'}$), 2.07 -

1.08 (26H, m, CH_2), 0.87 (3H, t, *J* = 6.9 Hz, CH_3); **^{13}C NMR** (126 MHz, $CDCl_3$) δ 203.0 (CH, CHO), 155.3 (C(quat), $C^1/C^{1'}$), 153.9 (C(quat), $C^1/C^{1'}$), 108.3 (CH, $C^{11}/C^{11'}$), 106.6 (CH, $C^{11}/C^{11'}$), 78.6 (CH, C^6), 73.5 (CH, C^2), 71.1 (CH, $C^{6'}$), 68.7 (CH, $C^{2'}$), 43.8 (CH_2, $C^{9'}$), 36.6 (CH_2), 34.6 (CH_2), 32.0 (CH_2), 31.3 (CH_2), 31.1 (CH_2), 29.7 (CH_2), 29.5 (CH_2), 27.1 (CH_2), 25.7 (CH_2), 23.8 (CH_2), 22.8 (CH_2), 19.7 (CH_2), 18.5 (CH_2), 14.3 (CH_3); **HRMS** (NSI$^+$) Calc. for $C_{24}H_{38}O_4Na$ $[M+Na]^+$ 413.2662, found 413.2649.

S42 <u>(1*S*,4*S*)-1-((2*R*,6*S*)-6-(4-(benzyloxy)butyl)tetrahydropyran-2-yl)-4-((2*S*,6*S*)-6-(4-(benzyloxy)butyl) tetrahydropyran-2-yl)-4-((tert-butyldimethylsilyl)oxy)but-2-yn-1-ol</u>

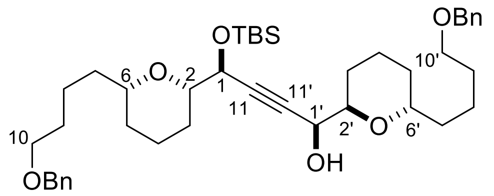

To a solution of alkyne **20** (130 mg, 0.311 mmol) in MTBE (2 mL) at 0°C was added *n*-BuLi (194 μL, 1.6 M solution in hexane, 0.311 mmol). The temperature was then lowered to -78°C and after 10 min a solution of aldehyde **25** (165 mg, 0.593 mmol) in MTBE (2.5 mL) was added. After 1 h, the reaction mixture was allowed to warm to RT. After further 17 h, saturated aqueous NH_4Cl (5 mL) and CH_2Cl_2 (5 mL) were added. The organics were extracted with CH_2Cl_2 (3 x 10 mL), washed with brine (5 mL), dried ($MgSO_4$), filtered and concentrated *in vacuo*. Purification by flash column chromatography (10→50% Et_2O/hexane) provided alkyne **S42** (117 mg, 54%) as a colourless oil, with >95:5 dr by ^{1}H NMR spectroscopy.

R$_f$ 0.31 (40% Et_2O/Hexane); $[\alpha]_D^{20}$ -8.55 (*c* 1.6, $CHCl_3$); **IR** (thin film) 3420 (O-H), 2934 (C-H), 2855 (C-H), 1456 (aromatic C=C), 1362 (aromatic C=C), 1098 (C-O) cm^{-1}; **^{1}H NMR** (500 MHz, $CDCl_3$) δ 7.36 – 7.31 (8H, m, ArH), 7.31 – 7.26 (2H, m, ArH), 4.51 (2H, s, CH_2Ph), 4.50 (2H, s, CH_2Ph), 4.40 – 4.35 (1H, m, $H^{1'}$), 4.34 (1H, dd, *J* = 6.6, 1.2 Hz, H^1), 3.96-3.89 (1H, m, $H^{6'}$), 3.66 (1H, dd, *J* = 8.9, 3.7 Hz, $H^{2'}$), 3.47 (4H, m, H^{10}+ $H^{10'}$), 3.36 – 3.29 (1H, m, H^2), 3.26 (1H, dd, *J* = 7.0, 3.9 Hz, H^6), 2.59 (1H, d, *J* = 6.8 Hz, OH), 1.92-1.08 (24H, m, CH_2), 0.90 (9H, s, $SiC(CH_3)_3$), 0.13 (3H, s, $SiCH_3$), 0.11 (3H, s, $SiCH_3$); **^{13}C NMR** (126 MHz, $CDCl_3$) δ 138.7 (C(quat), ArC), 138.6 (C(quat), ArC), 128.5 (CH, ArC), 128.4 (CH, ArC), 127.7 (2 x CH, ArC), 127.6 (CH, ArC), 127.5 (CH, ArC), 85.3 (C(quat), CHC),

83.6 (C(quat), CHC), 80.8 (CH, C^2), 77.8 (CH, C^6), 73.1 (CH, $C^{6'}$), 73.0 (CH_2, CH_2Ph), 72.9 (CH_2, CH_2Ph), 71.9 (CH, $C^{2'}$), 70.5 (CH_2, OCH_2), 70.4 (CH_2, OCH_2), 66.9 (CH, C^1), 65.1 (CH, $C^{1'}$), 36.3 (CH_2), 31.4 (CH_2), 30.7 (CH_2), 29.9 (CH_2), 29.8 (CH_2), 28.6 (CH_2), 26.9 (CH_2), 25.9 (CH_3, $SiC(CH_3)_3$), 25.5 (CH_2), 23.3 (CH_2), 22.7 (CH_2), 22.2 (CH_2), 18.4 (CH_2), 18.1 (C(quat), $SiC(CH_3)_3$), -4.5 (CH_3, $SiCH_3$), -4.7 (CH_3, $SiCH_3$); **HRMS** (NSI^+) Calc. for $C_{42}H_{68}O_6Si_1N_1$ $[M+NH_4]^+$ 710.4810, found 710.4807.

31: (1*S*,4*S*)-1-((2*R*,6*S*)-6-(4-(benzyloxy)butyl)tetrahydropyran-2-yl)-4-((2*S*,6*S*)-6-(4-benzyloxy)butyl) tetrahydropyran-2-yl)but-2-yne-1,4-diol

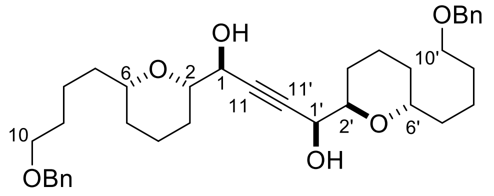

To a solution of alkyne **S42** (76.6 mg, 111 µmol) in methanol/CH_2Cl_2 (1:1, 2.5 mL) (±)-CSA (6.1 mg, 26.3 µmol) was added as a solution in methanol (0.5 mL) and stirred at RT. After 20 h, the reaction mixture was quenched by the addition of saturated aqueous $NaHCO_3$ (10 mL). The organics were extracted with CH_2Cl_2 (3 x 10 mL), washed with brine (10 mL), dried ($MgSO_4$), filtered and concentrated *in vacuo*. Purification by flash column chromatography (80%→100% Et_2O/Hexane) provided diol **31** (40.1 mg, 62%) as a colourless oil.

R$_f$ 0.38 (80% Et_2O/Hexane); $[\alpha]_D^{20}$ -8.21 (*c* 0.3, $CHCl_3$); **IR** (thin film) 3422 (O-H), 2936 (C-H), 2857 (C-H), 1456 (aromatic C=C), 1098 (C-O), 1042 (C-O), cm^{-1}; **^{1}H NMR** (500 MHz, $CDCl_3$) δ 7.38-7.31 (8H, m, ArH), 7.31-7.26 (2H, m, ArH), 4.50 (2H, s, CH_2Ph), 4.49 (2H, s, CH_2Ph), 4.44-4.36 (1H, m, $H^{1'}$), 4.23 (1H, ddd, *J* = 7.6, 2.9, 1.6 Hz, H^1), 3.96-3.88 (1H, m, $H^{6'}$), 3.70-3.61 (1H, m, $H^{2'}$), 3.53 – 3.40 (4H, m, H^{10}+ $H^{10'}$), 3.39-3.27 (2H, m, H^2 + H^6), 2.90 (1H, d, *J* = 3.0 Hz, OH), 2.47 (1H, d, *J* = 6.5 Hz, OH), 1.89-1.07 (24H, m, CH_2); **^{13}C NMR** (126 MHz, $CDCl_3$) δ 138.7 (2 x C(quat), ArC), 128.5 (2 x CH, ArC), 127.8 (2 x CH, ArC), 127.7 (2 x CH, ArC), 84.3 (C(quat), CHC), 83.9 (C(quat), CHC), 80.5 (CH, C^2), 78.2 (CH, C^6), 73.2 (CH, $C^{6'}$), 73.1 (2 x CH_2, CH_2Ph), 71.8 (CH, $C^{2'}$), 70.5 (CH_2, OCH_2), 70.4 (CH_2, OCH_2), 66.2 (CH, C^1), 65.0 (CH, $C^{1'}$), 36.1 (CH_2), 31.4 (CH_2), 30.8 (CH_2), 29.9 (CH_2), 29.8 (CH_2), 28.8 (CH_2), 27.4 (CH_2), 25.5 (CH_2), 23.1 (CH_2), 22.8 (CH_2), 22.4 (CH_2), 18.1 (CH_2); **HRMS** (NSI^+) Calc. for $C_{36}H_{54}O_6N_1$ $[M+NH_4]^+$ 596.3946, found 596.3939.

11: 2-((2*R*,6*S*)-6-(4-(benzyloxy)butyl)tetrahydropyran-2-yl)-5-((2*S*,6*S*)-6-(4-(benzyloxy)butyl) tetrahydropyran-2-yl)furan

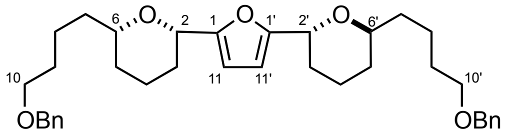

A solution of alkyne diol **31** (39.1 mg, 67.6 μmol), $Ru(PPh_3)_3(CO)H_2$ (0.6 mg, 0.68 μmol), xantphos (1.0 mg, 1.7 μmol) and benzoic acid (2.0 mg, 16.4 μmol) in toluene (1.5 mL) was heated under reflux. After 24 h, TLC analysis showed very poor conversion, so more $Ru(PPh_3)_3(CO)H_2$ (1.2 mg, 1.4 μmol) was added and the solution heated under reflux for further 21 h. The solvent was removed under reduced pressure. Purification by flash column chromatography (20% Et_2O/Hexane) provided furan **11** (8.6 mg, 23%) as a colourless oil.

R_f 0.61 (40% Et_2O/Hexane); $[\alpha]_D^{20}$ -26.6 (*c* 0.9, $CHCl_3$); **IR** (thin film) 2936 (C-H), 2859 (C-H), 1717 (heteroaromatic C-O), 1456 (aromatic C=C), 1099 (C-O), 1042 (C-O), cm^{-1}; **^{1}H NMR** (500 MHz, $CDCl_3$) δ 7.37-7.30 (8H, m, ArH), 7.30 – 7.26 (2H, m, ArH), 6.20 (1H, d, *J* = 3.1 Hz, $H^{11}/H^{11'}$), 6.16 (1H, d, *J* = 3.1 Hz, $H^{11}/H^{11'}$), 4.91 (1H, t, *J* = 4.3 Hz, $H^{2'}$), 4.49 (4H, s, CH_2Ph), 4.37 (1H, dd, *J* = 11.3, 1.9 Hz, H^2), 3.57-3.50 (1H, m, $H^{6'}$),3.46 (4H, t, *J* = 6.6 Hz, H^{10}+ $H^{10'}$), 3.44-3.37 (1H, m, H^6), 2.04-1.15 (24H, m, CH_2); **^{13}C NMR** (126 MHz, $CDCl_3$) δ 155.1 (C(quat), $C^1/C^{1'}$), 154.1 (C(quat), $C^1/C^{1'}$), 138.8 (2 x C(quat), ArC),128.5 (2 x CH, ArC), 127.8 (2 x CH, ArC), 127.6 (2 x CH, ArC), 108.0 (CH, $C^{11}/C^{11'}$), 106.6 (CH, $C^{11}/C^{11'}$), 78.4 (CH, C^6), 73.5 (CH, C^2), 73.0 (2 x CH_2, CH_2Ph), 71.6 (CH, $C^{6'}$), 70.6 (CH_2, OCH_2), 70.5 (CH_2, OCH_2), 68.7 (CH, $C^{2'}$), 36.3 (CH_2), 35.1 (CH_2), 31.2 (CH_2), 30.9 (CH_2), 29.9 (CH_2), 29.8 (CH_2), 29.7 (CH_2), 27.3 (CH_2), 23.7 (CH_2), 22.3 (2 x CH_2), 19.7 (CH_2); **HRMS** (NSI^+) Calc. for $C_{36}H_{48}O_5Na_1$ $[M+Na]^+$ 583.3399, found 583.3379.

S43: (1*S*,4*S*)-4-((2*S*,6*S*)-6-(4-(benzyloxy)butyl)tetrahydropyran-2-yl)-4-((*tert*-butyldimethylsilyl)oxy)-1-((2*R*,6*S*)-6-(4-((*tert*-butyldiphenylsilyl)oxy)butyl)tetrahydropyran-2-yl)but-2-yn-1-ol

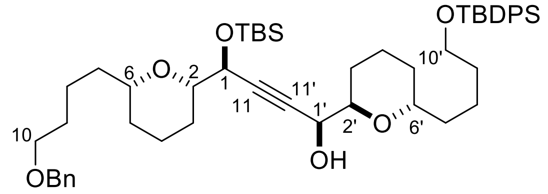

To a solution of alkyne **20** (592 mg, 1.42 mmol) in MTBE (15 mL) at 0°C was added *n*-BuLi (0.57 mL, 2.5 M solution in hexane, 1.42 mmol) and the reaction mixture was stirred for 10 min. The temperature was then lowered to -78°C and after further 10 min a solution of aldehyde **26**[1] (113 mg, 0.264 mmol) in MTBE (5 mL) was added and the reaction mixture was allowed to warm to RT. The reaction was quenched by addition of saturated aqueous NH_4Cl (20 mL) and CH_2Cl_2 (10 mL). The organics were extracted with CH_2Cl_2 (3 x 20 mL), dried ($MgSO_4$), filtered and concentrated *in vacuo*. Purification by flash column chromatography (5→20% Et_2O/hexane) provided alkyne **S43** (161 mg, 73%) as a colourless oil, with >95:5 dr by ^1H NMR spectroscopy.

R$_f$ 0.53 (40% Et_2O/Hexane); $[\alpha]_D^{20}$ -6.96 (*c* 1.0, $CHCl_3$); **IR** (thin film) 3441 (O-H), 2932 (C-H), 2857 (C-H), 1472 (aromatic C=C), 1427 (aromatic C=C), 1362 (aromatic C=C), 1105 (C-O) cm^{-1}; **^{1}H NMR** (500 MHz, $CDCl_3$) δ 7.69 – 7.63 (4H, m, ArH), 7.45 – 7.30 (10H, m, ArH), 7.30 – 7.27 (1H, m, ArH), 4.49 (2H, s, CH_2Ph), 4.38 – 4.34 (1H, m, $H^{1'}$), 4.33 (1H, dd, *J* = 6.6, 1.3 Hz, H^1), 3.94-3.86 (1H, m, $H^{6'}$), 3.66 (2H, t, *J* = 6.4 Hz, $H^{10'}$), 3.69 – 3.61 (1H, m, $H^{2'}$), 3.45 (2H, t, *J* = 6.7 Hz, H^{10}), 3.31 (1H, ddd, *J* = 10.9, 6.5, 1.6 Hz, H^2), 3.28 – 3.22 (1H, m, H^6), 2.50 (1H, d, *J* = 6.6 Hz, OH), 1.90 - 1.09 (24H, m, CH_2), 1.04 (9H, s, $SiC(CH_3)_3$), 0.89 (9H, s, $SiC(CH_3)_3$), 0.12 (3H, s, $SiCH_3$), 0.10 (3H, s, $SiCH_3$); **^{13}C NMR** (126 MHz, $CDCl_3$) δ 138.8 (C(quat), ArC), 135.7 (CH, ArC), 134.2 (C(quat), ArC), 129.7 (CH, ArC), 128.5 (CH, ArC), 127.8 (CH, ArC), 127.7 (CH, ArC), 127.6 (CH, ArC), 85.4 (C(quat), CHC), 83.6 (C(quat), CHC), 80.8 (CH, C^2), 77.8 (CH, C^6), 73.3 (CH, $C^{6'}$), 73.0 (CH_2, CH_2Ph), 71.9 (CH, $C^{2'}$), 70.6 (CH_2, C^{10}), 67.0 (CH, C^1), 65.2 (CH, $C^{1'}$), 63.9 (CH_2, $C^{10'}$), 36.3 (CH_2), 32.6 (CH_2), 31.5 (CH_2), 30.6 (CH_2), 30.0 (CH_2), 28.5 (CH_3, $SiC(CH_3)_3$), 27.0 (CH_2), 26.9 (CH_2), 26.0 (CH_3, $SiC(CH_3)_3$), 25.5 (CH_2), 23.4 (CH_2), 22.4 (CH_2), 22.2 (CH_2), 19.4 (C(quat), $SiC(CH_3)_3$), 18.5 (C(quat), $SiC(CH_3)_3$), 18.1 (CH_2), -4.5 (CH_3, $SiCH_3$), -4.7 (CH_3, $SiCH_3$); **HRMS** (NSI$^+$) Calc. for $C_{51}H_{80}O_6Si_2N_1$ $[M+NH_4]^+$ 858.5519, found 858.5516.

32: (1*S*,4*S*)-1-((2*S*,6*S*)-6-(4-(benzyloxy)butyl)tetrahydropyran-2-yl)-4-((2*R*,6*S*)-6-(4-hydroxybutyl)tetrahydropyran-2-yl)but-2-yne-1,4-diol

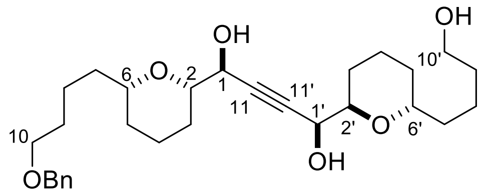

To a solution of alkyne **S43** (149 mg, 177 μmol) in methanol/CH_2Cl_2 (1:1, 20 mL) (±)-CSA (8.9 mg, 38.3 μmol) was added and stirred at RT. After 16 h, the reaction mixture was quenched by the addition of saturated aqueous $NaHCO_3$ (20 mL) and water (10 mL). The organics were extracted with CH_2Cl_2 (3 x 10 mL), washed with brine (10 mL), dried ($MgSO_4$), filtered and concentrated *in vacuo*. Purification by flash column chromatography (70%→100% Et_2O/Hexane) provided a separable mixture of partially deprotected diol **S44** (56.0 mg, 44%) and triol **32** (31.5 mg, 36%) as yellow oils. Mixed fractions of **S44** and **32** (52.3 mg, 7 : 1 **S44:32**) were dissolved in methanol/CH_2Cl_2 (1:1, 8 mL) and (±)-CSA (8.3 mg, 35.7 μmol).added at RT The reaction mixture was stirred for 21 h, after which it was quenched by the addition of saturated aqueous $NaHCO_3$ (10 mL) and water (10 mL). The organics were extracted with CH_2Cl_2 (3 x 10 mL), washed with brine (10 mL), dried ($MgSO_4$), filtered and concentrated *in vacuo*. Purification by flash column chromatography (100% Et_2O →100% EtOAc) provided further triol **32** (26.3 mg, 85%) as a yellow oil. After two deprotection cycles a combined 57.8 mg of **32** was isolated (67%)

32: R$_f$ 0.31 (100% Et_2O); $[\alpha]_D^{20}$ -17.5 (*c* 3.3, $CHCl_3$); **IR** (thin film) 3393 (O-H), 2936 (C-H), 2860 (C-H), 1456 (aromatic C=C), 1362 (aromatic C=C), 1090 (C-O), 1042 (C-O) cm^{-1}; **^{1}H NMR** (500 MHz, $CDCl_3$) δ 7.36 - 7.30 (4H, m, ArH), 7.30-7.26 (1H, m, ArH), 4.49 (2H, s, CH_2Ph), 4.38 (1H, br s, $H^{1'}$), 4.25 (1H, d, *J* = 7.1 Hz, H^1), 3.91 - 3.83 (1H, m, $H^{6'}$), 3.69 - 3.59 (1H, m, $H^{2'}$), 3.63 (2H, t, *J* = 6.0 Hz, $H^{10'}$), 3.45 (2H, t, *J* = 6.4 Hz, H^{10}), 3.43 - 3.28 (2H, m, H^2 + H^6), 3.05 (1H, br s, OH), 2.47 (1H, d, *J* = 6.5 Hz, OH), 1.91 - 1.26 (24H, m, CH_2); **^{13}C NMR** (126 MHz, $CDCl_3$) δ 138.6 (C(quat), ArC), 128.5 (CH, ArC), 127.8 (CH, ArC), 127.7 (CH, ArC), 84.8 (C(quat), CHC), 83.8 (C(quat), CHC), 80.4 (CH, C^2), 78.2 (CH, C^6), 73.0 (CH_2, CH_2Ph), 72.9 (CH, $C^{6'}$), 72.5 (CH, $C^{2'}$), 70.4 (CH_2, C^{10}), 66.0 (CH, C^1), 64.4 (CH, $C^{1'}$), 62.7 (CH_2, $C^{10'}$), 36.1 (CH_2), 32.5 (CH_2), 31.4 (CH_2), 31.2 (CH_2), 29.8 (CH_2), 29.2 (CH_2), 27.3 (CH_2), 25.8 (CH_2), 23.1 (CH_2), 22.4 (CH_2), 22.1 (CH_2), 18.2 (CH_2); **HRMS** (NSI$^+$) Calc. for $C_{29}H_{48}O_6N_1$ $[M+NH_4]^+$ 506.3476, found 506.3466.

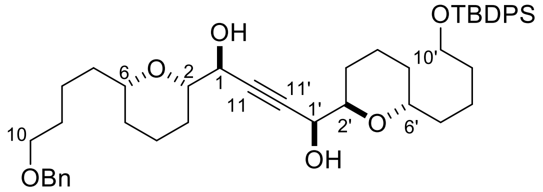

S44: $\mathbf{R_f}$ 0.29 (70% Et_2O/Hexane); $[\alpha]_D^{20}$ -8.68 (*c* 0.7, $CHCl_3$); **IR** (thin film) 3393 (O-H), 2934 (C-H), 2859 (C-H), 1456 (aromatic C=C), 1429 (aromatic C=C), 1362 (aromatic C=C), 1096 (C-O), 1045 (C-O) cm^{-1}; **^{1}H NMR** (500 MHz, $CDCl_3$) δ 7.66 (4H, dd, *J* = 7.9, 1.4 Hz, ArH), 7.45 -7.27 (11H, m, ArH), 4.50 (2H, s, CH_2Ph), 4.42 - 4.36 (1H, m, $H^{1'}$), 4.26 – 4.18 (1H, m, H^1), 3.93 - 3.84 (1H, m, $H^{6'}$), 3.66 (2H, t, *J* = 6.4 Hz, $H^{10'}$), 3.71 - 3.61 (1H, m, $H^{2'}$), 3.46 (2H, t, *J* = 6.5 Hz, H^{10}), 3.39 - 3.26 (2H, m, H^2 + H^6), 2.86 (1H, d, *J* = 2.8 Hz, OH), 2.47 (1H, d, *J* = 6.4 Hz, OH), 1.93 -1.10 (24H, m, CH_2), 1.04 (9H, s, $SiC(CH_3)_3$); **^{13}C NMR** (126 MHz, $CDCl_3$) δ 138.7 (C(quat), ArC), 135.7 (CH, ArC), 134.2 (C(quat), ArC), 129.7 (CH, ArC), 128.5 (CH, ArC), 127.8 (CH, ArC), 127.7 (2 x CH, ArC), 84.2 (C(quat), CHC), 83.9 (C(quat), CHC), 80.5 (CH, C^2), 78.2 (CH, C^6), 73.3 (CH, $C^{6'}$), 73.1 (CH_2, CH_2Ph), 71.7 (CH, $C^{2'}$), 70.4 (CH_2, C^{10}), 66.2 (CH, C^1), 65.1 (CH, $C^{1'}$), 63.9 (CH_2, $C^{10'}$), 36.1 (CH_2), 32.6 (CH_2), 31.4 (CH_2), 30.6 (CH_2), 29.9 (CH_2), 28.7 (CH_2), 27.5 (CH_2), 27.0 (3 x CH_3, $SiC(CH_3)_3$), 25.5 (CH_2), 23.1 (CH_2), 22.4 (CH_2), 22.3 (CH_2), 19.4 ((quat), $SiC(CH_3)_3$), 18.1 (CH_2); **HRMS** (NSI^+) Calc. for $C_{45}H_{66}O_6Si_1N_1$ $[M+NH_4]^+$ 744.4654, found 744.4653.

6: 4-((2*S*,6*R*)-6-(5-((2*S*,6*S*)-6-(4-(benzyloxy)butyl)tetrahydropyran-2-yl)furan-2-yl)tetrahydropyran-2-yl)butan-1-ol

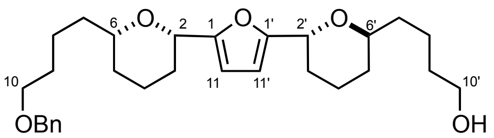

A solution of alkyne triol **32** (35.2 mg, 72.0 µmol), $Ru(PPh_3)_3(CO)H_2$ (3.3 mg, 3.6 µmol), xantphos (2.1 mg, 3.6 µmol) and benzoic acid (0.4 mg, 3.6 µmol) in toluene (3 mL) was heated in a sealed tube at 130°C. After 43 h, the reaction mixture was quenched by the addition of saturated aqueous $NaHCO_3$ (10 mL). The organics were extracted with CH_2Cl_2 (4 x 10 mL), dried ($MgSO_4$), filtered and concentrated *in vacuo*. Purification by flash column chromatography (20%→50% Et_2O/Hexane) provided alcohol **6** (14.8 mg, 44%) and aldehyde by-product **S45** (6.0 mg, 18%) as colourless oils.

R$_f$ 0.70 (100% Et_2O); $[\alpha]_D^{20}$ -37.4 (*c* 0.8, $CHCl_3$); **IR** (thin film) 3649 (O-H), 2936 (C-H), 2860 (C-H), 1717 (heteroaromatic C-O), 1653 (heteroaromatic C-O), 1456 (aromatic C=C), 1092 (C-O), 1045 (C-O) cm^{-1}; **^{1}H NMR** (500 MHz, $CDCl_3$) δ 7.37-7.30 (4H, m, ArH), 7.30 – 7.26 (1H, m, ArH), 6.21 (1H, d, *J* = 3.1 Hz, $H^{11}/H^{11'}$), 6.17 (1H, dd, *J* = 3.2, 0.8 Hz, $H^{11}/H^{11'}$), 4.45 – 4.90 (1H, m, $H^{2'}$), 4.49 (4H, s, CH_2Ph), 4.38 (1H, dd, *J* = 11.3, 1.9 Hz, H^2), 3.61 (2H, t, *J* = 6.2 Hz, $H^{10'}$), 3.55 - 3.38 (2H, m, $H^{6'}$ + H^6), 3.46 (2H, t, *J* = 6.6 Hz, H^{10}), 2.06-1.11 (24H, m, CH_2); **^{13}C NMR** (126 MHz, $CDCl_3$) δ 155.1 (C(quat), $C^1/C^{1'}$), 154.0 (C(quat), $C^1/C^{1'}$), 138.8 (C(quat), ArC),128.5 (CH, ArC), 127.8 (CH, ArC), 127.6 (CH, ArC), 108.2 (CH, $C^{11}/C^{11'}$), 106.6 (CH, $C^{11}/C^{11'}$), 78.5 (CH, C^6), 73.5 (CH, C^2), 73.0 (CH_2, CH_2Ph), 71.4 (CH, $C^{6'}$), 70.5 (CH_2, C^{10}), 68.7 (CH, $C^{2'}$), 63.0 (CH_2, $C^{10'}$), 36.3 (CH_2), 35.0 (CH_2), 32.8 (CH_2), 31.2 (CH_2), 31.1 (CH_2), 29.9 (CH_2), 29.6 (CH_2), 27.1 (CH_2), 23.7 (CH_2), 22.3 (CH_2), 21.8 (CH_2), 19.7 (CH_2); **HRMS** (NSI$^+$) Calc. for $C_{29}H_{42}O_5Na$ $[M+Na]^+$ 493.2930, found 493.2913.

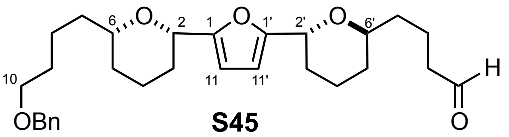

S45

S45: R$_f$ 0.45 (50% Et_2O/Hexane); $[\alpha]_D^{20}$ -38.4 (*c* 0.4, $CHCl_3$); **IR** (thin film) 2934 (C-H), 2859 (C-H), 1724 (C=O), 1454 (aromatic C=C), 1362 (aromatic C=C), 1096 (C-O), 1042 (C-O), cm^{-1}; **^{1}H NMR** (500 MHz, $CDCl_3$) δ 9.74 (1H, s, CHO), 7.36-7.30 (4H, m, ArH), 7.30 – 7.26 (1H, m, ArH), 6.21 (1H, d, *J* = 3.2 Hz, $H^{11}/H^{11'}$), 6.17 (1H, dd, *J* = 3.2, 0.9 Hz, $H^{11}/H^{11'}$), 4.95 – 4.89 (1H, m, $H^{2'}$), 4.49 (4H, s, CH_2Ph), 4.37 (1H, dd, *J* = 11.3, 2.0 Hz, H^2), 3.54 - 3.38 (2H, m, $H^{6'}$+ H^6),3.46 (2H, t, *J* = 6.6 Hz, H^{10}), 2.43 (2H, t, *J* = 7.1 Hz, $H^{9'}$), 2.04-1.16 (22H, m, CH_2); **^{13}C NMR** (126 MHz, $CDCl_3$) δ 203.0 (CH, CHO), 155.2 (C(quat), $C^1/C^{1'}$), 153.9 (C(quat), $C^1/C^{1'}$), 138.8 (C(quat), ArC), 128.5 (CH, ArC), 127.8 (CH, ArC), 127.6 (CH, ArC), 108.3 (CH, $C^{11}/C^{11'}$), 106.6 (CH, $C^{11}/C^{11'}$), 78.4 (CH, C^6), 73.5 (CH, C^2), 73.0 (CH_2, CH_2Ph), 71.1 (CH, $C^{6'}$), 70.5 (CH_2, C^{10}), 68.7 (CH, $C^{2'}$), 43.8 (CH_2, $C^{9'}$), 36.4 (CH_2), 34.6 (CH_2), 31.2 (CH_2), 31.1 (CH_2), 29.9 (CH_2), 29.7 (CH_2), 27.1 (CH_2), 23.7 (CH_2), 22.3 (CH_2), 19.7 (CH_2), 18.5 (CH_2); **HRMS** (NSI$^+$) Calc. for $C_{29}H_{40}O_5Na$ $[M+Na]^+$ 491.2773, found 491.2756.

S46: (1*S*,4*S*)-1,4-bis((2*R*,6*S*)-6-(4-(benzyloxy)butyl)tetrahydropyran-2-yl)-4-((*tert*-butyldimethylsilyl)oxy)but-2-yn-1-ol

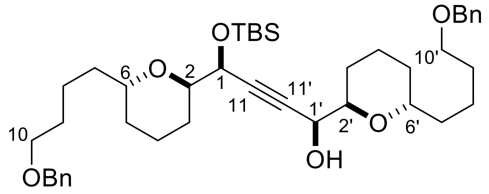

To a solution of alkyne **21** (841 mg, 2.02 mmol) in MTBE (15 mL) at 0°C was added *n*-BuLi (0.81 mL, 2.5 M solution in hexane, 2.02 mmol) and the reaction mixture was stirred for 10 min. The temperature was then lowered to -78°C and after further 10 min a solution of aldehyde **25** (109 mg, 0.394 mmol) in MTBE (5 mL) was added and the reaction mixture was allowed to warm to RT. The reaction was quenched by addition of saturated aqueous NH_4Cl (20 mL) and CH_2Cl_2 (10 mL). The organics were extracted with CH_2Cl_2 (3 x 20 mL), dried ($MgSO_4$), filtered and concentrated *in vacuo*. Purification by flash column chromatography (10→30% Et_2O/hexane) provided alkyne **S46** (198 mg, 73%) as a yellow oil, with >95:5 dr by ^{1}H NMR spectroscopy.

R_f 0.40 (50% Et_2O/Hexane); $[\alpha]_D^{20}$ -2.63 (*c* 1.0, $CHCl_3$); **IR** (thin film) 3420 (O-H), 2932 (C-H), 2857 (C-H), 1454 (aromatic C=C), 1362 (aromatic C=C), 1252 (C-O), 1204 (C-O), 1099 (C-O) cm^{-1}; **^{1}H NMR** (500 MHz, $CDCl_3$) δ 7.38 – 7.30 (8H, m, ArH), 7.30 – 7.24 (2H, m, ArH), 4.50 (4H, s, CH_2Ph), 4.41 – 4.35 (2H, m, H^1+$H^{1'}$), 3.92 - 3.85 (1H, m, $H^{6'}$), 3.82 – 3.71 (1H, m, H^6), 3.69 – 3.56 (2H, m, H^2+$H^{2'}$), 3.47 (2H, t, *J* = 6.5 Hz, $H^{10'}$), 3.46 (2H, t, *J* = 6.5 Hz, H^{10}), 2.79 (1H, d, *J* = 6.6 Hz, OH), 1.90 - 1.21 (24H, m, CH_2), 0.90 (9H, s, $SiC(CH_3)_3$), 0.16 (3H, s, $SiCH_3$), 0.12 (3H, s, $SiCH_3$); **^{13}C NMR** (126 MHz, $CDCl_3$) δ 138.7 (C(quat), ArC), 138.6 (C(quat), ArC), 128.5 (4 x CH, ArC), 127.9 (4 x CH, ArC), 127.8 (CH, ArC), 127.6 (CH, ArC), 86.0 (C(quat), CHC), 83.3 (C(quat), CHC), 73.7 (CH, C^2/$C^{2'}$), 73.0 (2 x CH_2, CH_2Ph), 72.9 (CH, $C^{6'}$), 72.6 (CH, C^6), 72.1 (CH, C^2/$C^{2'}$), 70.5 (2 x CH_2, C^{10} +$C^{10'}$), 65.0 (CH, C^1/$C^{1'}$), 64.9 (CH, C^1/$C^{1'}$), 32.3 (CH_2), 31.0 (CH_2), 29.8 (CH_2), 29.6 (CH_2), 28.6 (CH_2), 26.4 (CH_2), 25.9 (3 x CH_3, $SiC(CH_3)_3$), 25.4 (CH_2), 22.7 (CH_2), 22.6 (CH_2), 18.5 (CH_2), 18.3 (CH_2), 18.2 (CH_2), 18.0 (C(quat), $SiC(CH_3)_3$), -4.2 (CH_3, $SiCH_3$), -5.0 (CH_3, $SiCH_3$); **HRMS** (NSI$^+$) Calc. for $C_{42}H_{68}O_6Si_1N_1$ $[M+NH_4]^+$ 710.4810, found 710.4800.

33: (1*S*,4*S*)-1,4-bis((2*R*,6*S*)-6-(4-(benzyloxy)butyl)tetrahydropyran-2-yl)but-2-yne-1,4-diol

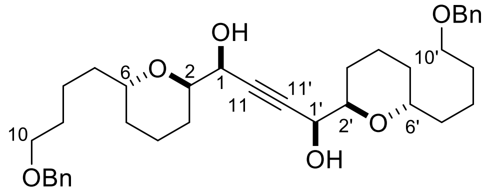

To a solution of alkyne **S46** (196 mg, 283 µmol) in methanol/CH_2Cl_2 (1:1, 10 mL) (±)-CSA (25 mg, 108 µmol) was added and stirred at RT. After 48 h, TLC analysis showed incomplete conversion, hence the reaction mixture was re-dosed with (±)-CSA (25 mg, 108 µmol) and stirred at RT for further 24 h. The reaction mixture was quenched by the addition of saturated aqueous $NaHCO_3$ (20 mL) and water (5 mL). The organics were extracted with CH_2Cl_2 (3 x 10 mL), dried ($MgSO_4$), filtered and concentrated *in vacuo*. Purification by flash column chromatography (70%→100% Et_2O/Hexane) provided diol **33** (132 mg, 80%) as a colourless oil.

R$_f$ 0.63 (100% Et_2O); $[\alpha]_D^{20}$ -27.2 (*c* 6.1, $CHCl_3$); **IR** (thin film) 3393 (O-H), 2936 (C-H), 2860 (C-H), 1454 (aromatic C=C), 1361 (aromatic C=C), 1098 (C-O), 1028 (C-O) cm^{-1}; **^1H NMR** (500 MHz, $CDCl_3$) δ 7.39 – 7.30 (8H, m, ArH), 7.30 - 7.22 (2H, m, ArH), 4.50 (4H, s, CH_2Ph), 4.45 - 4.33 (2H, m, H^1+$H^{1'}$), 3.93 - 3.83 (2H, m, H^6+$H^{6'}$), 3.69 - 3.61 (2H, m, H^2+$H^{2'}$), 3.47 (4H, t, *J* = 6.4 Hz, H^{10}+$H^{10'}$), 3.12 – 3.01 (2H, m, OH), 1.92 -1.23 (24H, m, CH_2); **^{13}C NMR** (126 MHz, $CDCl_3$) δ 138.6 (C(quat), ArC), 128.4 (CH, ArC), 127.8 (CH, ArC), 127.6 (CH, ArC), 84.5 (C(quat), CHC), 73.0 (CH_2, CH_2Ph), 72.9 (CH, C^6 +$C^{6'}$), 72.3 (CH, C^2+$C^{2'}$), 70.4 (CH_2, C^{10} +$C^{10'}$), 64.5 (CH, C^1+$C^{1'}$), 31.3 (CH_2), 29.7 (CH_2), 29.0 (CH_2), 25.6 (CH_2), 22.6 (CH_2), 18.2 (CH_2); **HRMS** (NSI^+) Calc. for $C_{36}H_{51}O_6$ $[M+H]^+$ 579.3680, found 579.3679.

12: 2,5-bis((2*R*,6*S*)-6-(4-(benzyloxy)butyl)tetrahydropyran-2-yl)furan

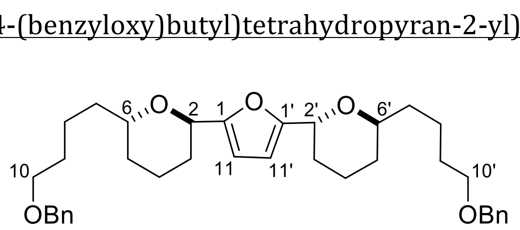

A solution of alkyne diol **33** (65.0 mg, 112 µmol), $Ru(PPh_3)_3(CO)H_2$ (5.2 mg, 5.7 µmol), xantphos (3.3 mg, 5.7 µmol) and benzoic acid (7.4 mg, 61 µmol) in toluene (3 mL) was heated in a sealed tube at 130°C. After 72 h, the reaction was quenched by the addition

of saturated aqueous $NaHCO_3$ (10 mL). The organics were extracted with CH_2Cl_2 (3 x 10 mL), dried ($MgSO_4$), filtered and concentrated *in vacuo*. Purification by flash column chromatography (20%→70% Et_2O/Hexane) provided furan **12** (22.3 mg, 36%) as a colourless oil.

R$_f$ 0.58 (50% Et_2O/Hexane); $[\alpha]_D^{20}$ -29.7 (*c* 1.5, $CHCl_3$); **IR** (thin film) 2934 (C-H), 2857 (C-H), 1454 (aromatic C=C), 1362 (aromatic C=C), 1099 (C-O), 1043 (C-O), 1016 (C-O) cm^{-1}; **^{1}H NMR** (400 MHz, $CDCl_3$) δ 7.38 – 7.31 (8H, m, ArH), 7.31 - 7.24 (2H, m, ArH), 6.20 (2H, s, H^{11}+$H^{11'}$), 4.93 (2H, dd, *J* = 5.0, 3.4 Hz, H^2+$H^{2'}$), 4.49 (2H, s, CH_2Ph), 3.59 - 3.50 (2H, m, H^6+$H^{6'}$), 3.46 (4H, t, *J* = 6.7 Hz, H^{10}+$H^{10'}$), 2.10 – 0.74 (24H, m, CH_2); **^{13}C NMR** (101 MHz, $CDCl_3$) δ 154.2 (C(quat), C^1+$C^{1'}$), 138.8 (C(quat), ArC), 128.5 (CH, ArC), 127.8 (CH, ArC), 127.6 (CH, ArC), 108.0 (CH, C^{11}+$C^{11'}$), 73.0 (CH_2, CH_2Ph), 71.5 (CH, C^6+$C^{6'}$), 70.5 (CH_2, C^{10}+$C^{10'}$), 68.8 (CH, C^2+$C^{2'}$), 35.3 (CH_2), 31.0 (CH_2), 29.9 (CH_2), 27.3 (CH_2), 22.4 (CH_2), 19.7 (CH_2); **HRMS** (NSI^+) Calc. for $C_{36}H_{48}O_5Na_1$ $[M+Na]^+$ 583.3394, found 583.3385.

S47: <u>(1*S*,4*S*)-4-((2*R*,6*S*)-6-(4-(benzyloxy)butyl)tetrahydropyran-2-yl)-4-((*tert*-butyldimethylsilyl)oxy)-1-((2*R*,6*S*)-6-(4-((*tert*-butyldiphenylsilyl)oxy)butyl)tetrahydropyran-2-yl)but-2-yn-1-ol</u>

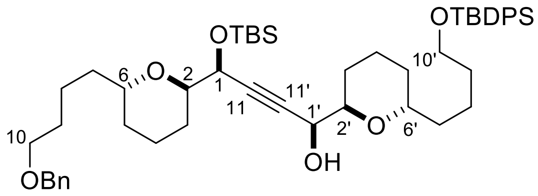

To a solution of alkyne **21** (500 mg, 1.20 mmol) in MTBE (15 mL) at 0°C was added *n*-BuLi (480 μL, 2.5 M solution in hexane, 1.20 mmol) and the reaction mixture was stirred for 10 min. The temperature was then lowered to -78°C and after further 10 min a solution of aldehyde **26**[1] (119 mg, 0.280 mmol) in MTBE (5 mL) was added and the reaction mixture was allowed to warm to RT. The reaction was quenched by addition of saturated aqueous NH_4Cl (20 mL) and CH_2Cl_2 (10 mL). The organics were extracted with CH_2Cl_2 (3 x 20 mL), dried ($MgSO_4$), filtered and concentrated *in vacuo*. Purification by flash column chromatography (20→30% Et_2O/hexane) provided alkyne **S47** (128 mg, 54%) as a yellow oil, with >95:5 dr by ^{1}H NMR spectroscopy.

R_f 0.50 (50% Et_2O/Hexane); $[\alpha]_D^{20}$ -1.88 (*c* 1.2, $CHCl_3$); **IR** (thin film) 3420 (O-H), 2930 (C-H), 2857 (C-H), 1458 (aromatic C=C), 1427 (aromatic C=C), 1252 (C-O), 1203 (C-O), 1103 (C-O), 1049 (C-O) cm^{-1}; **^{1}H NMR** (500 MHz, $CDCl_3$) δ 7.70 – 7.62 (4H, m, ArH), 7.44 – 7.29 (11H, m, ArH), 4.79 (2H, s, CH_2Ph), 4.39 – 4.34 (2H, m, H^1+$H^{1'}$), 3.91 - 3.83 (1H, m, $H^{6'}$), 3.83 – 3.74 (1H, m, H^6), 3.65 (2H, t, *J* = 6.4 Hz, $H^{10'}$), 3.68 – 3.56 (2H, m, H^2+$H^{2'}$), 3.46 (2H, t, *J* = 6.4 Hz, H^{10}), 2.71 (1H, d, *J* = 6.6 Hz, OH), 1.84 - 1.11 (24H, m, CH_2), 1.04 (9H, s, $SiC(CH_3)_3$), 0.89 (9H, s, $SiC(CH_3)_3$), 0.15 (3H, s, $SiCH_3$), 0.11 (3H, s, $SiCH_3$); **^{13}C NMR** (126 MHz, $CDCl_3$) δ 138.7 (C(quat), ArC), 135.7 (CH, ArC), 134.2 (C(quat), ArC), 129.7 (CH, ArC), 128.5 (CH, ArC), 127.9 (CH, ArC), 127.7 (CH, ArC), 127.6 (CH, ArC), 86.1 (C(quat), CHC), 83.3 (C(quat), CHC), 73.7 (CH, C^2/$C^{2'}$), 73.1 (CH, $C^{6'}$), 73.0 (CH_2, CH_2Ph), 72.6 (CH, C^6), 72.0 (CH, C^2/$C^{2'}$), 70.5 (CH_2, C^{10}), 65.1 (CH, C^1/$C^{1'}$), 65.0 (CH, C^1/$C^{1'}$), 64.0 (CH_2, $C^{10'}$), 32.7 (CH_2), 32.3 (CH_2), 31.8 (CH_2), 30.8 (CH_2), 29.8 (CH_2), 27.0 (3 x CH_3, $SiC(CH_3)_3$), 29.6 (CH_2), 26.4 (CH_2), 25.9 (3 x CH_3, $SiC(CH_3)_3$), 25.4 (CH_2), 22.6 (CH_2), 22.3 (CH_2), 19.4 (C(quat), $SiC(CH_3)_3$), 18.5 (C(quat), $SiC(CH_3)_3$), 18.3 (CH_2), 18.2 (CH_2), -4.2 (CH_3, $SiCH_3$), -4.9 (CH_3, $SiCH_3$); **HRMS** (NSI^+) Calc. for $C_{51}H_{80}O_6Si_2N_1$ $[M+NH_4]^+$ 858.5519, found 858.5513.

34: (1*S*,4*S*)-1-((2*R*,6*S*)-6-(4-(benzyloxy)butyl)tetrahydropyran-2-yl)-4-((2*R*,6*S*)-6-(4-hydroxybutyl)tetrahydropyran-2-yl)but-2-yne-1,4-diol

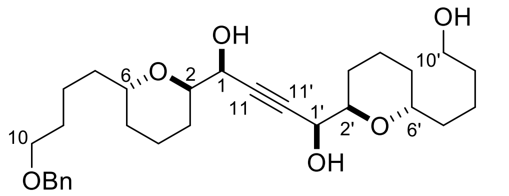

To a solution of alkyne **S47** (128 mg, 152 μmol) in water/THF (1:1, 16 mL) aqueous HCl (3M, 4 mL) was added and stirred at 50°C. After 6 h, TLC analysis showed incomplete conversion, hence the reaction mixture was re-dosed with HCl (3 M, 3 mL) and stirred at 50°C for 5 h. The temperature was increased to 90°C over further 5 h, then the reaction mixture was re-dosed with further HCl (3 M, 3 mL) and stirred at 90°C for 5 h and then at RT for 16 h. The reaction mixture was quenched by the addition of saturated aqueous $NaHCO_3$ (100 mL). The organics were extracted with CH_2Cl_2 (2 x 50 mL), dried ($MgSO_4$), filtered and concentrated *in vacuo*. Purification by flash column chromatography (100% Et_2O →100% EtOAc) provided triol **34** (50.5 mg, 68%) as a colourless oil.

R$_f$ 0.32 (100% EtOAc); $[\alpha]_D^{20}$ -33.2 (*c* 0.9, $CHCl_3$); **IR** (thin film) 3387 (O-H), 2938 (C-H), 2864 (C-H), 1456 (aromatic C=C), 1362 (aromatic C=C), 1215 (aromatic C=C), 1101 (C-O), 1042 (C-O) cm^{-1}; **^{1}H NMR** (500 MHz, $CDCl_3$) δ 7.41 – 7.22 (5H, m, ArH), 4.50 (2H, s, CH_2Ph), 4.45 - 4.33 (2H, m, H^1+$H^{1'}$), 3.88 - 3.76 (2H, m, H^6+$H^{6'}$), 3.71 - 3.58 (4H, m, H^2+$H^{2'}$+$H^{10'}$), 3.48 (2H, t, *J* = 6.4 Hz, H^{10}), 1.92 -1.16 (24H, m, CH_2); **^{13}C NMR** (126 MHz, $CDCl_3$) δ 138.5 (C(quat), ArC), 128.5 (CH, ArC), 127.8 (CH, ArC), 127.7 (CH, ArC), 84.9 (C(quat), CHC), 84.8 (C(quat), CHC), 73.1 (CH, C^2/$C^{2'}$), 73.0 (CH+CH_2, CH_2Ph+C^6 /$C^{6'}$), 72.9 (CH, C^6/$C^{6'}$), 72.8 (CH, C^2/$C^{2'}$), 70.5 (CH_2, C^{10}), 64.1 (CH, $C^{1'}$), 63.7 (CH, C^1), 62.5 (CH_2, $C^{10'}$), 32.4 (CH_2), 31.6 (CH_2), 30.4 (CH_2), 29.8 (CH_2), 29.7 (CH_2), 29.6 (CH_2), 29.3 (CH_2), 25.8 (CH_2), 22.7 (CH_2), 21.9 (CH_2), 18.3 (CH_2), 18.2 (CH_2); **HRMS** (NSI$^+$) Calc. for $C_{29}H_{48}O_6N_1$ $[M+NH_4]^+$ 506.3476, found 506.3466.

9: 4-((2*S*,6*R*)-6-(5-((2*R*,6*S*)-6-(4-(benzyloxy)butyl)tetrahydropyran-2-yl)furan-2-yl)tetrahydropyran-2-yl)butan-1-ol

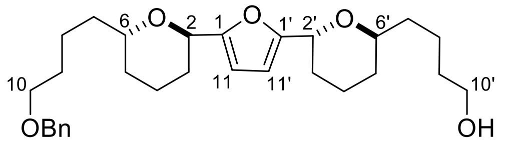

A solution of triol **34** (25.2 mg, 51.5 μmol), $Ru(PPh_3)_3(CO)H_2$ (4.2 mg, 4.6 μmol), xantphos (3.3 mg, 5.7 μmol) and benzoic acid (5.5 mg, 45 μmol) in toluene (3 mL) was heated at 130°C in a sealed tube. After 72 h, the reaction was quenched by the addition of saturated aqueous $NaHCO_3$ (10 mL). The organics were extracted with CH_2Cl_2 (3 x 10 mL), dried ($MgSO_4$), filtered and concentrated *in vacuo*. Purification by flash column chromatography (70%→100% Et_2O/Hexane) provided furan **9** (7.7 mg, 32%) as a colourless oil.

R$_f$ 0.29 (70% Et_2O/Hexane); $[\alpha]_D^{20}$ -37.0 (*c* 0.3, $CHCl_3$); **IR** (thin film) 3445 (O-H), 2936 (C-H), 2857 (C-H), 1717 (heteroaromatic C-O), 1454 (aromatic C=C), 1361 (aromatic C=C), 1105 (C-O), 1045 (C-O), 1016 (C-O) cm^{-1}; **^{1}H NMR** (500 MHz, $CDCl_3$) δ 7.38 – 7.22 (5H, m, ArH), 6.19 (2H, s, H^{11}+$H^{11'}$), 4.97 – 4.87 (2H, m, H^2+$H^{2'}$), 4.49 (2H, s, CH_2Ph), 3.61 (2H, t, *J* = 6.5 Hz, $H^{10'}$), 3.58 - 3.49 (2H, m, H^6+$H^{6'}$), 3.46 (4H, t, *J* = 6.6 Hz, H^{10}), 2.05 – 1.14 (24H, m, CH_2); **^{13}C NMR** (126 MHz, $CDCl_3$) δ 154.2 (2 x C(quat), C^1+$C^{1'}$), 138.8 (C(quat), ArC), 128.5 (CH, ArC), 127.8 (CH, ArC), 127.6 (CH, ArC), 108.1 (CH, C^{11}+$C^{11'}$), 73.0 (CH_2, CH_2Ph), 71.7 (CH, C^6/$C^{6'}$), 71.3 (CH, C^6/$C^{6'}$), 70.5 (CH_2, C^{10}), 68.8 (CH, C^2/$C^{2'}$), 68.7 (CH, C^2/$C^{2'}$), 63.1 (CH_2, $C^{10'}$), 35.1 (2 x CH_2), 32.7 (CH_2), 31.3 (CH_2), 30.9 (CH_2), 29.9 (CH_2), 27.3

(CH_2), 27.1 (CH_2), 22.4 (CH_2), 21.9 (CH_2), 19.8 (CH_2), 19.7 (CH_2); **HRMS** (NSI^+) Calc. for $C_{29}H_{42}O_5Na_1$ $[M+Na]^+$ 493.2924, found 493.2916.

S48: (1*S*/*R*,4*S*)-4-((2*R*,6*S*)-6-(4-(benzyloxy)butyl)tetrahydropyran-2-yl)-4-((*tert*-butyldimethylsilyl)oxy)-1-((2*S*,6*S*)-6-(4-((*tert*-butyldiphenylsilyl)oxy)butyl)tetrahydropyran-2-yl)but-2-yn-1-ol

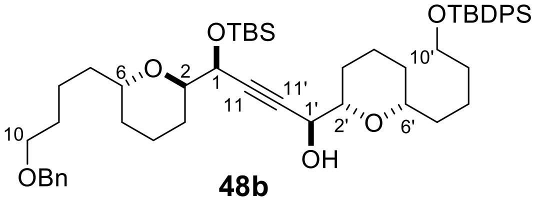

48b

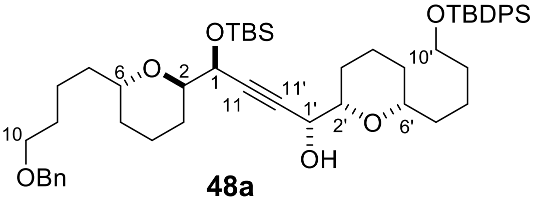

48a

To a solution of alkyne **21** (230 mg, 552 µmol) in MTBE (10 mL) at 0°C was added *n*-BuLi (0.22 mL, 2.5 M solution in hexane, 552 µmol) and the reaction mixture was stirred for 10 min. The temperature was then lowered to -78°C and after further 10 min a solution of aldehyde **24**[1] (113 mg, 266 µmol) in MTBE (5 mL) was added and the reaction mixture was allowed to warm to RT. The reaction was quenched by addition of saturated aqueous NH_4Cl (20 mL) and CH_2Cl_2 (10 mL). The organics were extracted with CH_2Cl_2 (3 x 20 mL), dried ($MgSO_4$), filtered and concentrated *in vacuo*. Purification by flash column chromatography (5→20% Et_2O/hexane) provided a separable mixture of **S48a** (38.4 mg) and **S48b** (17.0 mg) as colourless oils, with diastereomeric ratio 69:31 (total yield 35%).

S48a (major diastereomer): $\mathbf{R_f}$ 0.51 (50% Et_2O/Hexane); $[\alpha]_D^{20}$ -0.652 (*c* 0.9, $CHCl_3$); **IR** (thin film) 3428 (O-H), 2930 (C-H), 2857 (C-H), 1472 (aromatic C=C), 1427 (aromatic C=C), 1252 (C-O), 1103 (C-O), 1049 (C-O) cm^{-1}; ^{1}**H NMR** (500 MHz, $CDCl_3$) δ 7.72 – 7.61 (4H, m, ArH), 7.47 – 7.30 (10H, m, ArH), 7.30 – 7.20 (1H, m, ArH), 4.50 (2H, s, CH_2Ph), 4.43 (1H, dd, *J* = 7.4, 1.4 Hz, H^1), 4.39 – 4.35 (1H, m, $H^{1'}$), 3.78 - 3.71 (1H, m, H^6), 3.68 – 3.56 (1H, m, H^2), 3.65 (2H, t, *J* = 6.5 Hz, H^{10}), 3.47 (2H, t, *J* = 6.5 Hz, $H^{10'}$), 3.43 – 3.36 (1H, m, $H^{2'}$), 3.31 – 3.21 (1H, m, $H^{6'}$), 2.74 (1H, d, *J* = 6.4 Hz, OH), 1.88 - 1.09 (24H, m, CH_2), 1.05 (9H, s, $SiC(CH_3)_3$), 0.90 (9H, s, $SiC(CH_3)_3$), 0.15 (3H, s, $SiCH_3$), 0.12 (3H, s, $SiCH_3$); ^{13}**C NMR** (126 MHz, $CDCl_3$) δ 138.7 (C(quat), ArC), 135.7 (CH, ArC), 134.2 (C(quat), ArC), 129.6 (CH, ArC), 128.5 (CH, ArC), 127.8 (CH, ArC), 127.7 (CH, ArC), 127.6 (CH, ArC), 85.8 (C(quat), CHC), 83.1 (C(quat), CHC), 79.6 (CH, $C^{2'}$), 78.2 (CH, $C^{6'}$), 73.9 (CH, C^2), 73.0 (CH_2, CH_2Ph), 72.4 (CH, C^6), 70.6 (CH_2, $C^{10'}$), 65.3 (CH, $C^{1'}$), 64.8 (CH, C^1), 64.0 (CH_2, $C^{10'}$), 36.2 (CH_2), 32.8 (CH_2), 32.7 (CH_2), 31.4 (CH_2), 29.8 (CH_2), 29.8 (2 x CH_2), 27.0 (CH_3,

$SiC(CH_3)_3$), 26.0 (CH_2), 25.9 (CH_3, $SiC(CH_3)_3$), 25.6 (CH_2), 23.2 (CH_2), 22.6 (CH_2), 21.9 (CH_2), 19.4 (C(quat), $SiC(CH_3)_3$), 18.5 (C(quat), $SiC(CH_3)_3$), 18.3 (CH_2), -4.2 (CH_3, $SiCH_3$), -4.9 (CH_3, $SiCH_3$); **HRMS** (NSI^+) Calc. for $C_{51}H_{80}O_6Si_2N_1$ $[M+NH_4]^+$ 858.5519, found 858.5510.

S48b (minor diasateromer): **R$_f$** 0.57 (50% Et_2O/Hexane); $[\alpha]_D^{20}$ +3.07 (*c* 1.0, $CHCl_3$); **IR** (thin film) 3433 (O-H), 2930 (C-H), 2857 (C-H), 1472 (aromatic C=C), 1427 (aromatic C=C), 1252 (C-O), 1103 (C-O), 1049 (C-O) cm^{-1}; **^{1}H NMR** (500 MHz, $CDCl_3$) δ 7.71 – 7.62 (4H, m, ArH), 7.45 – 7.29 (10H, m, ArH), 7.29 – 7.23 (1H, m, ArH), 4.49 (2H, s, CH_2Ph), 4.39 (1H, dd, *J* = 7.3, 1.5 Hz, H^1), 4.20 (1H, d, *J* = 7.7 Hz, $H^{1'}$), 3.80-3.71 (1H, m, H^6), 3.69 – 3.53 (1H, m, H^2), 3.64 (2H, t, *J* = 6.6 Hz, $H^{10'}$), 3.47 (2H, t, *J* = 6.6 Hz, H^{10}), 3.35 – 3.21 (2H, m, $H^{2'}$+$H^{6'}$), 2.90 (1H, s, OH), 1.96 - 1.10 (24H, m, CH_2), 1.04 (9H, s, $SiC(CH_3)_3$), 0.89 (9H, s, $SiC(CH_3)_3$), 0.15 (3H, s, $SiCH_3$), 0.11 (3H, s, $SiCH_3$); **^{13}C NMR** (126 MHz, $CDCl_3$) δ 138.7 (C(quat), ArC), 135.7 (CH, ArC), 134.2 (C(quat), ArC), 129.7 (CH, ArC), 128.5 (CH, ArC), 127.8 (CH, ArC), 127.7 (CH, ArC), 127.6 (CH, ArC), 86.1 (C(quat), CHC), 82.8 (C(quat), CHC), 80.6 (CH, $C^{2'}$), 78.1 (CH, $C^{6'}$), 73.8 (CH, C^2), 73.0 (CH_2, CH_2Ph), 72.5 (CH, C^6), 70.5 (CH_2, $C^{10'}$), 66.2 (CH, $C^{1'}$), 64.9 (CH, C^1), 63.9 (CH_2, C^{10}), 36.3 (CH_2), 32.7 (CH_2), 31.4 (CH_2), 29.9 (CH_2), 29.7 (CH_2), 27.5 (CH_2), 27.4 (CH_2), 27.0 (CH_3, $SiC(CH_3)_3$), 26.2 (CH_2), 25.9 (CH_3, $SiC(CH_3)_3$), 23.3 (CH_2), 22.5 (CH_2), 21.9 (CH_2), 19.4 (C(quat), $SiC(CH_3)_3$), 18.5 (C(quat), $SiC(CH_3)_3$), 18.3 (CH_2), -4.2 (CH_3, $SiCH_3$), -4.9 (CH_3, $SiCH_3$); **HRMS** (NSI^+) Calc. for $C_{51}H_{80}O_6Si_2N_1$ $[M+NH_4]^+$ 858.5519, found 858.5512.

35: (1*S*)-1-((2*R*,6*S*)-6-(4-(benzyloxy)butyl)tetrahydropyran-2-yl)-4-((2*S*,6*S*)-6-(4-hydroxybutyl)tetrahydropyran-2-yl)but-2-yne-1,4-diol

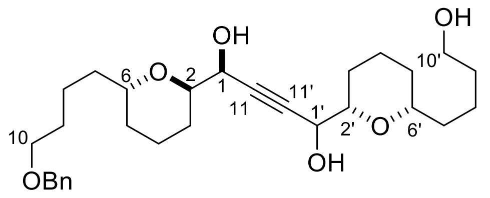

To a solution of alkyne **S48** (120 mg, 143 μmol) in methanol/CH_2Cl_2 (1:1, 10 mL) was added (±)-CSA (13 mg, 57 μmol). The reaction mixture was stirred at RT for 48 h, after which it was quenched by the addition of saturated aqueous $NaHCO_3$ (10 mL) and water (20 mL). The organics were extracted with CH_2Cl_2 (3 x 10 mL), dried ($MgSO_4$), filtered and concentrated *in vacuo*. Purification by flash column chromatography (100% EtOAc) provided triol **35** (14.7 mg, 21%) as a colourless oil.

R_f 0.40 (100% EtOAc); $[\alpha]_D^{20}$ -22.7 (*c* 0.2, $CHCl_3$); **IR** (thin film) 3385 (O-H), 2934 (C-H), 2860 (C-H), 1454 (aromatic C=C), 1364 (aromatic C=C), 1204 (C-O), 1099 (C-O), 1045 (C-O) cm^{-1}; **^{1}H NMR** (400 MHz, $CDCl_3$) δ 7.39 - 7.31 (4H, m, ArH), 7.30-7.24 (1H, m, ArH), 4.50 (2H, s, CH_2Ph), 4.40 (1H, br s, H^1), 4.34 (1H, br s, $H^{1'}$), 3.93 - 3.84 (1H, m, H^6), 3.71 - 3.58 (1H, m, H^2), 3.64 (2H, t, *J* = 6.1 Hz, $H^{10'}$), 3.51 - 3.39 (1H, m, $H^{2'}$), 3.47 (2H, t, *J* = 6.5 Hz, H^{10}), 3.38 - 3.29 (1H, m, $H^{6'}$), 3.05 (1H, br s, OH), 2.92 (1H, br s, OH), 2.09 - 1.09 (24H, m, CH_2); **^{13}C NMR** (101 MHz, $CDCl_3$) δ 138.6 (C(quat), ArC), 128.5 (CH, ArC), 127.8 (CH, ArC), 127.7 (CH, ArC), 84.4 (C(quat), CHC), 84.3 (C(quat), CHC), 79.8 (CH, $C^{2'}$), 78.4 (CH, $C^{6'}$), 73.1 (CH_2, CH_2Ph + CH, C^6), 72.4 (CH, C^2), 70.5 (CH_2, $C^{10'}$), 65.4 (CH, $C^{1'}$), 64.6 (CH, C^1), 62.8 (CH_2, C^{10}), 35.5 (CH_2), 32.5 (CH_2), 31.5 (CH_2), 31.3 (CH_2), 29.7 (CH_2), 29.0 (CH_2), 26.2 (CH_2), 25.6 (CH_2), 23.2 (CH_2), 22.8 (CH_2), 21.8 (CH_2), 18.2 (CH_2); **HRMS** (NSI^+) Calc. for $C_{29}H_{45}O_6$ $[M+H]^+$ 489.3211, found 489.3200.

8: 4-((2*S*,6*S*)-6-(5-((2*R*,6*S*)-6-(4-(benzyloxy)butyl)tetrahydropyran-2-yl)furan-2-yl)tetrahydropyran-2-yl)butan-1-ol

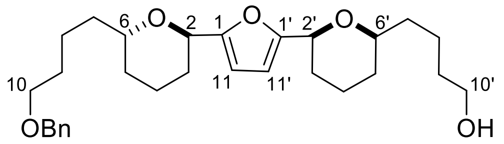

A solution of alkyne triol **35** (12.6 mg, 25.8 μmol), $Ru(PPh_3)_3(CO)H_2$ (3.5 mg, 3.8 μmol), xantphos (6.5 mg, 11.2 μmol) and benzoic acid (9.1 mg, 74.5 μmol) in toluene (3 mL) was heated in a sealed tube at 130°C. After 43 h, the reaction mixture was quenched by the addition of saturated aqueous $NaHCO_3$ (10 mL). The organics were extracted with CH_2Cl_2 (4 x 10 mL), dried ($MgSO_4$), filtered and concentrated *in vacuo*. Purification by flash column chromatography (70% Et_2O/Hexane) provided furan **8** (4.4 mg, 36%) as a colourless oil.

R_f 0.27 (70% Et_2O); $[\alpha]_D^{20}$ -24.4 (*c* 0.4, $CHCl_3$); **IR** (thin film) 3428 (O-H), 2936 (C-H), 2859 (C-H), 1437 (aromatic C=C), 1094 (C-O), 1043 (C-O), 1028 (C-O) cm^{-1}; **^{1}H NMR** (500 MHz, $CDCl_3$) δ 7.37-7.30 (4H, m, ArH), 7.30 – 7.24 (1H, m, ArH), 6.21 (1H, d, *J* = 3.2 Hz, $H^{11}/H^{11'}$), 6.16 (1H, d, *J* = 2.9 Hz, $H^{11}/H^{11'}$), 4.96 – 4.87 (1H, m, H^2), 4.49 (4H, s, CH_2Ph), 4.37 (1H, dd, *J* = 11.4, 1.9 Hz, $H^{2'}$), 3.66 – 3.58 (2H, m, $H^{10'}$), 3.56 - 3.38 (2H, m, $H^{6'}$ + H^6), 3.46 (2H, t, *J* = 6.6 Hz, H^{10}), 2.06-1.16 (24H, m, CH_2); **^{13}C NMR** (126 MHz, $CDCl_3$) δ 155.0 (C(quat), $C^1/C^{1'}$), 154.2 (C(quat), $C^1/C^{1'}$), 138.8 (C(quat), ArC),128.5 (CH, ArC), 127.8 (CH, ArC), 127.6 (CH, ArC), 108.1 (CH, $C^{11}/C^{11'}$), 106.6 (CH, $C^{11}/C^{11'}$), 78.4 (CH, $C^{6'}$),

73.5 (CH, $C^{2'}$), 73.0 (CH_2, CH_2Ph), 71.6 (CH, C^6), 70.5 (CH_2, C^{10}), 68.7 (CH, C^2), 63.0 (CH_2, $C^{10'}$), 36.2 (CH_2), 35.1 (CH_2), 32.9 (CH_2), 31.3 (CH_2), 30.9 (CH_2), 29.9 (CH_2), 29.6 (CH_2), 27.3 (CH_2), 23.7 (CH_2), 22.3 (CH_2), 21.9 (CH_2), 19.6 (CH_2); **HRMS** (ESI^+) Calc. for $C_{29}H_{42}O_5Na$ $[M+Na]^+$ 493.2924, found 493.2916.

References:

1. Florence, G. J.; Fraser, A. L.; Gould, E. R.; King, E. F. B.; Menzies, S. K.; Morris, J. C.; Tulloch, L. B.; Smith, T. K. *ChemMedChem* **2014**, *9*, 2548–2556.

2. (a) Pridmore, S. J.; Slatford, P. A.; Taylor, J. E.; Whittlesey, M. K.; Williams, J. M. J. *Tetrahedron* **2009**, 65, 8981–8986; (b) Pridmore, S. J. Ruthenium Catalysed Sequential and Tandem Reactions (PhD thesis), University of Bath, 2009.

3. (a) Florence, G. J.; Morris, J. C.; Murray, R. G.; Osler, J. D.; Reddy, V. R.; Smith, T. K. *Org. Lett* **2011**, *13*, 514–517; (b) Florence, G. J.; Morris, J. C.; Murray, R. G.; Vanga, R. R.; Osler, J. D.; Smith, T. K. *Chem. Eur. J.* **2013**, *19*, 8309–8320.

^{1}H and ^{13}C NMR Spectra

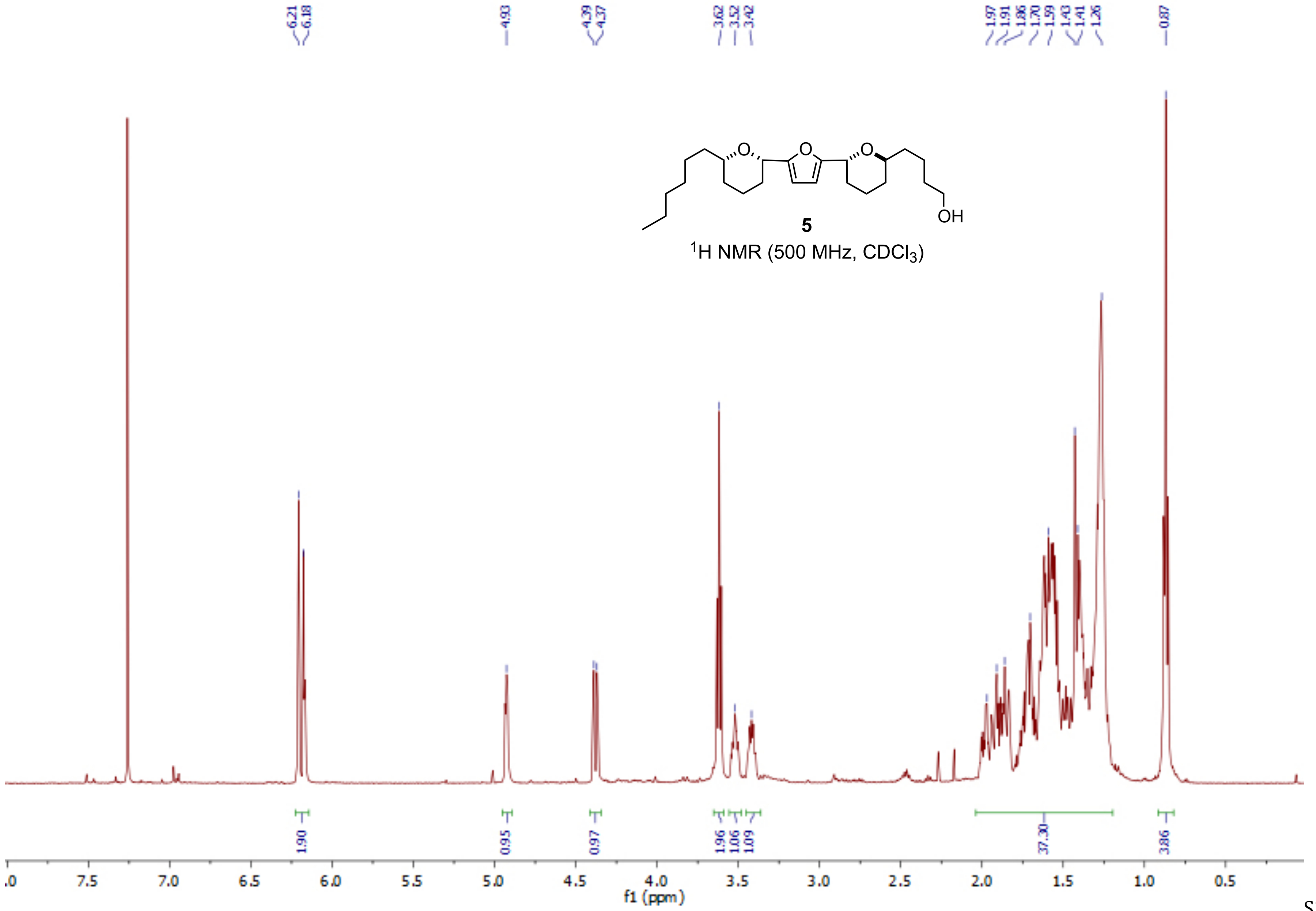

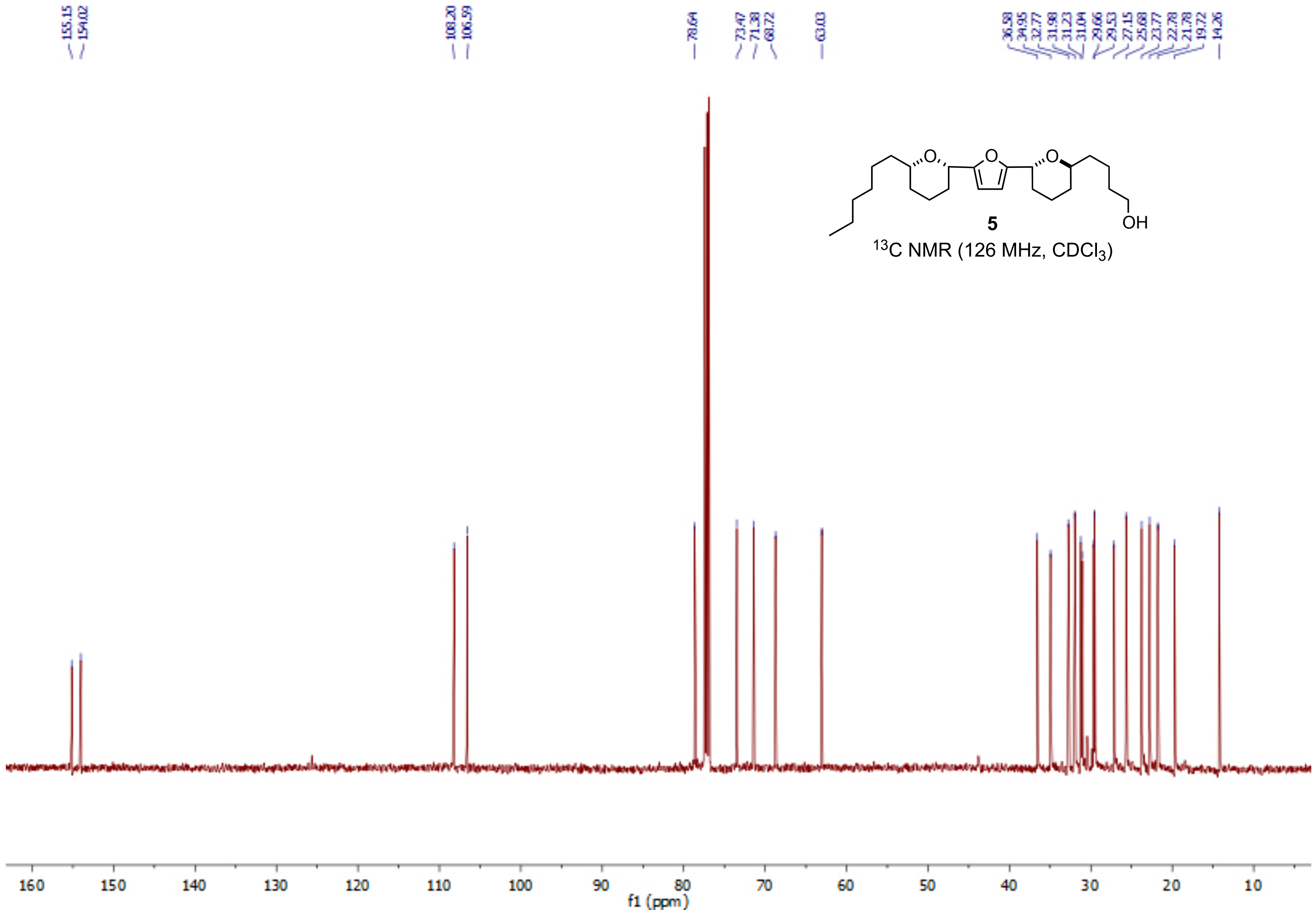
155.15
154.02
108.20
106.59
78.64
73.47
71.38
68.72
63.03
36.58
34.95
32.77
31.98
31.23
31.04
29.66
29.53
27.15
25.68
23.77
22.78
21.78
19.72
14.26
O
O
O
OH
5
^{13}C NMR (126 MHz, $CDCl_3$)
160
150
140
130
120
110
100
90
80
70
60
50
40
30
20
10
f1 (ppm)

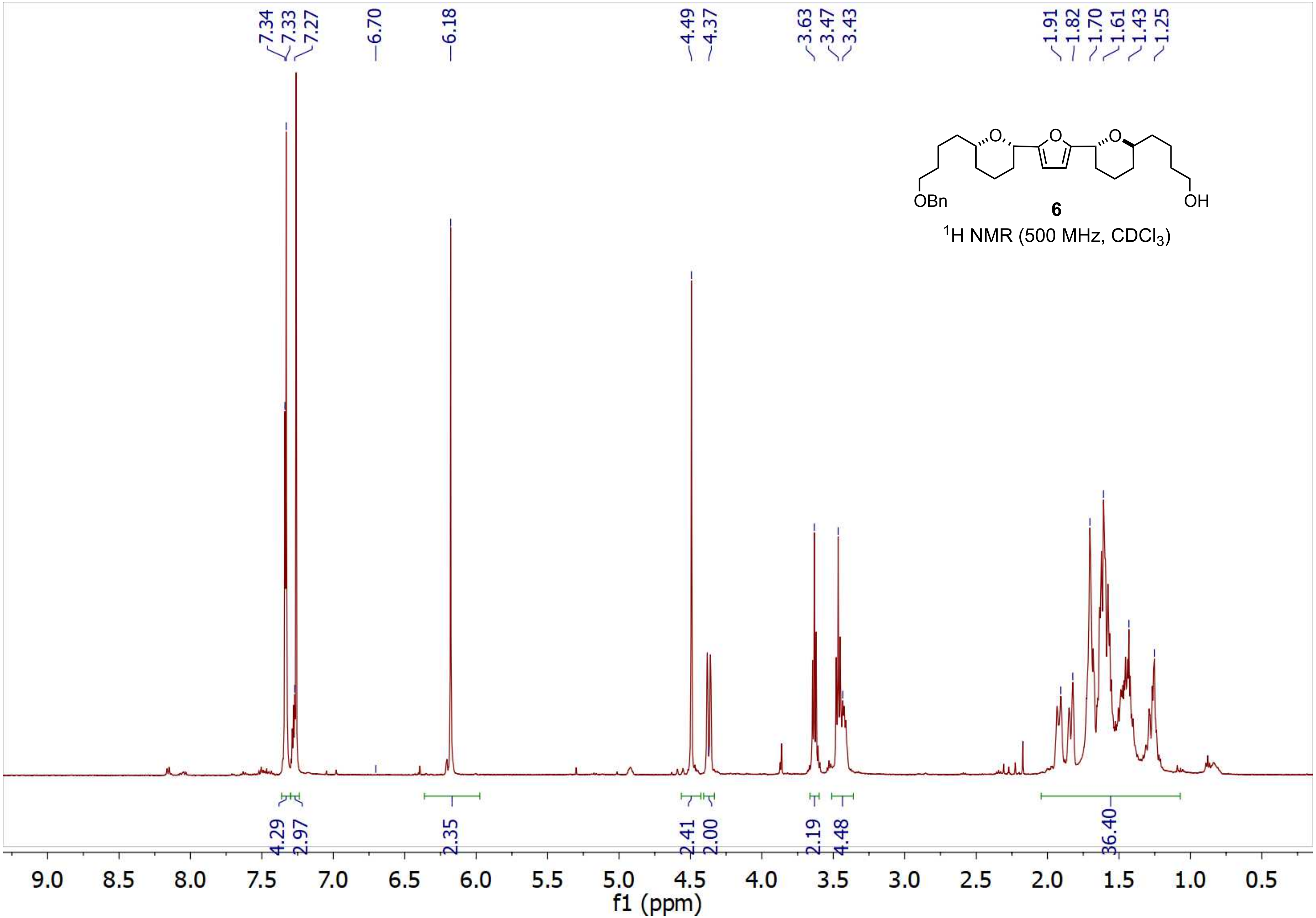
7.34
7.33
7.27
6.70
6.18
4.49
4.37
3.63
3.47
3.43
1.91
1.82
1.70
1.61
1.43
1.25
OBn
6
OH
^{1}H NMR (500 MHz, CDCl$_3$)
4.29
2.97
2.35
2.41
2.00
2.19
4.48
36.40
9.0
8.5
8.0
7.5
7.0
6.5
6.0
5.5
5.0
4.5
4.0
3.5
3.0
2.5
2.0
1.5
1.0
0.5
f1 (ppm)

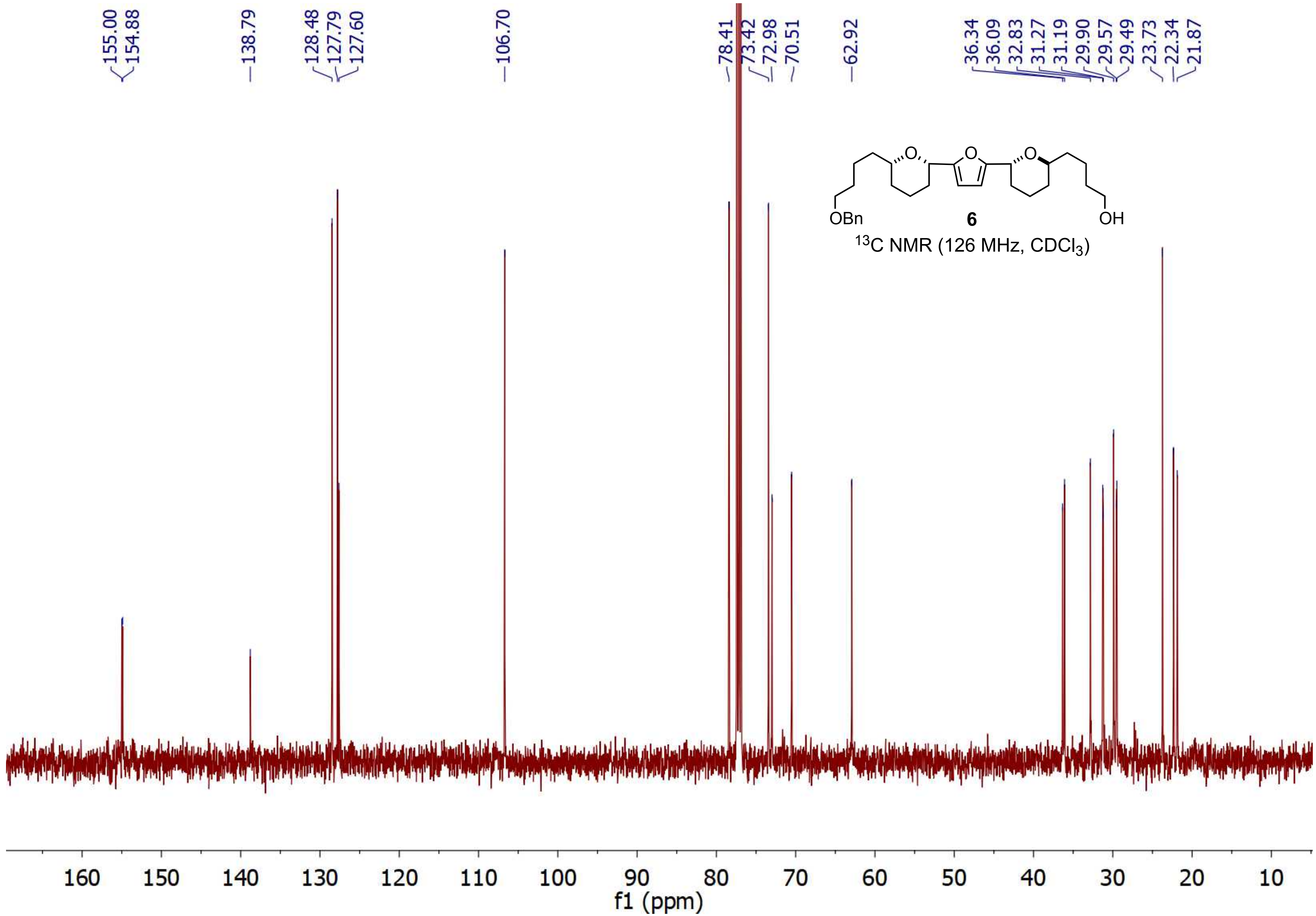
155.00
154.88
138.79
128.48
127.79
127.60
106.70
78.41
73.42
72.98
70.51
62.92
36.34
36.09
32.83
31.27
31.19
29.90
29.57
29.49
23.73
22.34
21.87
OBn
6
OH
^{13}C NMR (126 MHz, $CDCl_3$)
160 150 140 130 120 110 100 90 80 70 60 50 40 30 20 10
f1 (ppm)

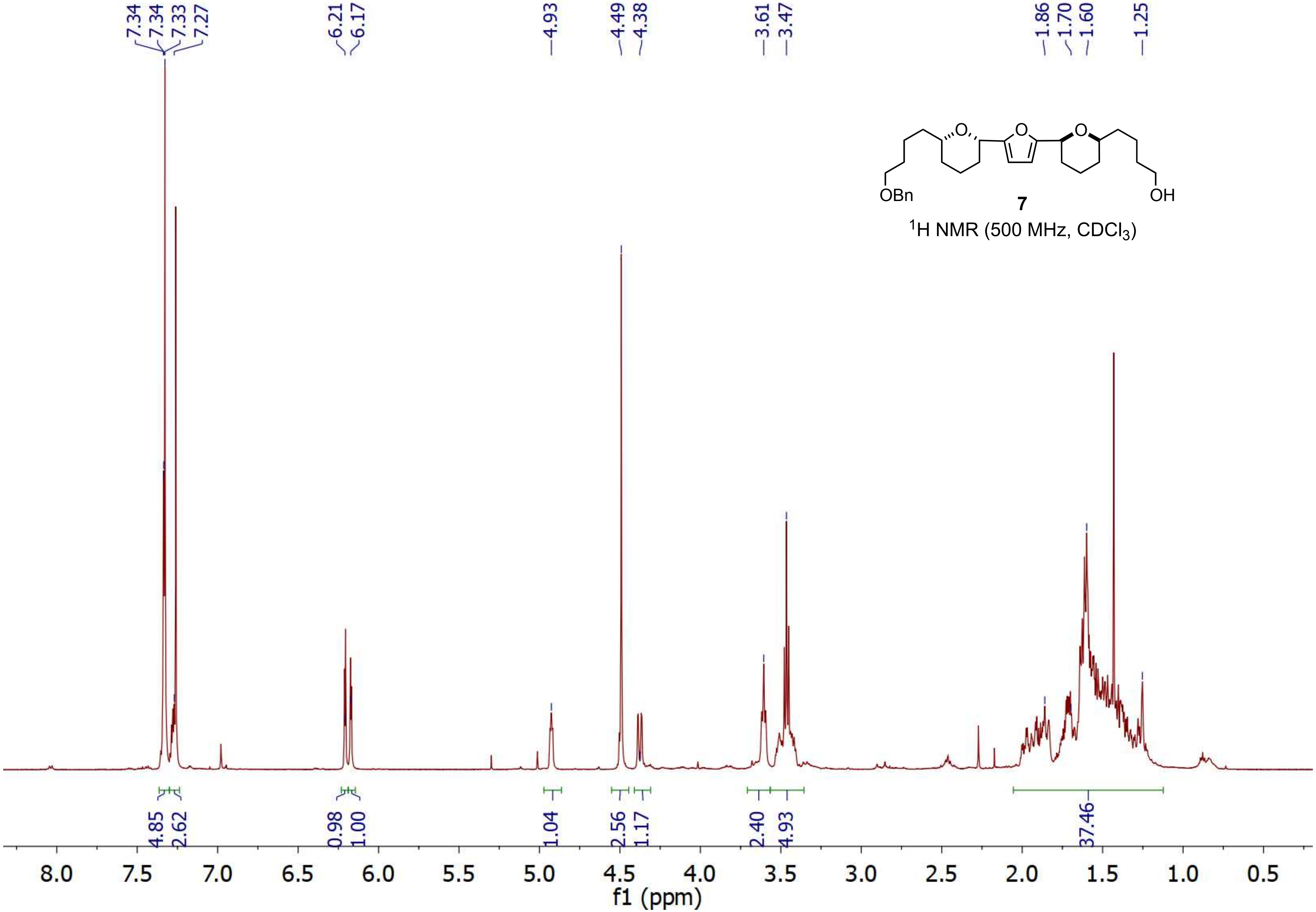

7.34
7.34
7.33
7.27
6.21
6.17
4.93
4.49
4.38
3.61
3.47
1.86
1.70
1.60
1.25
OBn
OH
7
^{1}H NMR (500 MHz, $CDCl_3$)
4.85
2.62
0.98
1.00
1.04
2.56
1.17
2.40
4.93
37.46
8.0
7.5
7.0
6.5
6.0
5.5
5.0
4.5
4.0
3.5
3.0
2.5
2.0
1.5
1.0
0.5
f1 (ppm)

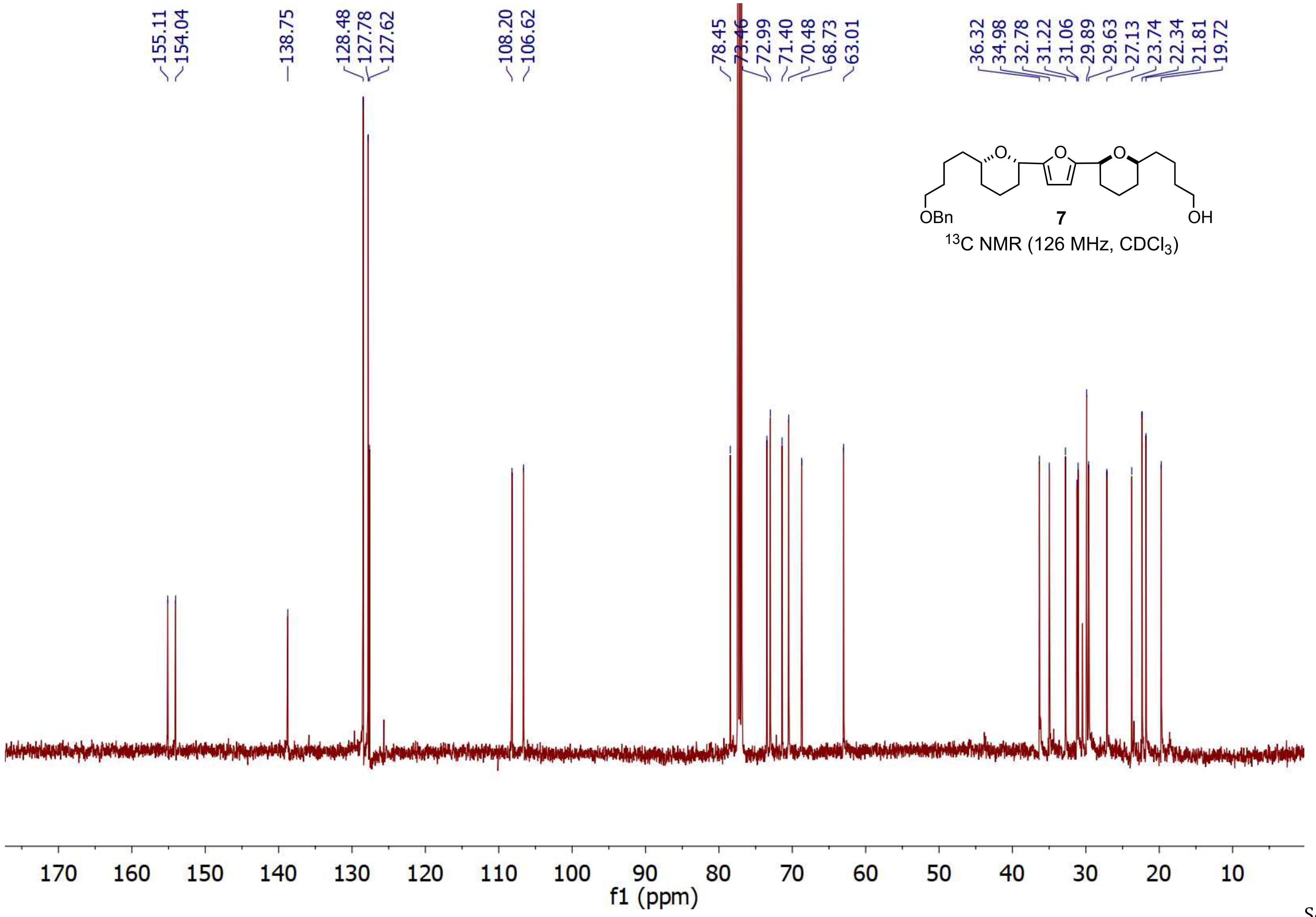
155.11
154.04
138.75
128.48
127.78
127.62
108.20
106.62
78.45
73.46
72.99
71.40
70.48
68.73
63.01
36.32
34.98
32.78
31.22
31.06
29.89
29.63
27.13
23.74
22.34
21.81
19.72
OBn
7
OH
^{13}C NMR (126 MHz, CDCl$_3$)
170 160 150 140 130 120 110 100 90 80 70 60 50 40 30 20 10
f1 (ppm)

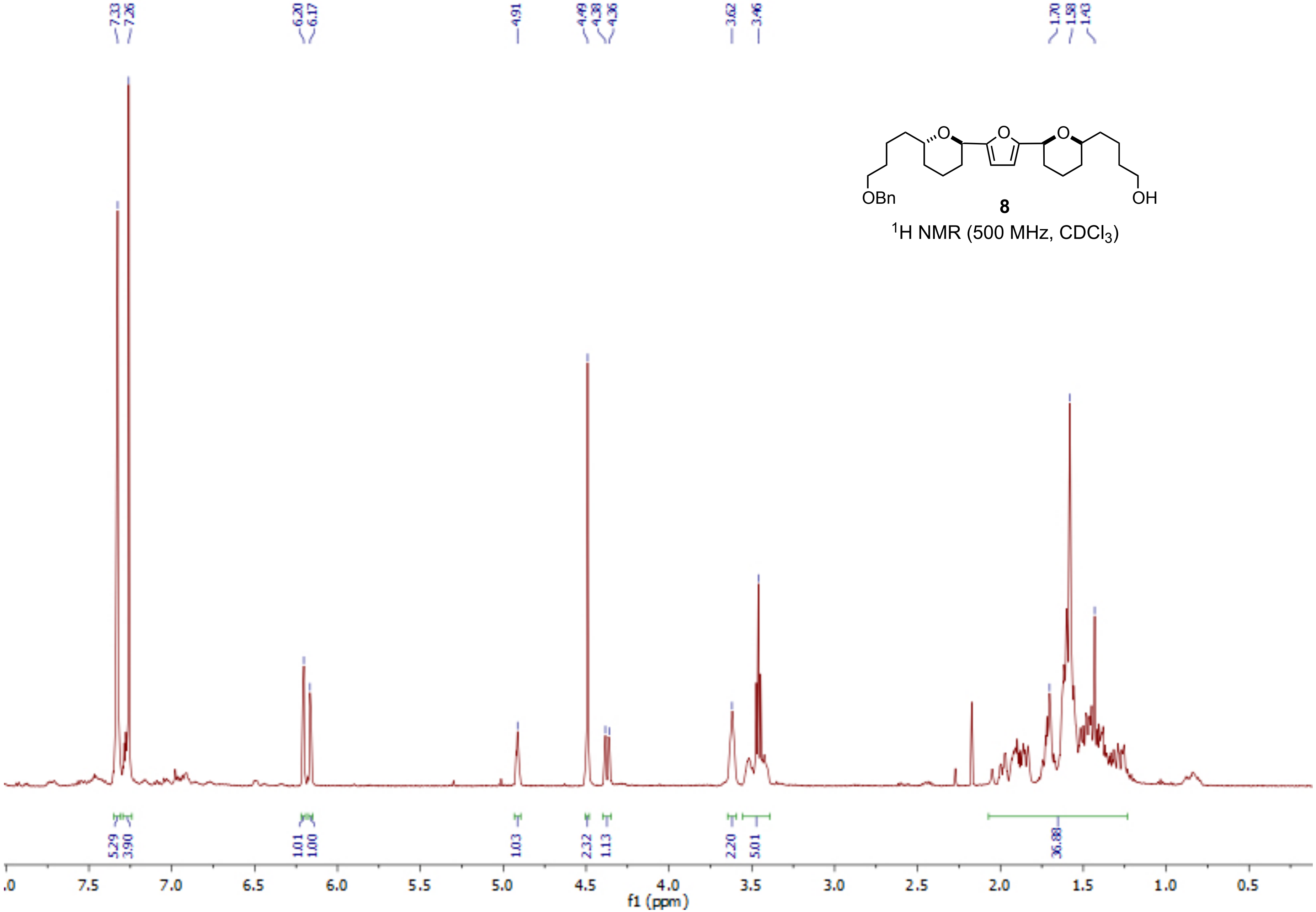

8

^{1}H NMR (500 MHz, $CDCl_3$)

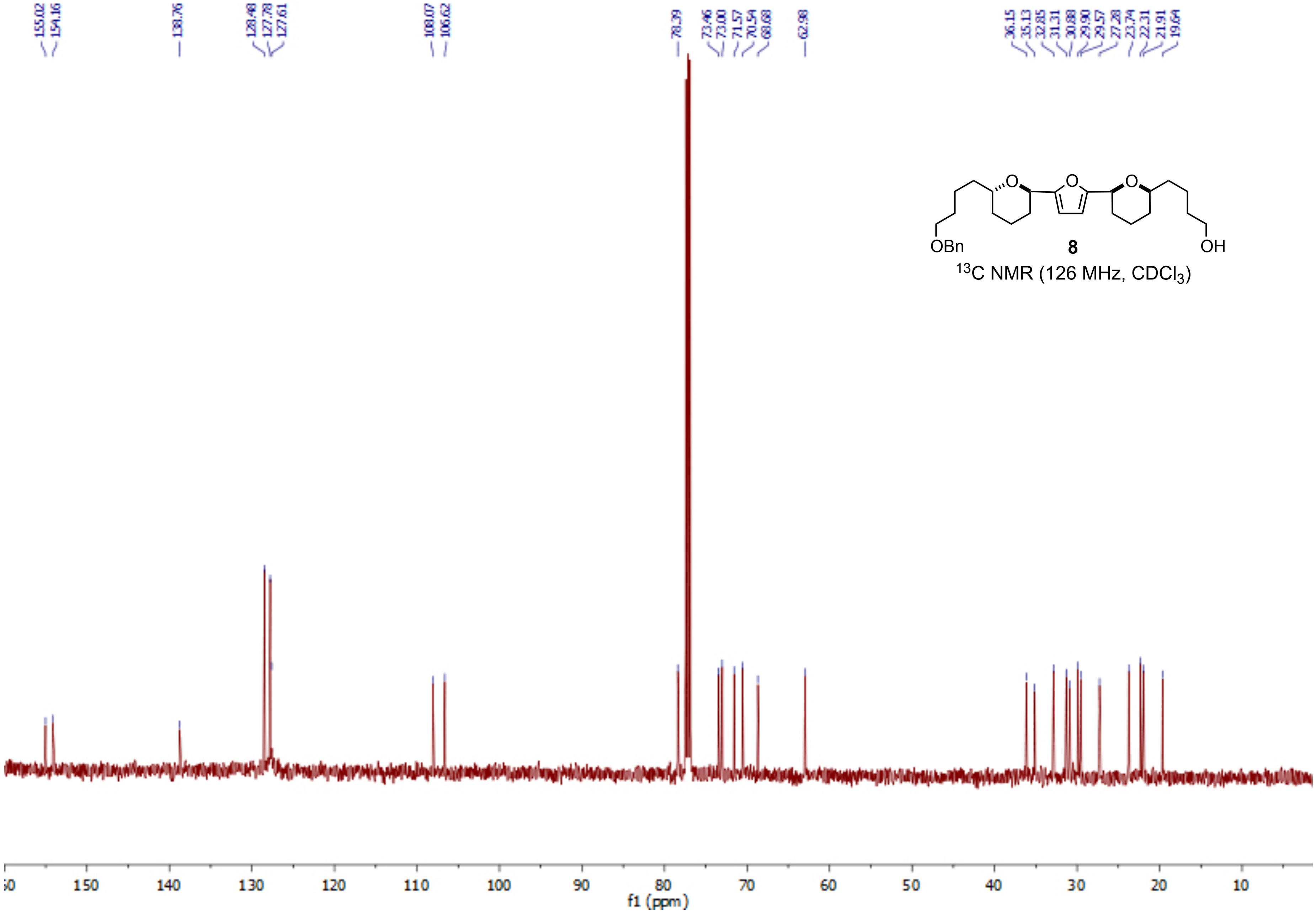
155.02
154.16
138.76
128.48
127.78
127.61
108.07
106.62
78.39
73.46
73.00
71.57
70.54
68.68
62.98
36.15
35.13
32.85
31.31
30.88
29.90
29.57
27.28
23.74
22.31
21.91
19.64
OBn
8
OH
^{13}C NMR (126 MHz, $CDCl_3$)
150
140
130
120
110
100
90
80
70
60
50
40
30
20
10
f1 (ppm)

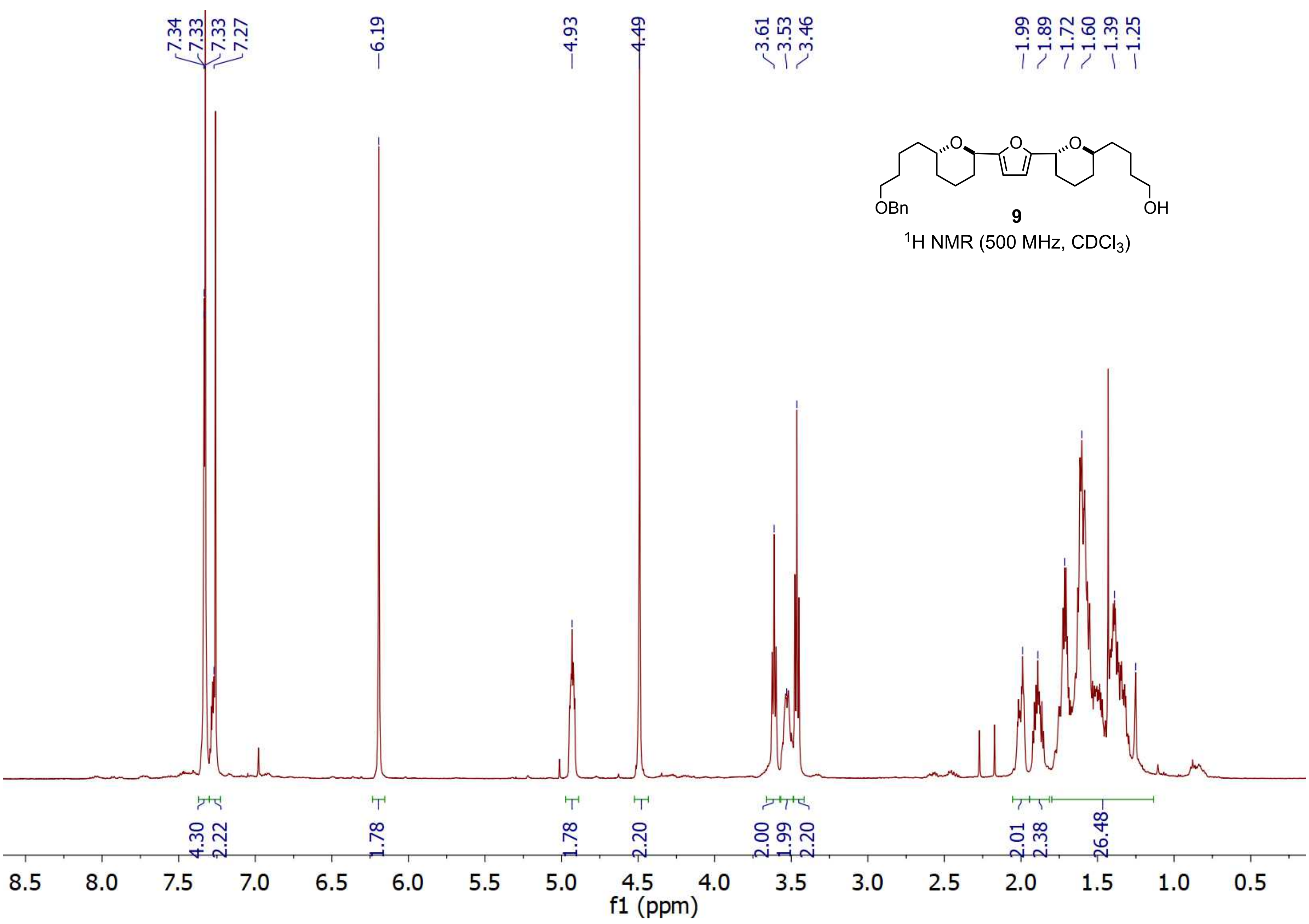

7.34
7.33
7.33
7.27
6.19
4.93
4.49
3.61
3.53
3.46
1.99
1.89
1.72
1.60
1.39
1.25
OBn
OH
O
9
^{1}H NMR (500 MHz, $CDCl_3$)
4.30
2.22
1.78
1.78
2.20
2.00
1.99
2.20
2.01
2.38
26.48
8.5
8.0
7.5
7.0
6.5
6.0
5.5
5.0
4.5
4.0
3.5
3.0
2.5
2.0
1.5
1.0
0.5
f1 (ppm)

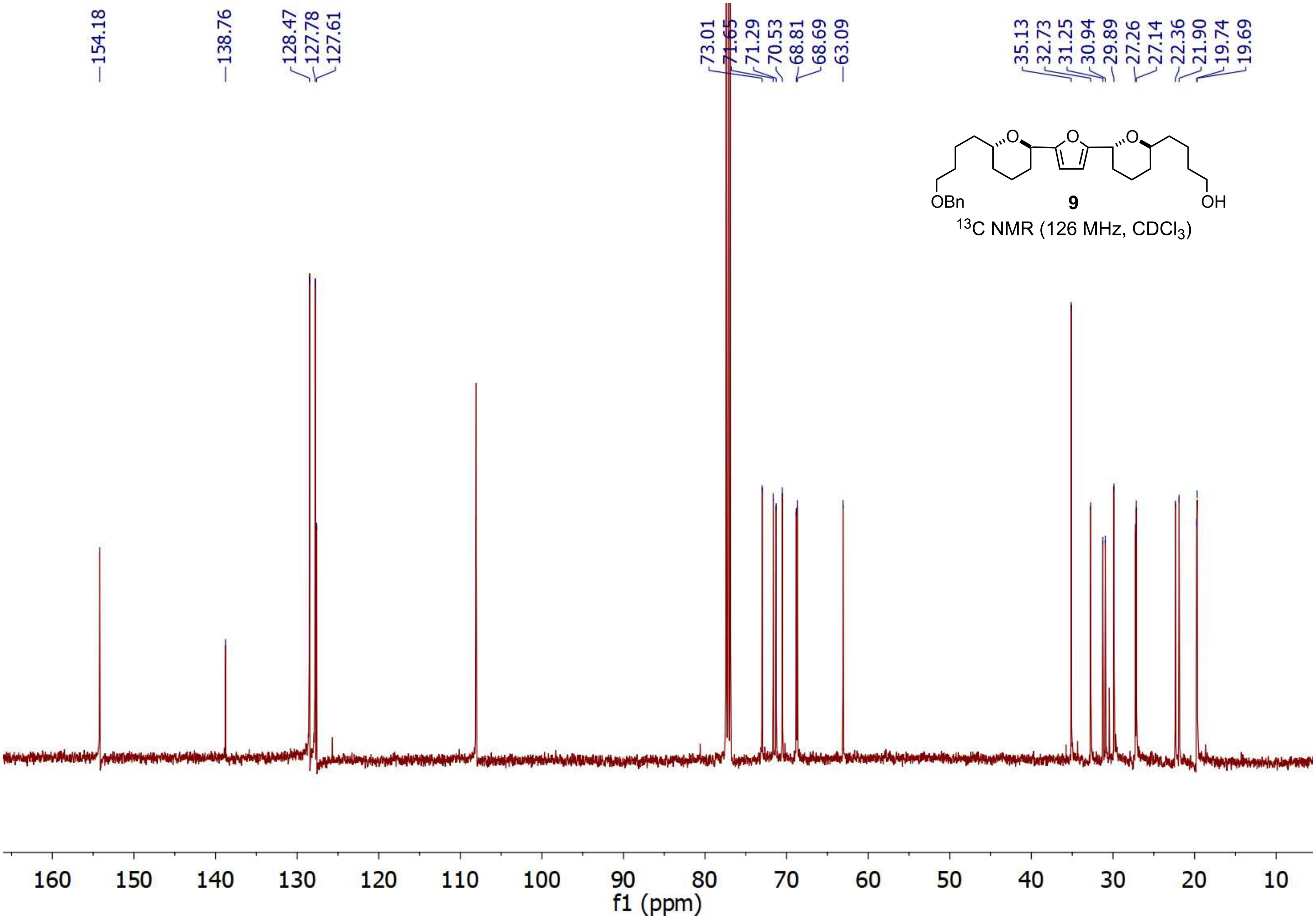
154.18
138.76
128.47
127.78
127.61
73.01
71.65
71.29
70.53
68.81
68.69
63.09
35.13
32.73
31.25
30.94
29.89
27.26
27.14
22.36
21.90
19.74
19.69
OBn
9
OH
^{13}C NMR (126 MHz, $CDCl_3$)
160 150 140 130 120 110 100 90 80 70 60 50 40 30 20 10
f1 (ppm)

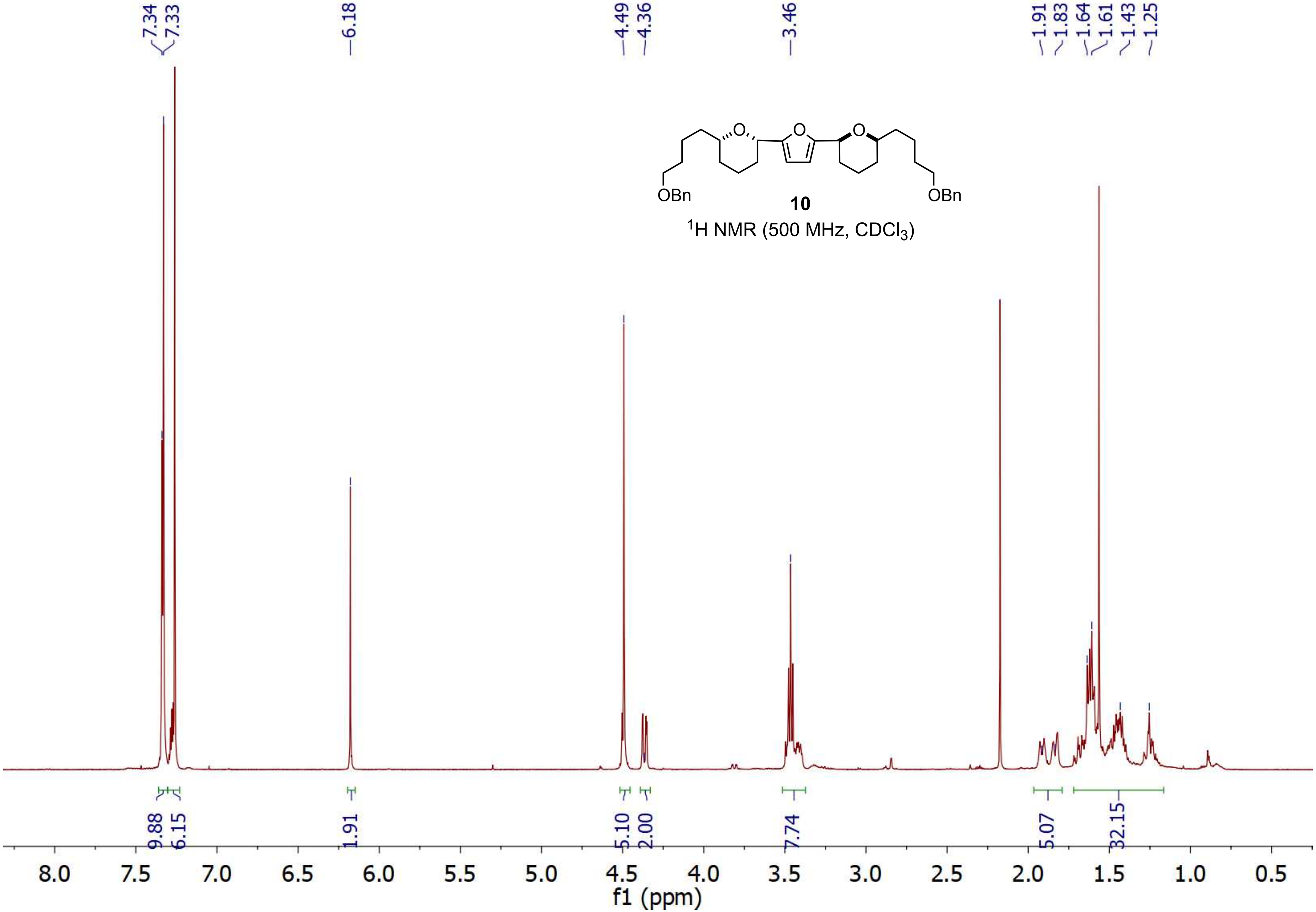
7.34
7.33
6.18
4.49
4.36
3.46
1.91
1.83
1.64
1.61
1.43
1.25
OBn
OBn
10
1H NMR (500 MHz, CDCl3)
9.88
6.15
1.91
5.10
2.00
7.74
5.07
32.15
8.0 7.5 7.0 6.5 6.0 5.5 5.0 4.5 4.0 3.5 3.0 2.5 2.0 1.5 1.0 0.5
f1 (ppm)

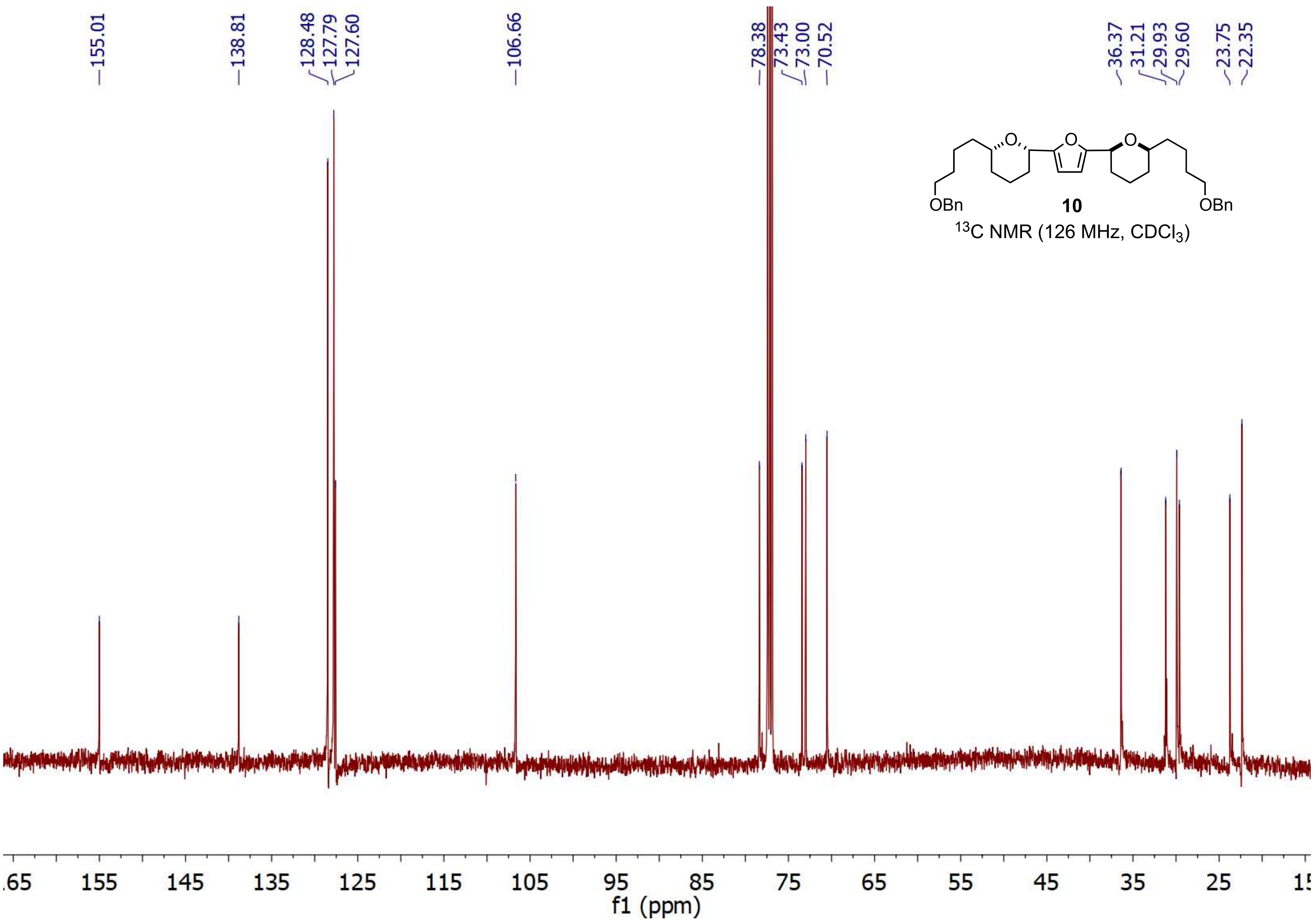
155.01
138.81
128.48
127.79
127.60
106.66
78.38
73.43
73.00
70.52
36.37
31.21
29.93
29.60
23.75
22.35
OBn
10
OBn
13C NMR (126 MHz, CDCl3)
165
155
145
135
125
115
105
95
85
75
65
55
45
35
25
15
f1 (ppm)

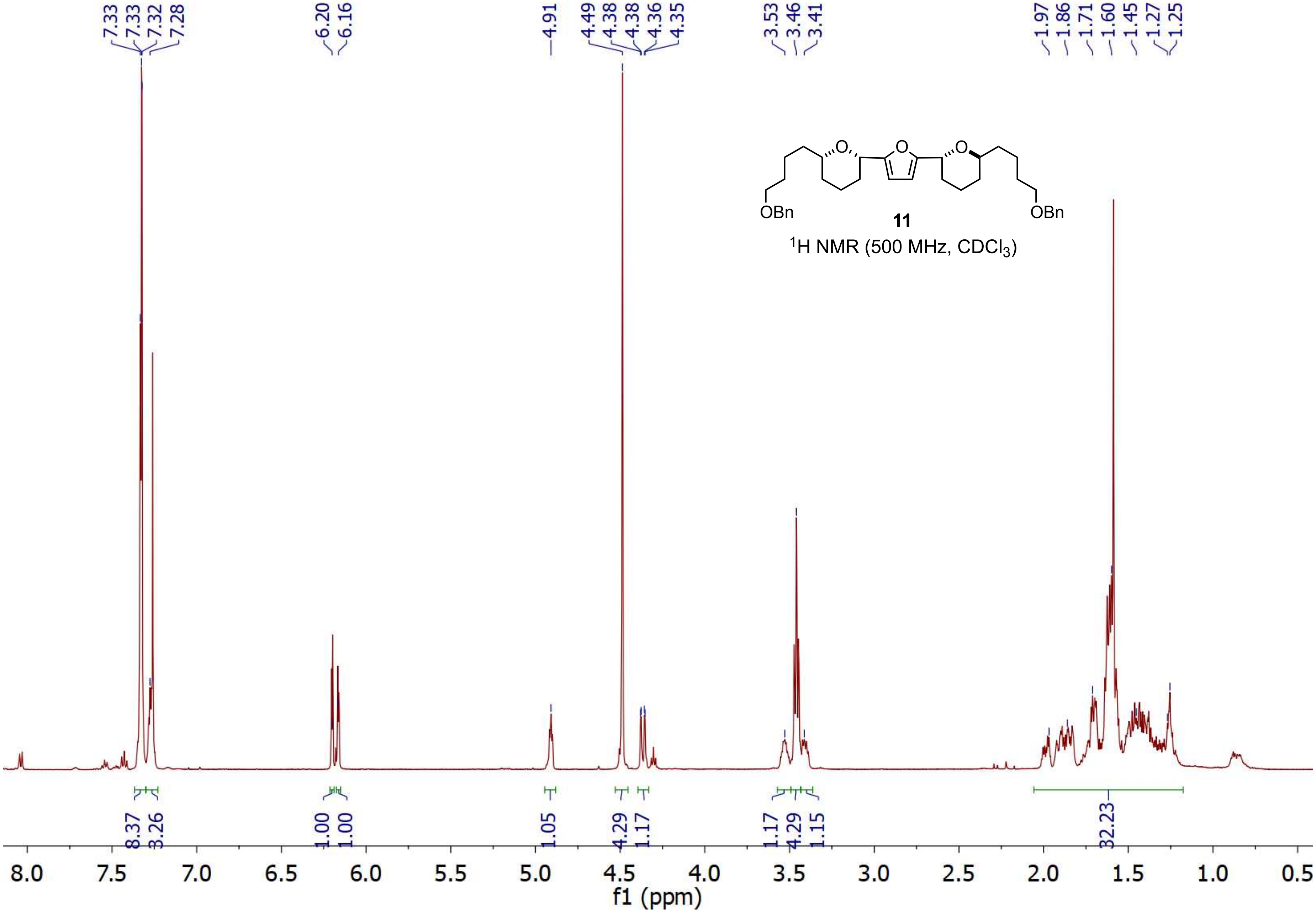
7.33
7.33
7.32
7.28
6.20
6.16
4.91
4.49
4.38
4.38
4.36
4.35
3.53
3.46
3.41
1.97
1.86
1.71
1.60
1.45
1.27
1.25
OBn
OBn
11
^{1}H NMR (500 MHz, CDCl$_3$)
8.37
3.26
1.00
1.00
1.05
4.29
1.17
1.17
4.29
1.15
32.23
f1 (ppm)

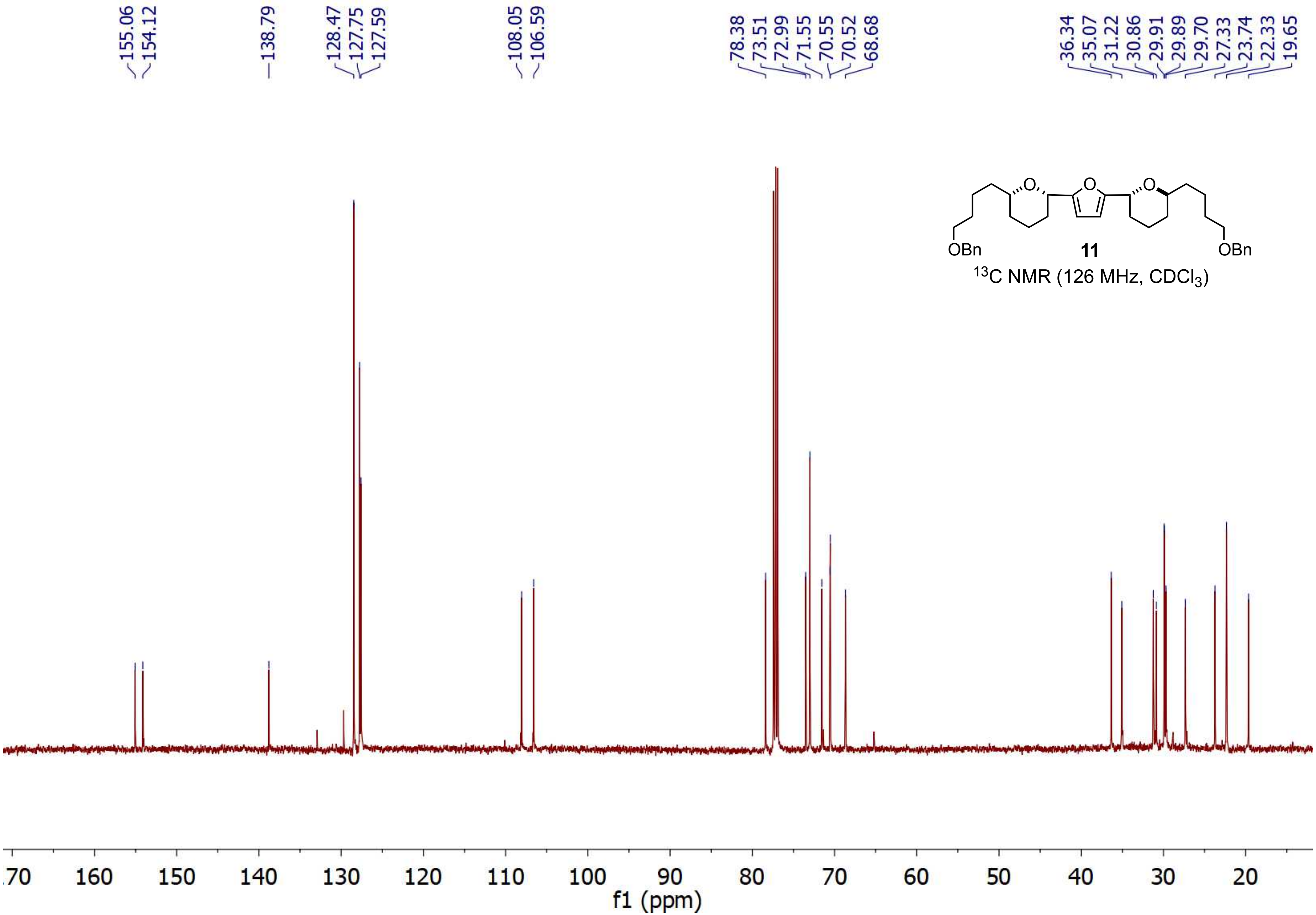
155.06
154.12
138.79
128.47
127.75
127.59
108.05
106.59
78.38
73.51
72.99
71.55
70.55
70.52
68.68
36.34
35.07
31.22
30.86
29.91
29.89
29.70
27.33
23.74
22.33
19.65
OBn
OBn
11
^{13}C NMR (126 MHz, $CDCl_3$)
f1 (ppm)

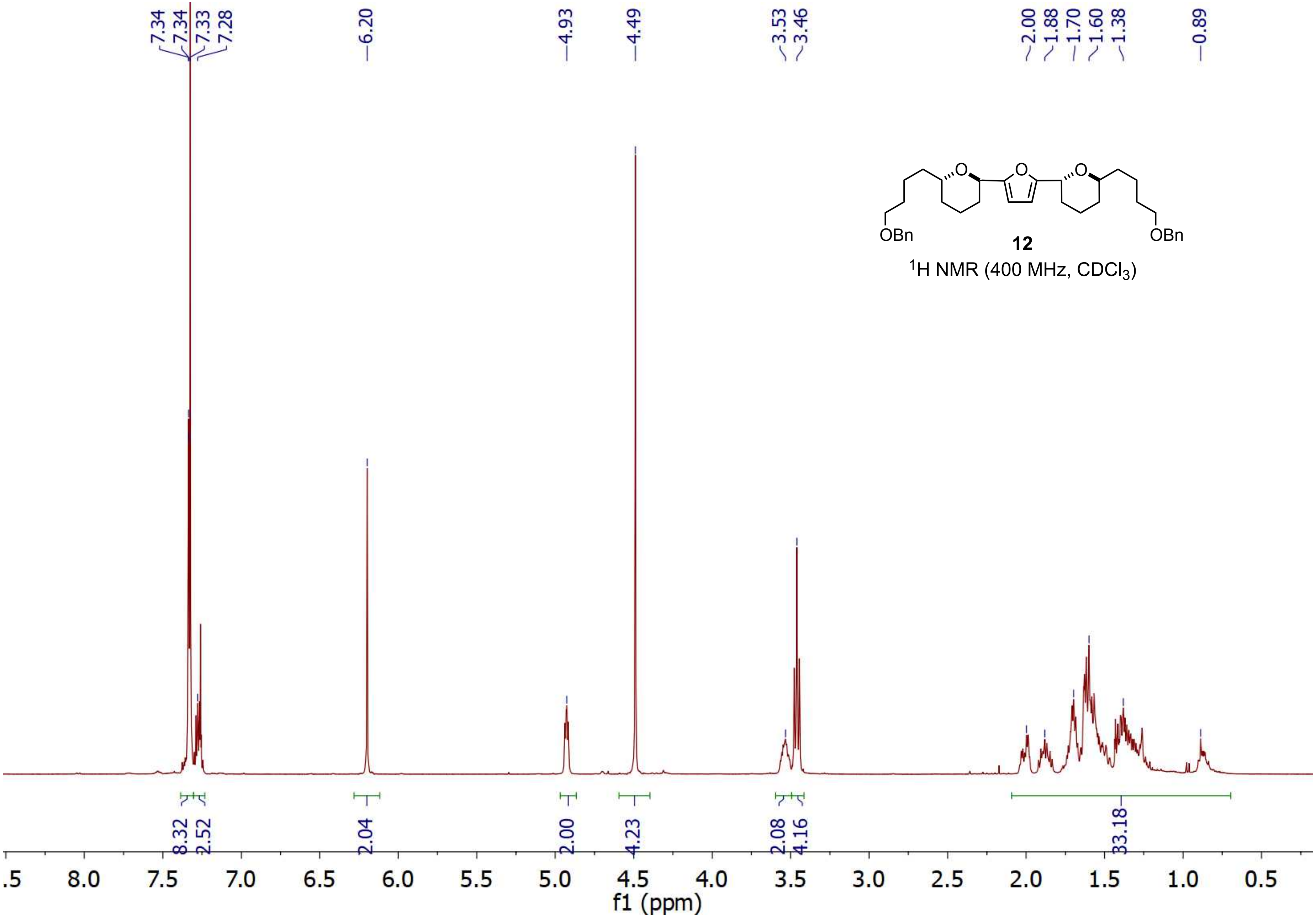
7.34
7.34
7.33
7.28
6.20
4.93
4.49
3.53
3.46
2.00
1.88
1.70
1.60
1.38
0.89
OBn
OBn
O
O
O
12
^{1}H NMR (400 MHz, CDCl$_3$)
8.32
2.52
2.04
2.00
4.23
2.08
4.16
33.18
.5
8.0
7.5
7.0
6.5
6.0
5.5
5.0
4.5
4.0
3.5
3.0
2.5
2.0
1.5
1.0
0.5
f1 (ppm)

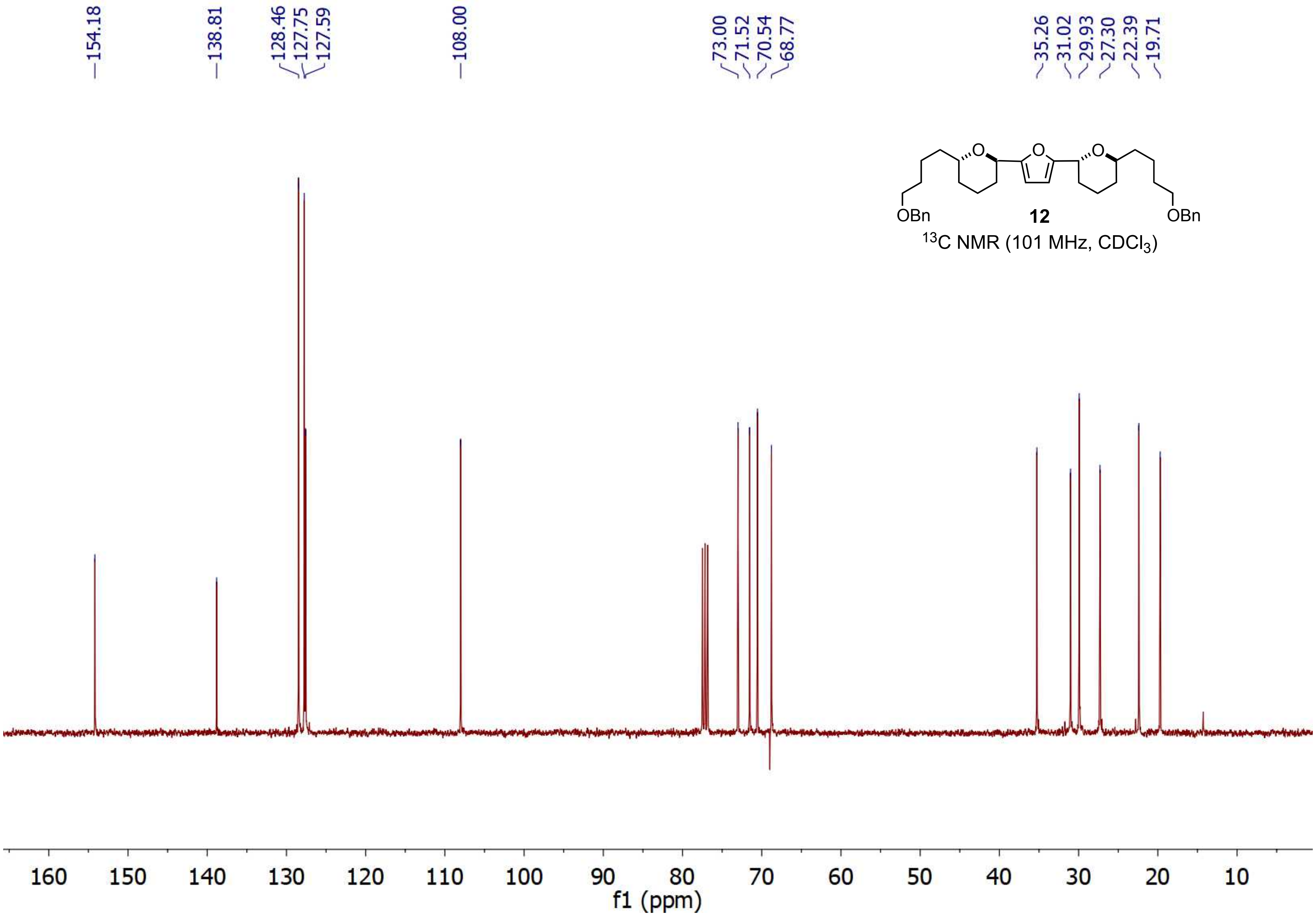

154.18
138.81
128.46
127.75
127.59
108.00
73.00
71.52
70.54
68.77
35.26
31.02
29.93
27.30
22.39
19.71
OBn
12
OBn
^{13}C NMR (101 MHz, $CDCl_3$)
160 150 140 130 120 110 100 90 80 70 60 50 40 30 20 10
f1 (ppm)

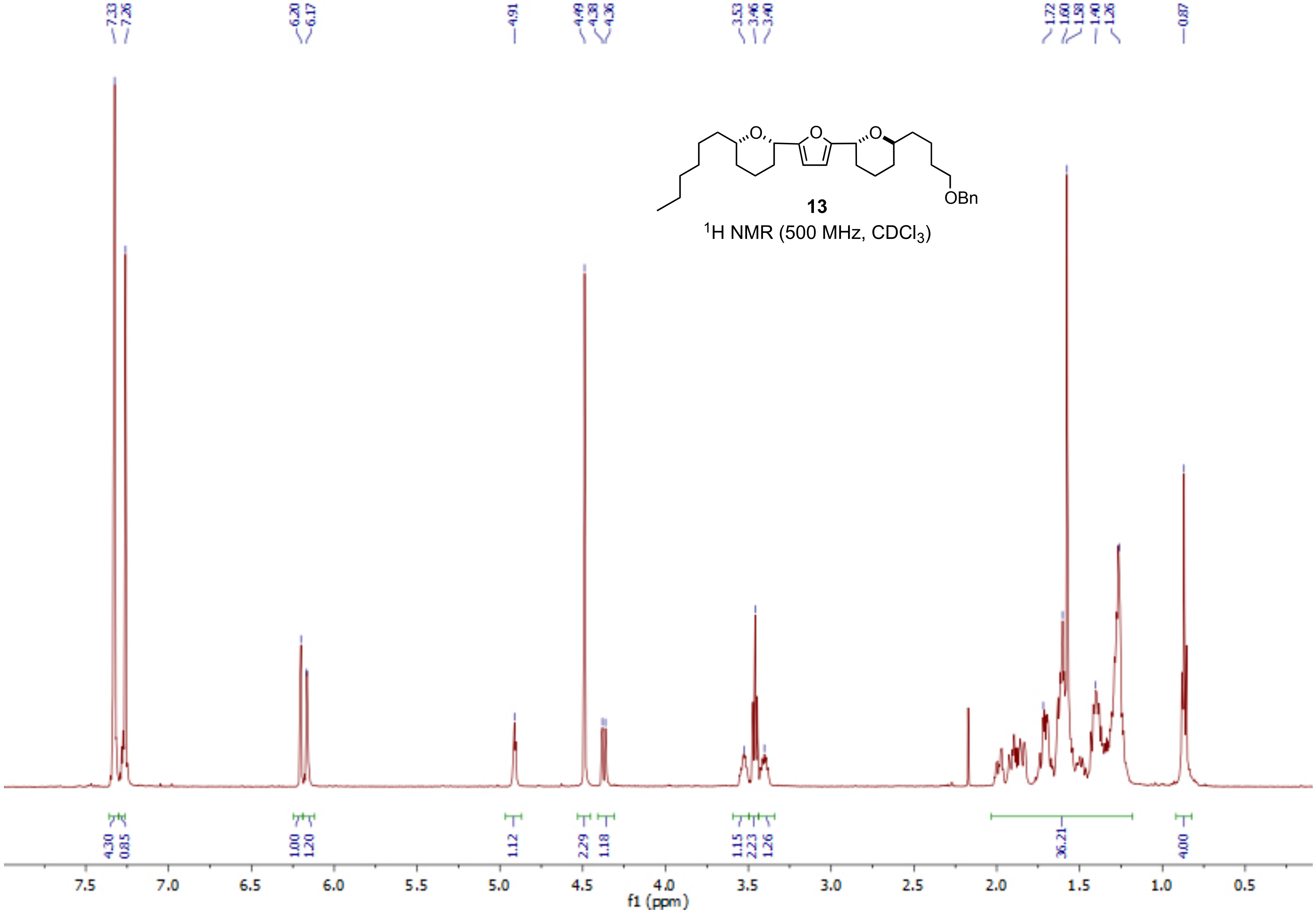
13
^{1}H NMR (500 MHz, $CDCl_3$)
OBn
f1 (ppm)

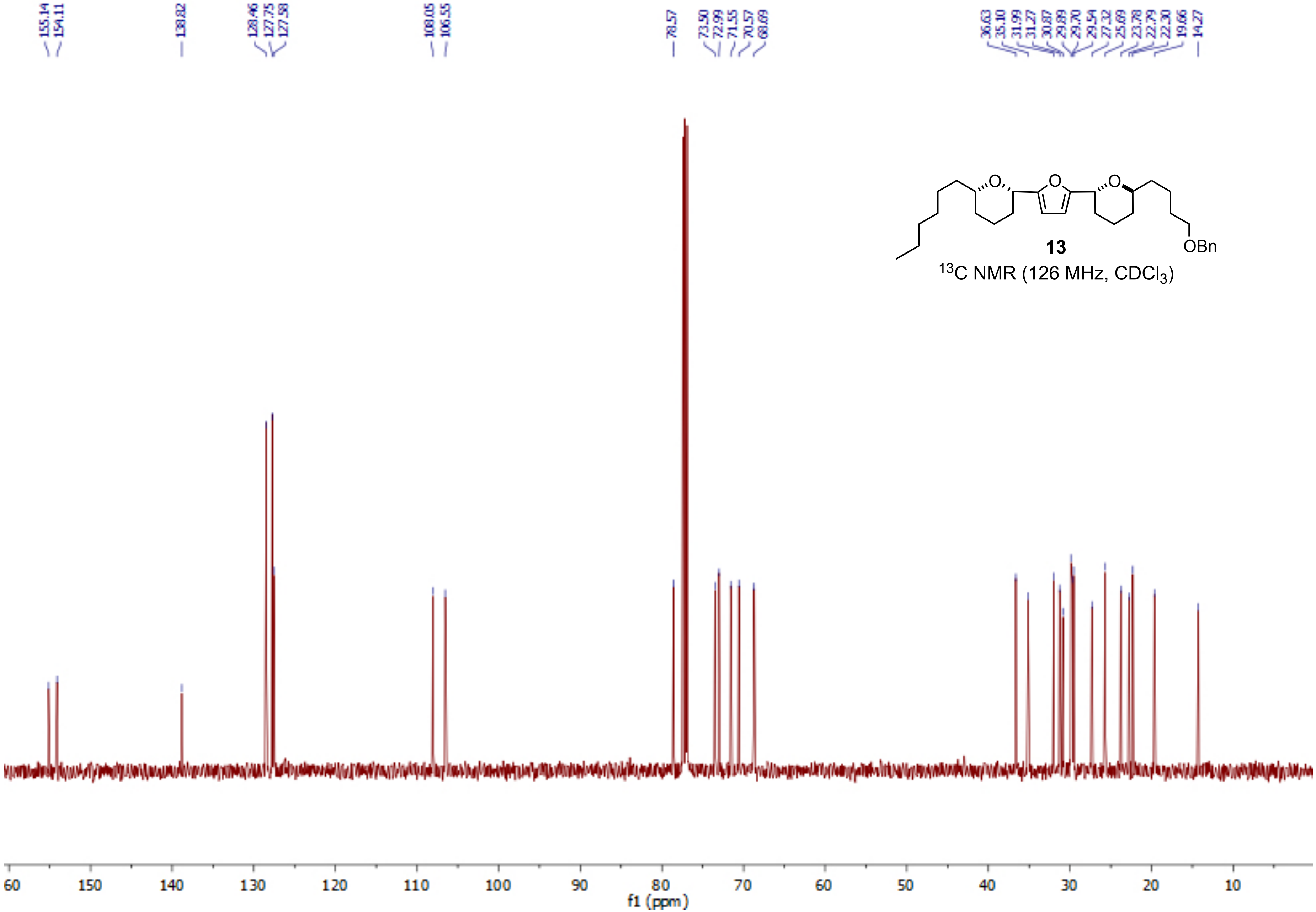
155.14
154.11
138.82
128.46
127.75
127.58
108.05
106.55
78.57
73.50
72.99
71.55
70.57
68.69
36.63
35.10
31.99
31.27
30.87
29.89
29.70
29.54
27.32
25.69
23.78
22.79
22.30
19.66
14.27
O
O
O
OBn
13
^{13}C NMR (126 MHz, $CDCl_3$)
f1 (ppm)
